# Gene activity in primary T cells infected with HIV_89.6_: intron retention and induction of genomic repeats

**DOI:** 10.1186/s12977-015-0205-1

**Published:** 2015-09-17

**Authors:** Scott Sherrill-Mix, Karen E. Ocwieja, Frederic D. Bushman

**Affiliations:** Department of Microbiology, Perelman School of Medicine at the University of Pennsylvania, 425 Johnson Pavilion, 3610 Hamilton Walk, Philadelphia, PA 19104 USA; Children’s Hospital of Philadelphia, 3401 Civic Center Blvd., Philadelphia, PA 19104 USA

**Keywords:** HIV, T cell, RNA-Seq, Transcription, Splicing, Expression, Chimera, HERV, Retrotransposons

## Abstract

**Background:**

HIV infection has been reported to alter cellular gene activity, but published studies have commonly assayed transformed cell lines and lab-adapted HIV strains, yielding inconsistent results. Here we carried out a deep RNA-Seq analysis of primary human T cells infected with the low passage HIV isolate HIV_89.6_.

**Results:**

Seventeen percent of cellular genes showed altered activity 48 h after infection. In a meta-analysis including four other studies, our data differed from studies of HIV infection in cell lines but showed more parallels with infections of primary cells. We found a global trend toward retention of introns after infection, suggestive of a novel cellular response to infection. HIV_89.6_ infection was also associated with activation of several human endogenous retroviruses (HERVs) and retrotransposons, of interest as possible novel antigens that could serve as vaccine targets. The most highly activated group of HERVs was a subset of the ERV-9. Analysis showed that activation was associated with a particular variant of ERV-9 long terminal repeats that contains an indel near the U3-R border. These data also allowed quantification of >70 splice forms of the HIV_89.6_ RNA and specified the main types of chimeric HIV_89.6_-host RNAs. Comparison to over 100,000 integration site sequences from the same infected cell populations allowed quantification of authentic versus artifactual chimeric reads, showing that 5′ read-in, splicing out of HIV_89.6_ from the D4 donor and 3′ read-through were the most common HIV_89.6_-host cell chimeric RNA forms.

**Conclusions:**

Analysis of RNA abundance after infection of primary T cells with the low passage HIV_89.6_ isolate disclosed multiple novel features of HIV-host interactions, notably intron retention and induction of transcription of retrotransposons and endogenous retroviruses.

**Electronic supplementary material:**

The online version of this article (doi:10.1186/s12977-015-0205-1) contains supplementary material, which is available to authorized users.

## Background

HIV replication requires integration of a cDNA copy of the viral RNA genome into cellular chromosomes, followed by transcription and splicing to yield viral mRNA. Alternative splicing allows the small 9.1 kb HIV genome to generate at least 108 mRNA transcripts encoding at least 9 proteins and polyproteins [1–6]. During replication, HIV also reprograms cellular transcription and splicing. For example, the virus-encoded Vpr protein arrests the cell cycle [7–10] and the viral Tat protein binds to P-TEFb and alters transcription at the HIV promoter and some cellular promoters as well [11–16].

Changes in host cell gene expression have been reported during HIV infection [17–29] and differences in expression have been observed associated with the stage [30] and progression [31] of disease. Multiple studies suggest that cells detect HIV infection, in part through the recognition of cytoplasmic DNA in abortive infections [32–34], and respond by inducing interferon-regulated, apoptotic and stress response pathways [18–22, 25–28]. Several studies have also suggested that HIV infection disrupts normal cellular splicing pathways [28, 35]. However, results have varied with many experimental parameters, including target cell type, HIV isolate and the duration of infection. Many previously published studies have focused on infections with lab-adapted HIV strains in transformed cell lines [17, 18, 24, 25, 28, 29, 36], and so results may not be fully reflective of infections in patients.

HIV infection also appears to induce the expression of human endogenous retroviruses (HERVs) [37], particularly HERV-K [38–42], and retrotransposons [43]. Immune responses to HERV proteins appear stronger in HIV-infected individuals suggesting candidate markers of infection and possible vaccine targets [44–47]. In contrast, two recent RNA-Seq studies of expression during HIV infection did not report increases in HERV RNA [24, 25]. The origin of this discrepancy is unclear.

The suggestion that HIV integration may disrupt cellular cancer-associated genes and thereby promote cell proliferation [48–51] has focused attention on the range of novel message types formed when HIV integrates within transcription units [52–56]. Chimeric reads containing HIV and cellular sequence are also of interest due to the potential of lentiviral vectors to trigger oncogenesis in gene therapy patients through insertional mutagenesis [57–60]. A better understanding of chimera formation would help clarify this phenomenon in both HIV infections and lentiviral vector-based gene therapies.

In this study, we sought to generate data more representative of HIV replication in patients by using Illumina sequencing to analyze transcriptional responses after infection of primary T cells with HIV_89.6_, a low passage patient isolate [61]. This represents a continuation of a long term effort to understand HIV-host cell interactions at the transcriptional level that began with analysis of transcription by HIV_89.6_ in primary T cells using Pacific Biosciences long read single molecule sequencing [6]. Our strategy here was to analyze a single time after infection in depth with over one billion sequence reads from HIV_89.6_-infected and uninfected host cells. These data were then combined with 147,281 unique integration site sequences from the same infections and the Pacific Biosciences data on HIV_89.6_ transcription to (1) elucidate effects of HIV infection on host cell mRNA abundances and splicing, (2) characterize viral message structure in detail and (3) probe the nature of the chimeras formed between host cell and viral RNAs.

## Results

### Infections studied

Primary CD4^+^ T cells from a single human donor were infected with HIV_89.6_, a clade B primary clinical isolate [61], in three replicates. For comparison, two additional replicates from the same donor were mock infected. Samples were harvested 48 h after viral inoculation, which allowed for widespread infection in the primary T cell cultures, though some cells may have been infected secondarily by viruses produced in the first round. Thus cultures probably were not tightly synchronized but did have extensive representation of infected primary T cells. From these samples, we obtained 1,161,705,678 101 bp reads; 1,021,207,853 were mapped to the human genome and 24,783,844 to the HIV_89.6_ provirus (Table 1). Below we first discuss the influence of infection on cellular gene activity and RNA splicing, then analyze HIV RNAs and lastly identify chimeras formed between HIV and cellular RNAs.

**Table 1:** Samples and sequencing coverage

Sample	Infection rate (%)	Reads	Human reads	HIV reads	% HIV	% HIV in infected
Uninfected-1	–	232,450,106	212,391,460	–	–	–
Uninfected-2	–	235,048,212	203,760,783	–	–	–
Infected-1	37.5	234,378,088	199,871,662	10,219,315	4.86	13.0
Infected-2	26	226,078,422	198,436,507	7,322,556	3.56	13.7
Infected-3	21	233,750,850	205,747,441	7,241,973	3.40	16.2

Samples used in this study, their infection rates and sequencing depth. “% HIV in infected” is an estimate based on the assumption that infected and uninfected cells contain equal amounts of mRNA

### Changes in gene activity in primary T cells upon infection with HIV_89.6_

We observed significant expression changes in 3,142 genes (false discovery rate of $q<0.01$), which is 17.1 % of expressed cellular genes (Additional file 1). The genes with most extreme increases, all $>6\times$ fold higher, during HIV infection included IFI44L, RSAD2, HMOX1, MX1, USP18, IGJ, OAS1, CMPK2, DDX60, IFI44, IFI6, IFNG and CCL3. All of these have been reported to be involved in innate immunity [62] or are interferon-inducible [63], highlighting a strong innate immune response in the cells studied. Genes with the largest decreases, all $>3\times$ fold lower, were GNG4, GPA33, IL6R, CCR8, RORC, AFF2 and CCR2.

Many Gene Ontology [64] categories were significantly enriched for differentially expressed genes (Additional file 2). Notably upregulated with infection were genes involved in apoptosis, immune responses and cytokine production (all $q<10^{-4}$) and downregulated were ribosomal protein genes and related pathways ($q<10^{-15}$). These changes suggest that the cells responded to HIV infection with the induction of inflammatory, interferon-regulated and apoptotic responses, patterns posited from several previous studies [18–22, 24–27, 29, 36, 65]. Expression significantly increased for several genes that are characteristic of other hematopoietic lineages, e.g. hemoglobin $\beta$, CD8, CD20 and CD117, while several CD4^+^ T cell specific genes, e.g. CD4 and CD3, were downregulated, potentially consistent with de-differentiation of infected or bystander cells. We return to this point in the discussion.

### Comparison of transcriptional profiles from HIV_89.6_ infection of primary T cells to data on HIV infection in other cell types

We sought to identify the transcriptional responses that were most conserved upon HIV infection and so collected and analyzed data from four other studies of transcription in HIV-infected cells (Additional file 3). These included two studies of infection of the SupT1 cell line [24, 25], a study of ex vivo infection of primary CD4^+^ T cells [26] and a study of lymphatic tissue biopsies from acutely viremic patients [30]. Genes were scored as increased or decreased in activity in infected cell populations, and the amount of agreement was compared among the different studies.

No gene was called as differentially expressed in all five studies. Eight genes were differentially expressed in the same direction in 4 out of 5 studies; AQP3 and EPHX2 were downregulated with HIV infection and CD70, EGR1, FOS, ISG20, RGS16 and SAMD9L were upregulated. A full listing is provided in Additional file 4. Several of the upregulated genes are known to be interferon-inducible, again emphasizing the role of innate immune pathways.

**Fig. 1:**
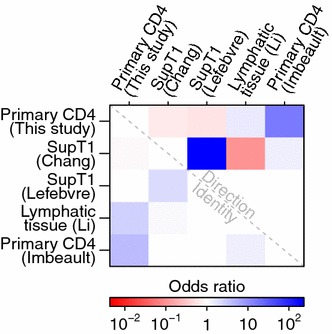
Comparisons among studies quantifying cellular gene expression after HIV infection. For each pair of studies, the association between up- and downregulation calls was measured for genes identified by both studies as differentially expressed (above the *diagonal*). As another comparison, we also measured the agreement between studies for which genes were called differentially expressed regardless of direction (below the *diagonal*). The *color scale* shows the conservative (i.e. closest to 1) boundary of the confidence interval of the odds ratio with *blue* indicating a positive association and *red* a negative association between studies. For confidence intervals overlapping 1, the value was set to 1. Therefore all *colored squares* indicate significant associations

For each pair of studies, we compared whether they agreed on the identities of differentially expressed genes and whether they agreed on the direction of change (Fig. 1). The responses to infection in primary cells showed notable differences to responses in the SupT1 cell line. The two SupT1 studies were significantly similar to each other (odds ratio: 1090, 95 % confidence interval (CI) 232–16,400, Fisher’s exact test $p<10^{-15}$ for direction of change in differentially expressed genes) but were not significantly associated or were negatively associated with data from ex vivo primary cells and from lymphatic tissue from acutely infected HIV patients. In contrast, our data was significantly associated with the primary cell (odds ratio: 75.7, 95 % CI 16.9–701, $p<10^{-15}$) and lymphatic tissue data (odds ratio: 6.49, 95 % CI 1.52–24.9, $p=0.003$). This documents significant differences in responses to HIV infection between infected primary cells and SupT1 cells and suggests that results of infections in primary cells more closely align with actual acute HIV infections in patients. SupT1 cells might be expected to respond to infection differently than primary cells since they have several nonsynonymous mutations in innate immunity genes [66], have blocks in immune signaling pathways [67] and fail to activate many interferon-stimulated genes during HIV infection [27].

### Comparison of the HIV-infected cell transcriptional profile to additional experimental T cell profiles

**Fig. 2:**
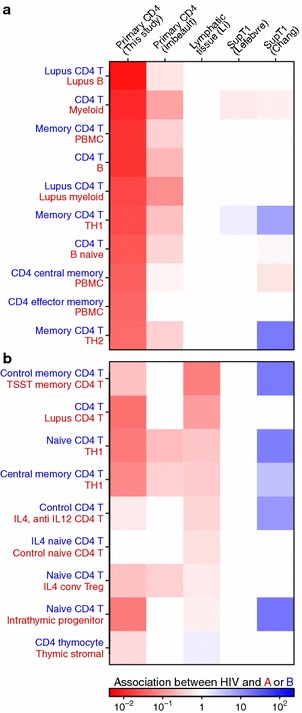
Comparisons of the effect of HIV infection on cellular gene expression to additional studies comparing transcription in subsets of immune cells. The MSigDB database was used to extract 185 sets of differentially expressed genes from pairs of transcriptional profiling studies of immune cell subsets involving CD4^+^ T cells. For each pair of studies, we used Fisher’s exact test to measure the association between up- and downregulation calls for genes identified as differentially expressed in both our HIV study and the comparator immune subsets. **a** The transcriptional profiles with strongest associations with changes observed in our study of HIV_89.6_ infection of primary T cells. *Blue* indicates a positive association between changes seen in HIV-infected cells and the first immune subset (*text colored blue*) while *red* indicates a positive association with the second immune subset (*text colored red*). The *color scale* shows the conservative (i.e. closest to 1) boundary of the confidence interval of the odds ratio. For confidence intervals overlapping 1, the value was set to 1. Therefore all *colored squares* indicate significant associations. **b** As in **a**, but showing the transcriptional profiles most strongly associated with changes observed in lymph node biopsies from acutely infected patients [30]

To investigate the transcriptional changes in more depth, we compared the results of the five studies of HIV infection to transcriptional profiles comparing immune cell subsets available at the Molecular Signatures Database (MSigDB) [68]. The MSigDB reports genes that are increased or decreased in relative expression for 185 pairs of transcriptional profiles involving CD4^+^ T cells. We compared the lists of affected genes in each pair to genes altered in activity by HIV infection. Those pairs of studies with the most significant associations with HIV_89.6_ data are shown in Fig. 2a. For comparison, the associations with the four other HIV transcriptional profiling studies mentioned above are shown as well.

The most significant associations for our data showed gene expression in HIV_89.6_-infected cells moving away from typical T cell expression patterns and towards patterns more similar to B cells, myeloid cells and bulk peripheral blood mononuclear cells (all Fisher’s exact test $p<10^{-15}$) (Fig. 2a). These changes were also seen, although to a lesser extent, in the Imbeault et al. [69] study which also used primary CD4^+^ T cells.

For comparison, we also extracted those profiles most strongly associated with the transcriptional data on lymphatic tissue of HIV patients [30]. The profiles showed patterns similar to strongly stimulated T cells, autoimmune disease and to the Th1 T cell subset (all $p<0.01$) (Fig. 2b). Our data in primary CD4^+^ T cells paralleled the changes seen in lymphatic tissue. These transcriptional changes again highlights the strong immune response generated by HIV infection in primary cells.

### Intron retention

**Fig. 3:**
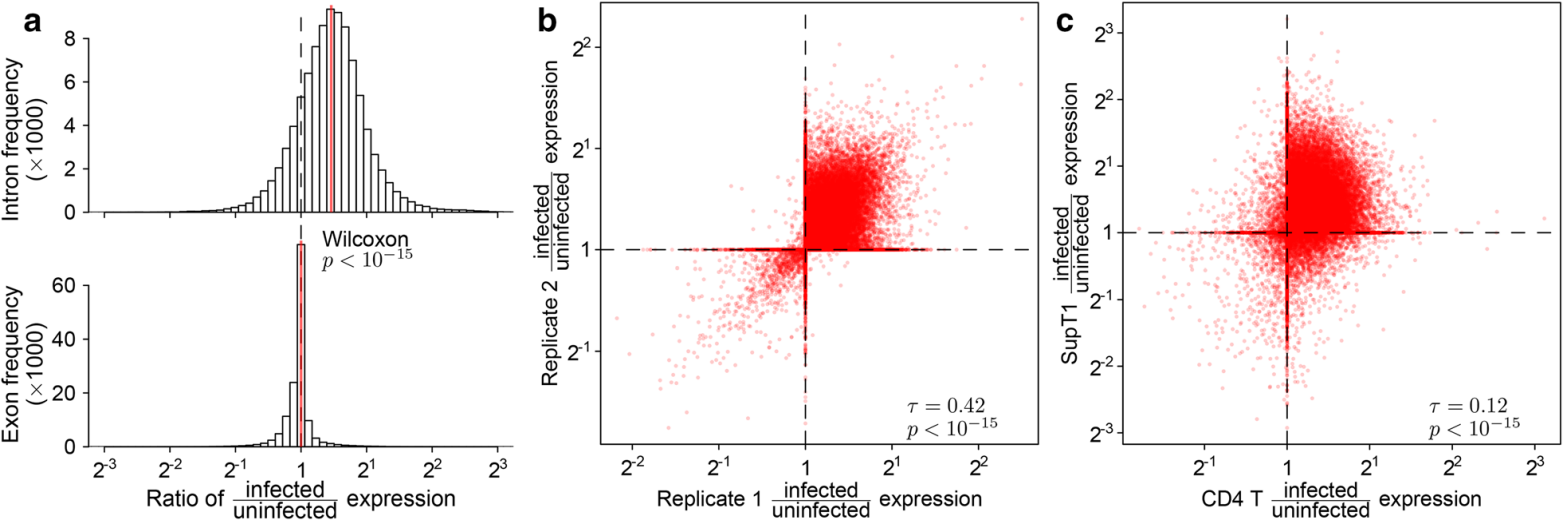
Changes in the abundance of intronic regions with HIV infection. Expression of intronic and exonic regions was quantified as the proportion of reads mapping within the intron/exon out of the total reads mapping to the transcription units overlapping that intron/exon. **a** Comparison of the ratios of expression between infected and uninfected replicates in exclusively intronic or exonic regions of transcription units. **b** Reproducibility of intron retention between replicates. Each *point* quantifies the change in expression with HIV infection for a specific intronic region. The x-axis shows changes in gene activity accompanying infection for one set of replicates (Infected-1 and Infected-2 vs. Uninfected-1) and the y-axis shows the same data for different replicates (Infected-3 vs. Uninfected-2). **c** Reproducibility of intron retention between studies. The *plot* is arranged as in **b** but with all data from our study combined on the x-axis and corresponding data from Chang et al. [25] on the y-axis

Cells respond to infection by shutting down macromolecular synthesis at multiple levels [70–74], so we investigated whether cells also showed perturbations in splicing efficiency after infection. As a probe, we created a database of cellular genomic regions annotated exclusively as exons or introns in all splice forms in the UCSC gene database [75] and quantified expression in these regions in infected and uninfected cells. We found a significant increase in intronic sequences relative to exonic sequence (Wilcoxon test $p<10^{-15}$) (Fig. 3a). This increase in intronic sequence was reproducible between replicates in our study (Kendall’s $\tau =0.42$, $p<10^{-15}$) (Fig. 3b). We reanalyzed RNA-Seq data from Chang et al. [25] and also documented intron retention that correlated with the changes seen in our data (Kendall’s $\tau =0.12$, $p<10^{-15}$) (Fig. 3c).

A possible artifactual explanation for enrichment of intronic sequences could involve greater DNA contamination in the infected cells samples. That is, if the relative amount of DNA differed between treatments, the amount of apparent intronic sequences could also differ due to sequencing of contaminating DNA. To examine whether DNA contamination was abundant in our samples, we compiled a collection of 27 large gene desert regions, defined here as (1) regions outside the centrosome and first and last cytoband, (2) containing less than 1 % unknown sequence, (3) containing no genes annotated in UCSC genes [75], (4) containing no repeats annotated in the RepeatMasker database [76] and (5) spanning more than 100 kb. No reads were mapped to these 41 Mb of gene deserts in any sample, arguing against explanations based on DNA contamination. Thus these data indicate that intron retention was increased in these cell populations upon HIV infection, revealing a previously undisclosed aspect of the host cell transcriptional response to infection.

Ribosomal protein genes were especially enriched for introns with strong increases in expression with HIV infection (odds ratio: 55.5, 95 % CI 36.9–81.5, Benjamini–Hochberg corrected Fisher’s exact test $q<10^{-15}$ for introns with a Bayesian 95 % credible interval for differential abundance of $>2\times$ change). Intron retention was not restricted to particular introns but was evident in most introns in affected genes (Additional file 5A). No other Gene Ontology category had a $q<0.01$ after excluding introns from ribosomal protein genes.

Intron retention has been linked to intronic characteristics such as splice site strength, GC content and intron width across many cell types [77]. To see if a similar pattern existed in our data, we fit a lasso-regularized logistic regression [78] to predict differential expression of an intronic region based on GC content, width and 3′ and 5′ splice site strength [79] of the introns overlapping the region. We also included a term indicating whether the intron was in a ribosomal protein gene and, because HIV has been reported to induce the expression of HERVs and other repetitive elements, a term indicating if the intron contained any repeat annotation in the RepeatMasker database. The resulting model selected only whether the intron contained a repetitive element and whether it was in a ribosomal protein gene and reduced cross-validated mean square error by only 1 %. Thus, it appears that the intron retention induced by HIV infection does not follow the same patterns seen when comparing cell types and that much of the variation in HIV-induced intron retention remains unexplained.

### Induction of transcription from HERVs and retrotransposons by HIV_89.6_ infection

**Fig. 4:**
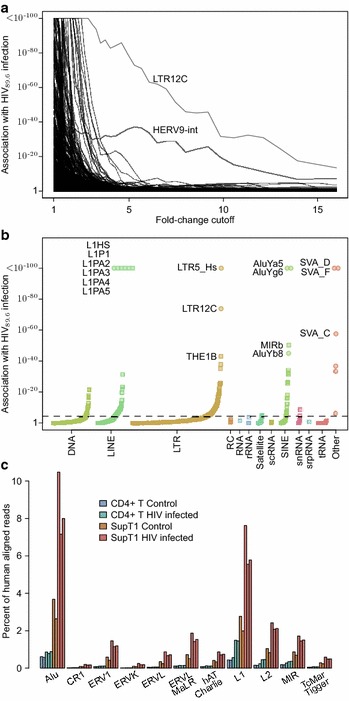
Repeat categories enriched upon infection with HIV. **a** The association of repeat regions differentially expressed after HIV_89.6_ infection of primary T cells observed for varying thresholds of differential expression. The threshold used to call a gene differentially expressed based on the Bayesian posterior median was varied and Fisher’s exact test was used to assess whether any genomic repeats had a significant association with this differential expression. Note that only ERV-9 (annotated as HERV9-int in the RepeatMasker database) and it’s corresponding long terminal repeat ERV-9/LTR12C were significantly associated with large changes in expression. **b** Enrichment of repeat categories in regions differentially expressed (Bayesian 95 % credible interval $>2\times$ change) between HIV-infected and control CD4^+^ T cells. The repeated sequences are ordered on the x-axis by the extent of induction within each class with *circles* indicating repeats annotated as hominid specific and *squares* marking all other repeats, the y-axis shows the *p* value for upregulation after infection. The *dashed line* indicates a Bonferroni corrected *p* value of 0.05. **c** The proportion of human mapped reads that align within classes of genomic repeats for data from primary CD4^+^ T cells from this study and SupT1 cells from Chang et al. [25]. A single read mapping multiple times to a given category was only counted once

Because some differentially expressed introns appeared associated with repetitive elements, we investigated the expression of HERVs, transposons and other repeated sequences. Figure 4a shows a comparison of the association between changes in expression with HIV_89.6_ infection and genomic repeat types annotated in the RepeatMasker database [76] over varying levels of differential expression. At high levels of expression change, ERV-9 (odds ratio: 154, 95 % CI 83.1–262, $p<10^{-15}$ for LTRs with a Bayesian 95 % credible interval for differential abundance $>4\times$ change) and its long terminal repeat LTR12C (odds ratio: 145, 95 % CI 98.9–210, $p<10^{-15}$) are the only repeats highly associated with HIV infection. Looking at genomic repeats with any significant increase during HIV infection, the expression of many recently acquired genomic repeats, including L1HS, LTR5_Hs (a human specific long terminal repeat of HERV-K), AluYa5, AluYg6 and SVA_D and SVA_F, were associated with HIV_89.6_ infection (Fig. 4b).

We saw a relationship between the age of genomic repeats and its likelihood of being induced by HIV_89.6_ infection. The most highly enriched repeats were associated with relatively recent hominid-specific repeat classes as annotated by the RepeatMasker database (repeat classes with $p<10^{-50}$ odds ratio: 31.6, 95 % CI 8.88–112, $p=10^{-7}$). In HERV-K (HML-2), the most recently active endogenous retrovirus in the human genome [80–82], we saw that integrations unique to the human genome [82] were more likely to be differentially expressed than older HERV-Ks (odds ratio: 5.38, 95 % CI 1.93–16.0, $p=0.0005$).

Previous RNA-Seq studies of cellular expression during HIV infection in transformed cell lines did not report increases in HERV mRNA [24, 25]. To investigate this difference, we downloaded and analyzed the RNA-Seq data from Chang et al. [25], which quantified gene activity in transformed SupT1 cells infected with a lab-adapted strain of HIV. We found a much higher level of HERV expression in their data in both HIV-infected cells and uninfected controls than in primary cells (Fig. 4c). We suspect that in SupT1 cells, as with many cancerous cells [83–87], the baseline expression of transposons and endogenous retroviruses is higher than in primary cells, masking further induction by HIV infection.

**Fig. 5:**
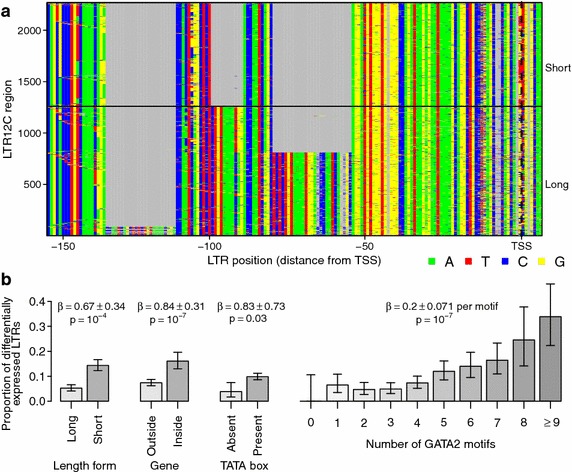
Characteristics of ERV-9/LTR12C sequences associated with induction upon infection of primary T cells with HIV_89.6_.** a** An alignment of the 3′ end of the U3 region of repeats annotated as ERV-9/LTR12C. Each* row* is a section of the long terminal repeat sequence and each* column* a base in that sequence colored by nucleotide identity. For clarity, positions appearing in less than 2 % of sequences are omitted. Two distinct classes are visible with a short form and long forms containing varying numbers of repeated sequence. Mutations away from the consensus can also be seen.** b** The proportion of LTR12C regions with significant increases in read abundance after infection with HIV and their 95 % confidence intervals separated by the length class of the LTR, presence in a gene, presence of a TATA box and the number of GATA2 motifs in the U3 region. These variables were selected by stepwise regression regression comparing differential expression of LTR12C to the length class of the LTR, the number of mutations away from consensus, the number of NFY, GATA2 and MZF1 motifs and the presence of a TATA box motif within 50 bp of the transcription start site. Variables are labeled with the estimated change in log odds ratio ($\beta$) and their Wald test *p* values

We observed heterogeneous expression among ERV-9/LTR12C sequences and so investigated the primary sequence determinants. We observed that LTR12C has variants with differing number of repeated sequence in the U3 region just upstream of the transcription start site (Fig. 5a), an important region for transcription initiation [88]. The U3 region of LTR12C also contains multiple motifs for transcription factors NFY, GATA2 and MZF1 [89]. To clarify factors affecting expression levels, we counted the number of motifs matching these transcription factors’ binding motifs, checked for a TATA box [90] within 50 bp upstream of the transcription start site, assigned each LTR12C to the short or long length class, counted the number of mutations away from the consensus for that length class and checked for integration in a transcription unit. We then applied a logistic regression to test the effects of these variables on LTR12C differential expression. We found that HIV_89.6_-induced transcription was more likely for LTR12C containing the short length variant of the 3′ U3 region, located within a transcription unit, containing a TATA box motif and containing greater numbers of GATA2 motifs (Fig. 5b).

Transcription extending several hundred kilobases from several ERV-9/LTR12C has recently been reported [91]. In contrast in our data, only 14 LTR12C appeared to have continuous transcription more than 1000 bp downstream of the LTR and the maximum length of continuous transcription was only 9275 bp. Transcription from some of these LTR12C does appear to continue directly into transcription units of cellular genes, suggestive of the potential for regulatory function (Additional file 5B).

### HIV mRNA synthesis and splicing

Over 24 million Illumina reads mapped to HIV_89.6_, yielding an average coverage of over 240,000-fold. Reads mapping to HIV_89.6_ comprised between 3.4–4.8 % of mapped reads in the infected samples (Table 1). It is unclear whether HIV infection increases or decreases the amount of mRNA in infected cells but if we assume HIV-infected cells contain the same amount of mRNA as uninfected cells and adjust for rates of infection ranging between 21–37.5 % (Table 1), we estimate that HIV transcripts comprise between 13.0–16.2 % of the total polyadenylated mRNA nucleotides in infected cells 48 h after initial infection. This parallels previous estimates of around 10 % [92] at 48 h postinfection, 38 % at 24 h [25] or 30 % after 72 h [18].

**Fig. 6:**
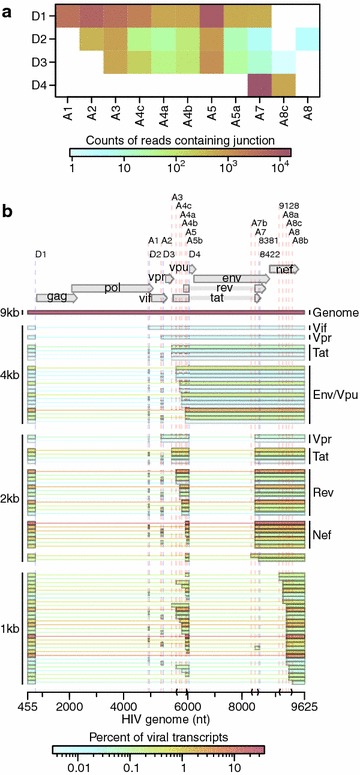
Transcription and splicing of the HIV_89.6_ RNA.** a** Junctions between HIV splice donors and acceptors observed in the RNA-Seq data. Acceptors are shown as the* columns* and donors as the* rows* with the* coloring* indicating the frequency of each pairing.** b** The relative abundance of 78 HIV_89.6_ transcripts as determined by a combination of PacBio sequencing [6] and Illumina sequencing. Message structures were generated by targeted long read single molecule sequencing, which allowed association of multiple splice junctions in single sequence reads. The Illumina short read sequencing allowed normalization of message abundances between size classes. The inferred HIV message population is shown colored by relative abundance

Over 47,257 single reads spanned previously reported HIV splice junctions, allowing a quantitative assessment of donor and acceptor utilization (Fig. 6a). As expected from previous studies [4, 6], the most abundant junctions were D1-A5 and D4-A7. We confirmed the use of unusual splice acceptors A8c and A5a, previously reported in HIV_89.6_ [6]. In the Illumina sequencing, we saw a higher abundance of D1-A1 and D1-A2 splice junctions than in PacBio sequencing [6], possibly indicative of recovery bias in PacBio sequencing.

A 3′ bias is apparent in our sequencing data (Additional file 6A). This could be due to the poly-A capture step of the protocol where any break in the RNA would result in loss of distal 5′ sequences [93]. We used sequence reads from the large unspliced HIV intron 1 to measure this bias by regressing the $\log$ of the number of fragments with a 5′-most end starting at a given position against the distance of that position from the viral polyadenylation site, yielding an estimated probability of breakage of 0.021 % per base (Additional file 6A). Given this rate of truncation, there is only a 14 % chance of reaching the 5′ end of the 9171 nt unspliced HIV genome ($(1-0.00021)^{9171}$).

Ocwieja et al. [6] determined the relative abundance of HIV_89.6_ of similarly sized transcripts using PacBio single molecule sequencing, but were not able to estimate the relative abundance of all transcripts due to a sequencing bias favoring shorter transcripts. For this reason, relative abundances could only be specified within message size classes (i.e. the 4 kb, 2 kb and unexpectedly a 1 kb size class as well) and the overall quantitative abundances were unknown. Our RNA-Seq data are unable to reconstruct the multiply spliced messages due to short read lengths but do permit estimation of size class abundances after correcting for 3′ bias (Additional file 6). Thus the PacBio data reported by Ocwieja et al. [6] and the Illumina data reported here can be combined together to determine complete relative abundance of 78 HIV_89.6_ transcripts (Fig. 6b).

The most abundant HIV mRNAs were the unspliced HIV genome (37.6 %), a transcript encoding Nef (D1-A5-D4-A7: 15.5 %), two 1 kb size class transcripts (D1-A5-D4-A8c: 10.6 %, D1-A8c: 4.9 %) and two Rev-encoding transcripts (D1-A4c-D4-A7: 4.2 %, D1-A4b-D4-A7: 3.1 %).

Using these abundances, we can estimate the number of HIV_89.6_ genomes in these primary T cells 48 h after infection. To determine the proportion of the mRNA nucleotides from viral transcripts, we multiplied the estimated abundances by their transcript lengths. Unspliced genome transcripts appear to form 79 % of the mRNA nucleotides from HIV_89.6_ transcripts. Assuming T cells contain at least 0.1 pg of mRNA then an infected cell should contain at least 0.011 pg of unspliced HIV transcript ($0.1\text {pg}\times 0.14\frac{\text {HIV mRNA nt}}{\text {cell mRNA nt}}\times 0.79\frac{\text {unspliced mRNA nt}}{\text {HIV mRNA nt}}$) or, assuming 9171 bases of RNA weigh about $5 \times 10^{-6}$ pg, at least 2200 HIV genomes at 48 h post infection. This estimate roughly agrees with previous estimates of HIV production per cell [92, 94, 95].

### Human-HIV chimeric reads

In our data, 80,045 reads contained sequences matching to both HIV and human genomic DNA. For a baseline measure of HIV_89.6_ integration patterns, we used ligation-mediated PCR to recover provirus-human junctions from the same infected cell populations, yielding 147,281 unique integration sites [96].

Comparison between these two datasets revealed abundant RNA-Seq chimeras formed between HIV and mitochondrial sequences while no proviral integrations into mitochondria were observed (Additional file 7A) or have been previously reported [53]. This likely indicates significant contamination with chimeras formed during the preparation of libraries for sequence analysis [97–104]. Potential mechanisms include template switching between sequences with shared similarity during reverse transcription [105–107] and priming off incomplete transcripts during DNA synthesis [97, 98, 108, 109]. To account for these artifacts, we retained only the 605 reads with no overlap and no unknown intervening sequence between human and HIV portions (Additional file 7B) where the HIV sequence bordered the 3′ or 5′ end of HIV or an HIV splice donor or acceptor (Additional file 7C).

**Fig. 7:**
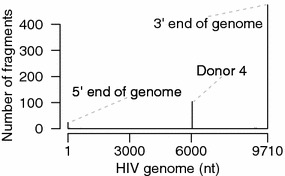
Analysis of chimeric RNA sequences containing both human and HIV sequences. Counts of the location in the HIV genome of the HIV-human junctions in filtered chimeric reads. Due to abundant sequencing artifacts (Additional file 7), reads were filtered to exclude reads where the human and HIV portions contained overlapping complementarity at the sequence junction (a sign of potential artifactual formation) and to exclude reads where the viral portion did not start at a known splice site or 5′ or 3′ border of the HIV genome

Chimeric messages composed of HIV and cellular RNA sequences can be formed by cellular gene transcription reading into the integrated provirus, by HIV transcription reading out through the viral polyadenylation site or by splicing between human and viral splice sites. In our filtered data, the predominant forms appear to be derived from reading through the HIV polyadenylation signal into the surrounding DNA (78 %), splicing out of the viral D4 splice donor to join to human slice acceptors (17 %) and reading into the HIV 5′ LTR from human sequence (4.0 %) (Fig. 7). No splice site other than D4 had more than two chimeric reads observed.

The filtered chimeric reads had many traits consistent with biological chimera formation. The reads containing HIV D4 joined to human sequences had the characteristics expected of splicing—72.1 % of the chimeric junctions mapped to known human acceptors and 96.1 % mapped to a location immediately preceded by the AG consensus of human mRNA acceptors. The reads containing the 5′ or 3′ LTR border were almost exclusively (93 %) found in transcription units, with odds of being in a gene 2.3-fold (95 % CI 1.6–3.2, $p=10^{-7}$) higher than integration sites from the same sample. The readthrough chimeras were also more likely to be located in an exon than integration sites (odds ratio: 2.1, 95 % CI 1.6–2.6, $p=10^{-7}$ only considering integration sites and chimeras in transcription units).

Chimeric sequences have the potential to alter the expression of proto-oncogenes leading to proliferation of the host cell [57–60]. We investigated possible effects of integration on cell proliferation by asking whether chimeric RNAs were more common at proto-oncogenes. HIV has been reported to integrate near oncogenes more often than expected by chance [110] and here integrations were more frequent in genes annotated as proto-oncogenes by the Uniprot Knowledgebase [111, 112] than in matched random controls [113] (odds ratio: 3.84, 95 % CI 3.72–3.97, $p=0.0005$). To account for this preference, we compared the locations of RNA-Seq chimeras to those of integration sites from the same samples. In these data, we saw no significant enrichment for chimeric mRNA to originate in transcription units annotated as proto-oncogenes relative to integration sites (Fisher’s exact test $p=0.15$). This lack of significant enrichment might be expected since cells were infected for only 48 h and there would be little time opportunity for selection to occur during cell growth.

We next compared whether the human and viral segments of chimeric reads agreed or disagreed in orientation (i.e. strand transcribed) for reads with the human portion mapped within annotated transcription units. The sequencing technique used here does not preserve strand information, but we can check whether the strand of a sequence read agrees or disagrees with the annotated gene strand and compare this to the observed strand of the HIV portion of the read. Chimeras involving HIV splice donor D4 were highly enriched for matching orientations (odds ratio: 52.5, 95 % CI 12.1–307, $p=10^{-11}$) suggesting that pairing with human splice acceptors constrains the orientation of D4 chimeric reads. We also found a strong association between the orientation of the human and HIV portions of chimeric reads within 3′ and 5′ chimeras (odds ratio: 6.2, 95 % CI 3.9–10.2, $p<10^{-15}$). This highly significant enrichment of HIV and human genes in the same orientation might indicate that antisense HIV RNA is rapidly degraded by a response to double-stranded RNA or that polymerases oriented in opposing directions interfere with one another during elongation.

Based on these data, we can propose a lower bound on the relative abundance of chimeras. If we assume that our filtering removed nearly all artifacts so that we have few false positives, then our estimate should be lower than the true proportion of chimeras. In our data, only $\frac{604}{12,689,879{}} = 0.0048\,\%$ of reads containing sequence mapping to HIV also contained identifiable chimeric junctions. However, this is an underestimate because in an HIV-derived mRNA, any fragment of the sequence will be mappable to HIV, while for a chimeric sequence only a read spanning the HIV-human junction will allow identification of a chimera. If we assume that 25 bases of sequence are necessary to map to human or HIV sequence, then, with the 100 bp reads used here, only read fragments starting between 75 and 25 bp downstream of the chimeric junction will be identifiable. If we assume the average chimeric mRNA sequences is at least 2 kb long, then a read from a chimeric sequence has at most a $\frac{50}{2000}=2.5\,\%$ chance of containing a mappable junction. Thus, a lower bound for the proportion of HIV mRNA that also contain human-derived sequences is 0.2 % ($\frac{0.0048\text \,{ \%}}{2.5\,\%}$). Looking only at splicing from HIV donor D4, we saw 16,843 reads containing a junction from D4 to an HIV acceptor and 104 reads from D4 to human sequence. Thus, in our data, 0.6 % of D4 splice products form junctions with human acceptors instead of HIV acceptors.

## Discussion

Here we used RNA-Seq to analyze mRNA accumulation and splicing in primary T cells infected with the low passage isolate HIV_89.6_. We did not carry out dense time series analysis, compare different human cell donors or compare different perturbations of the infections—instead, we focused on generating a dense data set at a single time point. We analyzed replicate infected cell and control samples to allow discrimination of within-condition versus between-condition variation and assessed differences using a series of bioinformatic approaches. Many previous studies have used microarray technology or RNA-Seq to study gene activity in HIV-infected cells [17–22, 24–28, 36], usually analyzing infections of transformed cell lines or laboratory-adapted strains of HIV-1. Here we present what is to our knowledge the deepest RNA-Seq data set reported for infection in primary T cells using a low passage HIV isolate.

This RNA-Seq data set was paired with a set of 147,281 unique integration site sequences extracted from the same infections, which were critical to our ability to quality control chimeric reads. An advantage of studies using cell lines and laboratory-adapted strains is that a high proportion of cells can be infected, whereas in this study we achieved only $\mathord {\sim }30\,\%$ infection. However, we report distinctive features of the transcriptional response not seen in studies of HIV infections in cell lines. Novel in this study are (1) identification of intron retention as a consequence of HIV infection, (2) the finding of activation of ERV-9/LTR12C after HIV infection, (3) generation of a quantitative account of the structures and abundances of over 70 HIV_89.6_ messages and (4) clarification of the predominant types of HIV-host transcriptional chimeras. These findings are discussed below.

Broad changes in host cell mRNA abundances were evident after infection, with over 17 % of expressed genes changing significantly in activity. Changes included response to viral infection, apoptosis and T cell activation. Although it is not possible here to separate the response of infected and bystander cells, this study highlights the drastic changes in cellular expression caused by HIV-1 infection. In a meta-analysis including four previously published studies, no gene was detected as differentially expressed in all five studies and only a handful of genes appeared in four out of five studies. Further analyses showed that expression changes appear to be cell type specific, raising concerns that studies using cell lines may not fully reflect host cell responses in in vivo infections.

Unexpectedly, intronic sequences were more common in the RNA-Seq data from cells after HIV_89.6_ infection than in mock infected cells. The mechanism is unclear. It is possible that the splicing machinery is reduced in activity after 48 h of infection, perhaps as a part of the antiviral response of infected and bystander cells. HIV infection does appear to alter expression and localization of some splicing factors [35, 114, 115] and genes involved in RNA splicing were more likely to be differentially expressed in our study (Benjamini–Hochberg corrected Fisher’s exact test $q=2\times 10^{-5}$). Alternatively, fully spliced mRNAs might be more rapidly degraded after infection, possibly by interferon-mediated induction of RNaseL [116, 117] or off-target binding of viral protein Rev might mediate export of incompletely spliced cellular transcripts [118, 119]. A speculative possibility is that HIV_89.6_ encodes a factor that alters cellular splicing or promotes mRNA degradation to optimize splicing and translation of viral messages.

Ribosomal protein genes were particularly affected by intron retention. Several of these genes have been reported to autoregulate protein abundance through a feedback loop where the protein represses splicing of its own mRNA transcripts to generate unproductive spliceforms [120–122]. HIV infection can cause a decrease in fully processed ribosomal RNA [29] likely through the interferon-activated RNaseL pathway [123, 124]. Here, we do not have a direct measure of rRNA abundance due to the poly-A selection but we did see an apparent decrease in total RNA yield in HIV-infected samples. Decreased rRNA might lead to more free ribosomal proteins which could suppress splicing of ribosomal gene transcripts during HIV infection. However, previous reports of alternative splicing in ribosomal protein genes have involved specific introns rather than the broad intron retention seen here perhaps indicating that both the intron retention and the general decrease of expression of ribosomal genes may be part of an innate immune response repressing translation [125, 126].

Infection resulted in increased expression of specific cellular repeated sequences. HERVs, in particular HERV-K, have previously been observed to show increased RNA accumulation with HIV infection [37–42, 47] and possibly represent vaccine targets because of their production of distinctive proteins [44–47, 83, 127]. Here, though we saw modest increases in HERV-K expression, ERV-9 had the greatest changes in expression (33 LTR12C and 14 ERV-9 annotated regions with greater than $4\times$ change in expression). Previous RNA-Seq studies of HIV infection in cell lines did not report increases in HERV expression [24, 25] but this difference is likely due to a much higher baseline expression of HERVs in transformed cell lines. We also observed increases in LINE and Alu element transcription, as has been reported previously [43], and expression changes in ERV-9/LTR12C expression associated with the density of transcription factor binding motifs within specific U3 variants.

Many of the repeated sequence elements that were induced by HIV_89.6_ infection are relatively recently integrated in the human genome. The reason for this pattern has been unclear. It may be that older elements have accumulated more mutations, resulting in an inactivation of transcriptional signals. Alternatively, perhaps the elements that are induced have been recruited for transcriptional control of cellular functions, so that their transcriptional activity is preserved evolutionarily [90, 91, 128–131].

Comparison of the results of sequencing HIV_89.6_ messages using long-read single molecule sequencing (Pacific Biosciences from Ocwieja et al. [6]) and dense short read sequencing (Illumina data reported here) allowed a full quantitative accounting of more than 70 HIV_89.6_ splice forms. The full length unspliced HIV RNA comprised 37.6 % of all messages, corresponding to more than 2000 genomes per cell. Notably abundant messages included the full length genome and spliced transcripts encoding Nef and Rev transcripts. The full set of messages is summarized in Fig. 6b.

Our previous analysis using PacBio sequencing [6] revealed an unusually prominent 1 kb size class. HIV_89.6_ encodes a splice acceptor (A8c) within Nef responsible for formation of the short messages. Our data indicated that two members of the 1-kb size class, D1-A5-D4-A8c and D1-A8c, accounted for 10.6 % and 4.9 % of all viral messages. The 1 kb size class as a whole accounted for fully 20 % of messages. The function of this large amount of 1 kb transcript is unknown. The most abundant 1 kb transcripts do not appear to encode significant open reading frames although other 1 kb transcripts can encode a Rev-Nef fusion [6]. Most HIV/SIV variants do appear to encode an acceptor near this position, suggesting a potential unknown function for these short spliced forms [6, 132, 133]. This analysis also suggests a lower proportion of 4 kb messages than has been seen for another isolate [134] suggesting that these ratios may vary with strain, time of infection or other conditions [6].

After filtering, we detected a sizeable number of apparently authentic chimeras containing both HIV and cellular sequences. Mechanisms of insertional activation have been studied intensively in animal models of transformation and in adverse events in human gene therapy. One of the most common mechanisms involves insertion of a retroviral enhancer near a cellular promoter, so that transcription of a nearby gene is increased [58, 135–137]. However, another common mechanism involves formation of chimeric messages involving both cellular and viral/vector sequences [57, 58]. A targeted in vitro study of chimeric message formation by lentiviral vectors showed examples of multiple types of splice-in messages [59], which may have been more frequent and more varied than for the HIV_89.6_ proviruses studied here. The low level of chimeric splicing into and reading into HIV in this study may be a reflection of the high rate of HIV transcription in these infected cells—because HIV was so highly expressed, there would be more opportunities for polymerase to splice out of or read through the HIV genome than to read or splice in. The vast majority of HIV proviruses in expanded clones in well-suppressed patients appear to be defective [51]—going forward, it will be of interest to investigate whether these HIV proviruses are damaged in ways that promote formation of chimeric transcripts.

Lastly, we note that several features of the transcriptional response to HIV_89.6_ infection were suggestive of de-differentiation away from T cell specific expression patterns. The increase in expression of cellular HERVs and LINEs is characteristic of cells in early development. Specific HERVs and transposons, including ERV-9/LTR12C and HERV-K, have been implicated in regulating gene activity early in development [90, 128, 131, 138–141]. Several genes related to other hematopoietic cell types showed elevated RNA abundance after HIV_89.6_ infection. These data are of interest given the finding that patients undergoing long term ART can contain long lived T cell clones that may contribute to the latent reservoir [51, 142–145]. Possibly the transcriptional responses seen here in infected primary T cells are reflective of processes leading to the formation of latently-infected cells with stem-like properties.

## Conclusions

Infections of primary T cells with a low passage HIV isolate showed several distinctive features compared to previously published data using T cell lines and/or lab-adapted HIV strains. We found strong changes in expression in genes related to immune response and apoptosis similar to studies of HIV infection in patient samples and primary cells but different from studies performed in SupT1 cell lines. Notable changes after infection included intron retention and activation of recently integrated retrotransposons and endogenous retroviruses, in particular ERV-9/LTR12C. We also present complete absolute estimation of over 70 messages from HIV_89.6_ and specify the major virus-host chimeras as splicing from viral splice donor 4 to cellular acceptors and readthrough from the 5′ and 3′ ends of the provirus.

## Methods

### Cell culture and viral infections

HIV_89.6_ stocks were generated by the University of Pennsylvania Center for AIDS Research. 293T cells were transfected with a plasmid encoding an HIV_89.6_ provirus, and harvested virus was passaged in SupT1 cells once. Viral stocks were quantified by measuring p24 antigen content. Primary CD4^+^ T cells were isolated by the University of Pennsylvania Center for AIDS Research Immunology Core from apheresis product from a single healthy male donor (ND365) using the RosetteSep Human CD4^+^ T Cell Enrichment Cocktail (StemCell Technologies). The Immunology Core maintains the IRB-approved protocol (IRB #705906) and receipt of these cells is considered secondary use of de-identified human specimens.

T cells were stimulated for 3 days at $0.5 \times 10^6$ cells per milliliter in R10 media (RPMI 1640 with GlutaMAX (Invitrogen) supplemented with 10 % FBS (Sigma-Aldrich) with 100 units U/mL recombinant IL2 (Novartis) + 5 µg/mL PHA-L (Sigma-Aldrich)). Here PHA and IL2 were used for their strong activating effects but further investigation using cells activated in a more physiological way might provide further benefits. Cells were infected in triplicate and mock infections were performed in duplicate. For each infection, $6.6 \times 10^6$ cells were mixed with 1.32 µg HIV_89.6_ in a total volume of 2.25 mL. Infection mixtures was split into three wells of a 6 well plate for spinoculation at 1200 g for 2 h at 37 °C. Cells were incubated an additional 2 hr at 37 °C. Cells were then pooled into flasks and volume was increased to a total of 12 mL. Spreading infection was allowed to proceed 48 hr at 37 °C, after which cells were harvested. $10^6$ cells were harvested for flow cytometry, and $6 \times 10^6$ cells were pelleted following two washes in PBS for nucleic acid extraction. Genomic DNA and total RNA were isolated from $6 \times 10^6$ T cells per infection using the AllPrep DNA/RNA Mini Kit (Qiagen) with Qiashredder columns (Qiagen) for homogenization according to the manufacturer’s instructions. DNA was eluted in 140 µL elution buffer. RNA samples were treated with DNase prior to elution in 40 µL water.

### Analysis of HIV_89.6_ integration sites in primary T cells

Integration site sequences were determined for DNA fractions from the above infections after ligation mediated PCR [96]. A total of 147,281 unique integration site sequences were determined. An analysis of integration site distributions for these samples was reported in Berry et al. [96].

### mRNA sequencing

Messenger RNA was isolated and amplified from purified total cellular RNA (3 µL or approximately 9 µg from each uninfected sample, 25 µL or approximately 3 µg from each infected sample) using the Illumina TruSeq RNA sample preparation kit according to manufacturer’s protocol. SuperScript III (Invitrogen) was used for reverse transcription. Each sample was tagged with a separate barcode and sequenced on an Illumina HiSeq 2000 using 100 bp paired-end chemistry.

### Flow cytometry

To assess percent infected cells, $10^6$ cells per infection were stained for flow cytometry. All staining incubations were at room temperature. Cells were first washed in PBS and then twice in FACS wash buffer (PBS, 2.5 % FBS, 2 mM EDTA). Cells were fixed and permeabilized with CytoFix/CytoPerm (BD) for 20 minutes and washed with Perm-Wash Buffer (BD) before staining with anti-HIV-Gag-PE (Beckman Coulter) for 60 min. Finally cells were washed in FACS wash buffer and resuspended in 3 % PFA. Samples were run on a LSRII (BD) and analyzed with FlowJo 8.8.6 (Treestar). Cells were gated as follows: lymphocytes (SSC-A by FSC-A), then singlets (FSC-A by FSC-H), then by Gag expression (FSC-A by Gag).

### Analysis

Reads were aligned to the human genome using a combination of BLAT [146] and Bowtie [147] through the Rum pipeline [148]. Estimates of fragments per kilobase of transcript per million mapped reads and changes in expression for cellular genes were calculated by Cufflinks [149]. Reads found to contain sequence similar to the HIV genome using a suffix tree algorithm were aligned against the HIV_89.6_ genome using BLAT [146]. All statistical analyses were performed in R 3.1.2 [150]. RNA-Seq reads from Chang et al. [25] were downloaded from the Sequence Read Archive (SRP013224) and aligned using the Rum pipeline.

Gene lists were obtained from the supplementary materials of four other studies of differential gene expression during HIV infection [24–26, 30]. We called genes differentially expressed in Li et al. [30] if they had a reported $p<0.01$ or in Lefebvre et al. [24], Chang et al. [25] and Imbeault et al. [26] if they had an adjusted $p<0.05$. We called genes as differentially expressed in our own study if the adjusted $p<0.01$. For the comparison of differentially expressed genes regardless of direction in Fig. 1 (below the diagonal), it was unclear exactly how many genes were studied in each study so we assumed a background of the 14,192 genes (the number of genes that could be tested for significance in our data).

We obtained transcriptional profiles comparing immune cell subsets from the Molecular Signatures Database [68]. MSigDB set names from the MSigDB used in Fig. 2a were GSE10325 LUPUS CD4 TCELL VS LUPUS BCELL, GSE10325 CD4 TCELL VS MYELOID, GSE10325 CD4 TCELL VS BCELL, GSE10325 LUPUS CD4 TCELL VS LUPUS MYELOID, GSE3982 MEMORY CD4 TCELL VS TH1, GSE22886 CD4 TCELL VS BCELL NAIVE, GSE11057 CD4 CENT MEM VS PBMC, GSE11057 CD4 EFF MEM VS PBMC, GSE3982 MEMORY CD4 TCELL VS TH2 and GSE11057 PBMC VS MEM CD4 TCELL and in Fig. 2b were GSE36476 CTRL VS TSST ACT 72H MEMORY CD4 TCELL OLD, GSE10325 CD4 TCELL VS LUPUS CD4 TCELL, GSE22886 NAIVE CD4 TCELL VS 12H ACT TH1, GSE3982 CENT MEMORY CD4 TCELL VS TH1, GSE17974 CTRL VS ACT IL4 AND ANTI IL12 48H CD4 TCELL, GSE24634 IL4 VS CTRL TREATED NAIVE CD4 TCELL DAY5, GSE24634 NAIVE CD4 TCELL VS DAY10 IL4 CONV TREG, GSE1460 CD4 THYMOCYTE VS THYMIC STROMAL CELL and GSE1460 INTRATHYMIC T PROGENITOR VS NAIVE CD4 TCELL ADULT BLOOD.

We downloaded the RepeatMasker [76] track from the UCSC genome browser [151] and used the SAMtools library [152] to assign reads to the repeat regions. HERV-K age estimates were obtained from the supplementary materials of Subramanian et al. [82].

We used a Bayesian estimate of the ratio of expression in uninfected and HIV-infected samples to account for sampling effort and differing expression in genomic regions. We modeled the observed counts as a binomial distribution with a flat beta prior ($\alpha =1,\beta =1$) separately for uninfected and infected samples. We then Monte Carlo sampled the two posterior distribution to estimate the posterior distribution of the ratio. For introns, the number of binomial successes was set to the number of reads mapped to the intron and the number of trials was the total number of reads observed in the genes overlapping that intron. For repeat regions, the number of binomial successes was set to the number of reads mapped to that region and the number of trials was the total number of reads mapped to the human genome.

Lasso regression was performed using the R package glmnet [153]. The $\lambda$ smoothing parameter of the lasso regression was optimized by finding the $\lambda$ with lowest mean squared error in a 500-fold cross validation and picking the simplest model with misclassification error within one standard error.

To estimate determinants of ERV-9/LTR12C expression, we fit a logistic regression for which LTR12C increased in expression with HIV_89.6_ infection (95 % Bayesian credible interval $>2\times$ change) on to characteristics of the LTR12C regions. We extracted all the LTR12C regions from the human genome and determined the U3-R boundary using a ends free alignment of the previously reported U3-R border [88–90, 154, 155] against the sequences. Regions less than 1,000 bases long were discarded. Previous studies disagreed about the location of the LTR12C transcription start site and it appears that transcription may start in several places [88, 155]. We took the 5′ most site that had agreement between studies (transcription starting with TGGCAACCC). We split the sequences into short and longer length classes based on repeated sequences about 70 bases upstream from the transcription start site. For the short and 3 subtypes within the long length class, we generated a consensus sequence and counted the Levenshtein edit distance between the consensuses and each corresponding sequence. We also counted the number of NFY motifs (CCAAT or ATTGG), MZF1 motifs (GTGGGGA) and GATA2 motifs (GATA or TATC) in the entire U3 region and checked if a TATA box (AATAAA) [90] was present in the 50 bases upstream of the TSS. A final regression model was selected using stepwise regression with an AIC cutoff of 5. For display, the LTR12C sequences were aligned with MUSCLE [156].

The abundance of the HIV RNA size classes was estimated as described in Additional file 6. These estimates were then multiplied by the within size class proportions estimated by Ocwieja et al. [6] using PacBio sequencing of HIV_89.6_ to yield proportions over 78 measured HIV_89.6_ RNAs.

## Availability of supporting data

RNA-Seq reads from this study are available at the Sequence Read Archive under accession number SRP055981. The integration site data is available at the Sequence Read Archive under accession number SRP057555.

## Additional files

RNA-Seq reads from this study are available at the Sequence Read Archive under accession number SRP055981. The integration site data is available at the Sequence Read Archive under accession number SRP057555.

10.1186/s12977-015-0205-1 **Analysis of genes differentially expressed during HIV**_**89.6**_ **infection of primary CD4** ^**+**^ **T cells.** Output from CuffLinks analysis of the RNA-Seq data is organized in a csv file with columns UCSC gene ID(s), gene symbol(s), status of test, FPKM in uninfected and infected samples, the $\log _2$ fold change, test statistic and false discovery rate adjusted *p*-value.
10.1186/s12977-015-0205-1 **Analysis of Gene Ontology categories associated with differential expression during HIV**_**89.6**_ **infection of primary CD4** ^**+**^ **T cells**. Counts of differentially expressed genes for each Gene Ontology category are stored in a csv file with columns category name, the numbers of genes differentially up- and downregulated or not significantly changed and odds ratios and *p*-values from Fisher’s exact tests.
10.1186/s12977-015-0205-1 **Studies used in a meta-analysis of differential expression during HIV infection.** Details of studies used in the meta-analysis of differential expression in HIV-infected cells are stored in a csv file with columns showing the alias used in figures, cell type and HIV used in the study, genes differentially expressed in either direction, gene upregulated, genes downregulated and the PubMed ID of the study.
10.1186/s12977-015-0205-1 **Genes called as up- or downregulated by studies of expression during HIV infection.** Genes called as differentially expressed in the five studies analyzed in the meta-analysis of differential expression with HIV infection are stored in a csv file with columns study, the gene name(s) and whether the differential expression was up or down.
10.1186/s12977-015-0205-1 **Expression observed in the top 50 most differentially expressed introns in ribosomal protein genes and ERV-9/LTR12C regions with expression continuing for greater than 1000bp downstream.** A) RNA-Seq read counts for the 50 most differentially expressed in introns in ribosomal protein genes. The number of reads covering each base is shown on the y-axis. UCSC genes and RepeatMasker LTRs are annotated below with lines indicating introns and boxes indicating exons or repeats. Dashed vertical lines highlight the region detected as differentially expressed. B) As in A but showing RNA-Seq read counts for ERV-9/LTR12C with expression continuing for greater than 1000 bp 3′ of the LTR region. LTRs were selected by filtering for regions with a 90th percentile coverage higher than 30 and looking for the furthest 3′ base with continuous (no gap greater than 200 bp) coverage of at least 10 % of the 90th percentile of expression within the LTR.
10.1186/s12977-015-0205-1 **Estimating relative abundance of HIV**_**89.6**_ **message size classes using RNA-Seq data.** A) RNA-Seq coverage of the HIV_89.6_ genome for the replicates in this study. Each replicate is indicated by a different color. The HIV genome is shown on the x-axis and the number of reads that aligned to each position is shown on the y-axis. Black line indicates the 0.021 % coverage decrease per base distance from the 3′ end of the mRNA estimated from a least squares fit on the read counts in the first intron. B) Diagram of the segments of the HIV_89.6_ RNA present in each of 9 kb, 4 kb, 2 kb and 1 kb size class. C) The proportion of reads mapped to each of the segments of the HIV_89.6_ genome shown in B adjusted by the length of the segment. Each replicate is shown by a different color. D) Corrected representation of RNA segments from the different size classes. Because cDNA synthesis was primed from the polyA tail, more 3′ sequences are recovered preferentially. Using the bias estimate from A, we adjusted each genome segment by the inverse of the bias predicted based on its distance from the 3′ end of the mRNA. Corrected proportions for the indicated RNA segments are shown colored by replicate. The first exon can not be adjusted using this method since multiple lengths of messages will include it. E) The proportion of each size class was inferred using the estimates in D by calculating the difference between segments. Replicates are indicated by color.
10.1186/s12977-015-0205-1 **Comparison with integration site data reveals an abundance of artifactual chimeras.** A) Chromosomal distribution of uniquely mapping HIV integration sites from the same infections of primary T cells and comparison to uniquely mapping human sequences in chimeric reads observed in RNA-Seq. Note that the mitochondrial genome, denoted as M, has no authentic integration sites but does have extensive matches to chimeric junctions found in the RNA-Seq data. B) The length of overlapping sequence (regions of complementarity potentially favoring chimera formation) matching both human and HIV at inferred chimeric junctions. The x-axis shows the length of the overlap and the y-axis shows the frequency of chimeric junctions with the indicated extent of overlap. C) The effects of filtering for chimeras with 1) an exact junction between human and HIV sequences with no unknown or overlapping sequence, 2) HIV segments starting at the 5′ or 3′ end of HIV or at known HIV splice sites or 3) both filters combined. A marked reduction in mitochondrial chimeras (inferred to be markers of sequencing artifacts) is observed with each filtering step.

## References

[1] Wain-Hobson S, Sonigo P, Danos O, Cole S, Alizon M (1985). Nucleotide sequence of the AIDS virus, LAV. Cell..

[2] Arya SK, Guo C, Josephs SF, Wong-Staal F (1985). Trans-activator gene of human T-lymphotropic virus type III (HTLV-III). Science..

[3] Schwartz S, Felber BK, Benko DM, Fenyö EM, Pavlakis GN (1990). Cloning and functional analysis of multiply spliced mRNA species of human immunodeficiency virus type 1. J Virol..

[4] Purcell DF, Martin MA (1993). Alternative splicing of human immunodeficiency virus type 1 mRNA modulates viral protein expression, replication, and infectivity. J Virol.

[5] Stoltzfus CM. Chapter 1. Regulation of HIV-1 alternative RNA splicing and its role in virus replication. Adv Virus Res. 2009;74:1–40. doi:10.1016/S0065-3527(09)74001-1. 10.1016/S0065-3527(09)74001-1 19698894

[6] Ocwieja KE, Sherrill-Mix S, Mukherjee R, Custers-Allen R, David P, Brown M, Wang S, Link DR, Olson J, Travers K, Schadt E, Bushman FD (2012). Dynamic regulation of HIV-1 mRNA populations analyzed by single-molecule enrichment and long-read sequencing. Nucleic Acids Res.

[7] He J, Choe S, Walker R, Di Marzio P, Morgan DO, Landau NR (1995). Human immunodeficiency virus type 1 viral protein R (Vpr) arrests cells in the G2 phase of the cell cycle by inhibiting p34cdc2 activity. J Virol.

[8] Jowett JB, Planelles V, Poon B, Shah NP, Chen ML, Chen IS (1995). The human immunodeficiency virus type 1 vpr gene arrests infected T cells in the G2 + M phase of the cell cycle. J Virol.

[9] Rogel ME, Wu LI, Emerman M (1995). The human immunodeficiency virus type 1 vpr gene prevents cell proliferation during chronic infection. J Virol.

[10] Goh WC, Rogel ME, Kinsey CM, Michael SF, Fultz PN, Nowak MA, Hahn BH, Emerman M (1998). HIV-1 Vpr increases viral expression by manipulation of the cell cycle: a mechanism for selection of Vpr in vivo. Nat Med.

[11] Marciniak RA, Sharp PA (1991). HIV-1 Tat protein promotes formation of more-processive elongation complexes. EMBO J.

[12] Wei P, Garber ME, Fang SM, Fischer WH, Jones KA (1998). A novel CDK9-associated C-type cyclin interacts directly with HIV-1 Tat and mediates its high-affinity, loop-specific binding to TAR RNA. Cell.

[13] Kanazawa S, Okamoto T, Peterlin BM (2000). Tat competes with CIITA for the binding to P-TEFb and blocks the expression of MHC class II genes in HIV infection. Immunity.

[14] Barboric M, Yik JHN, Czudnochowski N, Yang Z, Chen R, Contreras X, Geyer M (2007). Matija Peterlin, B., Zhou, Q.: Tat competes with HEXIM1 to increase the active pool of P-TEFb for HIV-1 transcription. Nucleic Acids Res.

[15] O’Brien SK, Cao H, Nathans R, Ali A, Rana TM (2010). P-TEFb kinase complex phosphorylates histone H1 to regulate expression of cellular and HIV-1 genes. J Biol Chem.

[16] Muniz L, Egloff S, Ughy B, Jády BE, Kiss T (2010). Controlling cellular P-TEFb activity by the HIV-1 transcriptional transactivator Tat. PLoS Pathog.

[17] Mitchell R, Chiang C-Y, Berry C, Bushman F (2003). Global analysis of cellular transcription following infection with an HIV-based vector. Mol Ther.

[18] Corbeil J, Sheeter D, Genini D, Rought S, Leoni L, Du P, Ferguson M, Masys DR, Welsh JB, Fink JL, Sasik R, Huang D, Drenkow J, Richman DD, Gingeras T (2001). Temporal gene regulation during HIV-1 infection of human CD4+ T cells. Genome Res.

[19] Woelk CH, Ottones F, Plotkin CR, Du P, Royer CD, Rought SE, Lozach J, Sasik R, Kornbluth RS, Richman DD, Corbeil J (2004). Interferon gene expression following HIV type 1 infection of monocyte-derived macrophages. AIDS Res Hum Retroviruses.

[20] Hyrcza MD, Kovacs C, Loutfy M, Halpenny R, Heisler L, Yang S, Wilkins O, Ostrowski M, Der SD (2007). Distinct transcriptional profiles in ex vivo CD4+ and CD8+ T cells are established early in human immunodeficiency virus type 1 infection and are characterized by a chronic interferon response as well as extensive transcriptional changes in CD8+ T cells. J Virol.

[21] Wu JQ, Dwyer DE, Dyer WB, Yang YH, Wang B, Saksena NK (2008). Transcriptional profiles in CD8+ T cells from HIV+ progressors on HAART are characterized by coordinated up-regulation of oxidative phosphorylation enzymes and interferon responses. Virology.

[22] Smith AJ, Li Q, Wietgrefe SW, Schacker TW, Reilly CS, Haase AT (2010). Host genes associated with HIV-1 replication in lymphatic tissue. J Immunol.

[23] Rotger M, Dang KK, Fellay J, Heinzen EL, Feng S, Descombes P, Shianna KV, Ge D, Günthard HF, Goldstein DB, Telenti A; SHC Study; Center for HIV/AIDS Vaccine Immunology. Genome-wide mRNA expression correlates of viral control in CD4+ T-cells from HIV-1-infected individuals. PLoS Pathog. 2010;6(2):1000781. doi:10.1371/journal.ppat.1000781. 10.1371/journal.ppat.1000781 PMC2829051 20195503

[24] Lefebvre G, Desfarges S, Uyttebroeck F, Muñoz M, Beerenwinkel N, Rougemont J, Telenti A, Ciuffi A (2011). Analysis of HIV-1 expression level and sense of transcription by high-throughput sequencing of the infected cell. J Virol.

[25] Chang ST, Sova P, Peng X, Weiss J, Law GL, Palermo RE, Katze MG. Next-generation sequencing reveals HIV-1-mediated suppression of T cell activation and RNA processing and regulation of noncoding RNA expression in a CD4+ T cell line. MBio. 2011;2(5). doi:10.1128/mBio.00134-11. 10.1128/mBio.00134-11 PMC3175625 21933919

[26] Imbeault M, Giguère K, Ouellet M, Tremblay MJ (2012). Exon level transcriptomic profiling of HIV-1-infected CD4(+) T cells reveals virus-induced genes and host environment favorable for viral replication. PLoS Pathog.

[27] Mohammadi P, Desfarges S, Bartha I, Joos B, Zangger N, Muñoz M, Günthard HF, Beerenwinkel N, Telenti A, Ciuffi A (2013). 24 hours in the life of HIV-1 in a T cell line. PLoS Pathog.

[28] Peng X, Sova P, Green RR, Thomas MJ, Korth MJ, Proll S, Xu J, Cheng Y, Yi K, Chen L, Peng Z, Wang J, Palermo RE, Katze MG (2014). Deep sequencing of HIV-infected cells: insights into nascent transcription and host-directed therapy. J Virol.

[29] Kleinman CL, Doria M, Orecchini E, Giuliani E, Galardi S, De Jay N, Michienzi A (2014). HIV-1 infection causes a down-regulation of genes involved in ribosome biogenesis. PLoS One.

[30] Li Q, Smith AJ, Schacker TW, Carlis JV, Duan L, Reilly CS, Haase AT (2009). Microarray analysis of lymphatic tissue reveals stage-specific, gene expression signatures in HIV-1 infection. J Immunol.

[31] Rotger M, Dalmau J, Rauch A, McLaren P, Bosinger SE, Martinez R, Sandler NG, Roque A, Liebner J, Battegay M, Bernasconi E, Descombes P, Erkizia I, Fellay J, Hirschel B, Miró JM, Palou E, Hoffmann M, Massanella M, Blanco J, Woods M, Günthard HF, de Bakker P, Douek DC, Silvestri G, Martinez-Picado J, Telenti A (2011). Comparative transcriptomics of extreme phenotypes of human HIV-1 infection and SIV infection in sooty mangabey and rhesus macaque. J Clin Invest.

[32] Gao D, Wu J, Wu Y-T, Du F, Aroh C, Yan N, Sun L, Chen ZJ (2013). Cyclic GMP-AMP synthase is an innate immune sensor of HIV and other retroviruses. Science.

[33] Rasaiyaah J, Tan CP, Fletcher AJ, Price AJ, Blondeau C, Hilditch L, Jacques DA, Selwood DL, James LC, Noursadeghi M, Towers GJ (2013). HIV-1 evades innate immune recognition through specific cofactor recruitment. Nature.

[34] Monroe KM, Yang Z, Johnson JR, Geng X, Doitsh G, Krogan NJ, Greene WC (2014). IFI16 DNA sensor is required for death of lymphoid CD4 T cells abortively infected with HIV. Science.

[35] Dowling D, Nasr-Esfahani S, Tan CH, O’Brien K, Howard JL, Jans DA, Purcell DF, Stoltzfus CM, Sonza S. HIV-1 infection induces changes in expression of cellular splicing factors that regulate alternative viral splicing and virus production in macrophages. Retrovirology. 2008;5:18. doi:10.1186/1742-4690-5-18. 10.1186/1742-4690-5-18 PMC2267807 18241354

[36] de la Fuente C, Santiago F, Deng L, Eadie C, Zilberman I, Kehn K, Maddukuri A, Baylor S, Wu K, Lee CG, Pumfery A, Kashanchi F (2002). Gene expression profile of HIV-1 Tat expressing cells: a close interplay between proliferative and differentiation signals. BMC Biochem.

[37] Vincendeau M, Göttesdorfer I, Schreml JMH, Wetie AGN, Mayer J, Greenwood AD, Helfer M, Kramer S, Seifarth W, Hadian K, Brack-Werner R, Leib-Mösch C (2015). Modulation of human endogenous retrovirus (HERV) transcription during persistent and de novo HIV-1 infection. Retrovirology.

[38] Contreras-Galindo R, Kaplan MH, Markovitz DM, Lorenzo E, Yamamura Y (2006). Detection of HERV-K(HML-2) viral RNA in plasma of HIV type 1-infected individuals. AIDS Res Hum Retroviruses.

[39] Laderoute MP, Giulivi A, Larocque L, Bellfoy D, Hou Y, Wu H-X, Fowke K, Wu J, Diaz-Mitoma F (2007). The replicative activity of human endogenous retrovirus K102 (HERV-K102) with HIV viremia. AIDS.

[40] Contreras-Galindo R, López P, Vélez R, Yamamura Y (2007). HIV-1 infection increases the expression of human endogenous retroviruses type K (HERV-K) in vitro. AIDS Res Hum Retroviruses.

[41] Contreras-Galindo R, Kaplan MH, He S, Contreras-Galindo AC, Gonzalez-Hernandez MJ, Kappes F, Dube D, Chan SM, Robinson D, Meng F, Dai M, Gitlin SD, Chinnaiyan AM, Omenn GS, Markovitz DM (2013). HIV infection reveals widespread expansion of novel centromeric human endogenous retroviruses. Genome Res.

[42] Bhardwaj N, Maldarelli F, Mellors J, Coffin JM (2014). HIV-1 infection leads to increased transcription of human endogenous retrovirus HERV-K (HML-2) proviruses in vivo but not to increased virion production. J Virol.

[43] Jones RB, Song H, Xu Y, Garrison KE, Buzdin AA, Anwar N, Hunter DV, Mujib S, Mihajlovic V, Martin E, Lee E, Kuciak M, Raposo RAS, Bozorgzad A, Meiklejohn DA, Ndhlovu LC, Nixon DF, Ostrowski MA (2013). LINE-1 retrotransposable element DNA accumulates in HIV-1-infected cells. J Virol.

[44] Garrison KE, Jones RB, Meiklejohn DA, Anwar N, Ndhlovu LC, Chapman JM, Erickson AL, Agrawal A, Spotts G, Hecht FM, Rakoff-Nahoum S, Lenz J, Ostrowski MA, Nixon DF (2007). T cell responses to human endogenous retroviruses in HIV-1 infection. PLoS Pathog.

[45] Tandon R, SenGupta D, Ndhlovu LC, Vieira RGS, Jones RB, York VA, Vieira VA, Sharp ER, Wiznia AA, Ostrowski MA, Rosenberg MG, Nixon DF (2011). Identification of human endogenous retrovirus-specific T cell responses in vertically HIV-1-infected subjects. J Virol.

[46] SenGupta D, Tandon R, Vieira RGS, Ndhlovu LC, Lown-Hecht R, Ormsby CE, Loh L, Jones RB, Garrison KE, Martin JN, York VA, Spotts G, Reyes-Terán G, Ostrowski MA, Hecht FM, Deeks SG, Nixon DF (2011). Strong human endogenous retrovirus-specific T cell responses are associated with control of HIV-1 in chronic infection. J Virol.

[47] Jones RB, Garrison KE, Mujib S, Mihajlovic V, Aidarus N, Hunter DV, Martin E, John VM, Zhan W, Faruk NF, Gyenes G, Sheppard NC, Priumboom-Brees IM, Goodwin DA, Chen L, Rieger M, Muscat-King S, Loudon PT, Stanley C, Holditch SJ, Wong JC, Clayton K, Duan E, Song H, Xu Y, SenGupta D, Tandon R, Sacha JB, Brockman MA, Benko E, Kovacs C, Nixon DF, Ostrowski MA (2012). HERV-K-specific T cells eliminate diverse HIV-1/2 and SIV primary isolates. J Clin Invest.

[48] Ikeda T, Shibata J, Yoshimura K, Koito A, Matsushita S (2007). Recurrent HIV-1 integration at the BACH2 locus in resting CD4+ T cell populations during effective highly active antiretroviral therapy. J Infect Dis.

[49] Wagner TA, McLaughlin S, Garg K, Cheung CYK, Larsen BB, Styrchak S, Huang HC, Edlefsen PT, Mullins JI, Frenkel LM (2014). Proliferation of cells with HIV integrated into cancer genes contributes to persistent infection. Science.

[50] Maldarelli F, Wu X, Su L, Simonetti FR, Shao W, Hill S, Spindler J, Ferris AL, Mellors JW, Kearney MF, Coffin JM, Hughes SH (2014). Specific HIV integration sites are linked to clonal expansion and persistence of infected cells. Science.

[51] Cohn LB, Silva IT, Oliveira TY, Rosales RA, Parrish EH, Learn GH, Hahn BH, Czartoski JL, McElrath MJ, Lehmann C, Klein F, Caskey M, Walker BD, Siliciano JD, Siliciano RF, Jankovic M, Nussenzweig MC (2015). HIV-1 integration landscape during latent and active infection. Cell.

[52] Schröder ARW, Shinn P, Chen H, Berry C, Ecker JR, Bushman F (2002). HIV-1 integration in the human genome favors active genes and local hotspots. Cell.

[53] Wang C, Mitsuya Y, Gharizadeh B, Ronaghi M, Shafer RW (2007). Characterization of mutation spectra with ultra-deep pyrosequencing: application to HIV-1 drug resistance. Genome Res.

[54] Brady T, Lee YN, Ronen K, Malani N, Berry CC, Bieniasz PD, Bushman FD (2009). Integration target site selection by a resurrected human endogenous retrovirus. Genes Dev.

[55] Sherrill-Mix S, Lewinski MK, Famiglietti M, Bosque A, Malani N, Ocwieja KE, Berry CC, Looney D, Shan L, Agosto LM, Pace MJ, Siliciano RF, O’Doherty U, Guatelli J, Planelles V, Bushman FD (2013). HIV latency and integration site placement in five cell-based models. Retrovirology.

[56] Marini B, Kertesz-Farkas A, Ali H, Lucic B, Lisek K, Manganaro L, Pongor S, Luzzati R, Recchia A, Mavilio F, Giacca M, Lusic M (2015). Nuclear architecture dictates HIV-1 integration site selection. Nature.

[57] Cavazzana-Calvo M, Payen E, Negre O, Wang G, Hehir K, Fusil F, Down J, Denaro M, Brady T, Westerman K, Cavallesco R, Gillet-Legrand B, Caccavelli L, Sgarra R, Maouche-Chrétien L, Bernaudin F, Girot R, Dorazio R, Mulder G-J, Polack A, Bank A, Soulier J, Larghero J, Kabbara N, Dalle B, Gourmel B, Socie G, Chrétien S, Cartier N, Aubourg P, Fischer A, Cornetta K, Galacteros F, Beuzard Y, Gluckman E, Bushman F, Hacein-Bey-Abina S, Leboulch P (2010). Transfusion independence and HMGA2 activation after gene therapy of human *β*-thalassaemia. Nature.

[58] Hacein-Bey-Abina S, Garrigue A, Wang GP, Soulier J, Lim A, Morillon E, Clappier E, Caccavelli L, Delabesse E, Beldjord K, Asnafi V, MacIntyre E, Dal Cortivo L, Radford I, Brousse N, Sigaux F, Moshous D, Hauer J, Borkhardt A, Belohradsky BH, Wintergerst U, Velez MC, Leiva L, Sorensen R, Wulffraat N, Blanche S, Bushman FD, Fischer A, Cavazzana-Calvo M (2008). Insertional oncogenesis in 4 patients after retrovirus-mediated gene therapy of SCID-X1. J Clin Invest.

[59] Moiani A, Paleari Y, Sartori D, Mezzadra R, Miccio A, Cattoglio C, Cocchiarella F, Lidonnici MR, Ferrari G, Mavilio F (2012). Lentiviral vector integration in the human genome induces alternative splicing and generates aberrant transcripts. J Clin Invest.

[60] Cesana D, Sgualdino J, Rudilosso L, Merella S, Naldini L, Montini E (2012). Whole transcriptome characterization of aberrant splicing events induced by lentiviral vector integrations. J Clin Invest.

[61] Collman R, Balliet JW, Gregory SA, Friedman H, Kolson DL, Nathanson N, Srinivasan A (1992). An infectious molecular clone of an unusual macrophage-tropic and highly cytopathic strain of human immunodeficiency virus type 1. J Virol.

[62] Breuer K, Foroushani AK, Laird MR, Chen C, Sribnaia A, Lo R, Winsor GL, Hancock REW, Brinkman FSL, Lynn DJ. InnateDB: systems biology of innate immunity and beyond—recent updates and continuing curation. Nucleic Acids Res. 2013;41(Database issue):1228–1233. doi:10.1093/nar/gks1147. 10.1093/nar/gks1147 PMC3531080 23180781

[63] Rusinova I, Forster S, Yu S, Kannan A, Masse M, Cumming H, Chapman R, Hertzog PJ. Interferome v2.0: an updated database of annotated interferon-regulated genes. Nucleic Acids Res. 2013;41(Database issue):1040–1046. doi:10.1093/nar/gks1215. 10.1093/nar/gks1215 PMC3531205 23203888

[64] Ashburner M, Ball CA, Blake JA, Botstein D, Butler H, Cherry JM, Davis AP, Dolinski K, Dwight SS, Eppig JT, Harris MA, Hill DP, Issel-Tarver L, Kasarskis A, Lewis S, Matese JC, Richardson JE, Ringwald M, Rubin GM, Sherlock G (2000). Gene ontology: tool for the unification of biology. The Gene Ontology Consortium. Nat Genet.

[65] Chang ST, Thomas MJ, Sova P, Green RR, Palermo RE, Katze MG. Next-generation sequencing of small RNAs from HIV-infected cells identifies phased microrna expression patterns and candidate novel microRNAs differentially expressed upon infection. MBio. 2013;4(1):00549–00512. doi:10.1128/mBio.2000549-12. 10.1128/mBio.00549-12 PMC3560529 23386435

[66] Atak Kalender Z, De Keersmaecker K, Gianfelici V, Geerdens E, Vandepoel R, Pauwels D, Porcu M, Lahortiga I, Brys V, Dirks WG, Quentmeier H, Cloos J, Cuppens H, Uyttebroeck A, Vandenberghe P, Cools J, Aerts S (2012). High accuracy mutation detection in leukemia on a selected panel of cancer genes. PLoS One.

[67] Patel ES, Chang L-J (2012). Synergistic effects of interleukin-7 and pre-T cell receptor signaling in human T cell development. J Biol Chem.

[68] Subramanian A, Tamayo P, Mootha VK, Mukherjee S, Ebert BL, Gillette MA, Paulovich A, Pomeroy SL, Golub TR, Lander ES, Mesirov JP (2005). Gene set enrichment analysis: a knowledge-based approach for interpreting genome-wide expression profiles. Proc Natl Acad Sci USA.

[69] Imbeault M, Ouellet M, Tremblay MJ (2009). Microarray study reveals that HIV-1 induces rapid type-I interferon-dependent p53 mRNA up-regulation in human primary CD4+ T cells. Retrovirology.

[70] Iwase S, Furukawa Y, Kikuchi J, Nagai M, Terui Y, Nakamura M, Yamada H (1997). Modulation of E2F activity is linked to interferon-induced growth suppression of hematopoietic cells. J Biol Chem.

[71] Johnstone RW, Kerry JA, Trapani JA (1998). The human interferon-inducible protein, IFI 16, is a repressor of transcription. J Biol Chem.

[72] Williams BR (1999). PKR; a sentinel kinase for cellular stress. Oncogene.

[73] Ramana CV, Grammatikakis N, Chernov M, Nguyen H, Goh KC, Williams BR, Stark GR (2000). Regulation of c-myc expression by IFN-gamma through Stat1-dependent and -independent pathways. EMBO J.

[74] Liang S-L, Quirk D, Zhou A (2006). RNase L: its biological roles and regulation. IUBMB Life.

[75] Hsu F, Kent WJ, Clawson H, Kuhn RM, Diekhans M, Haussler D (2006). The UCSC known genes. Bioinformatics.

[76] Jurka J, Kapitonov VV, Pavlicek A, Klonowski P, Kohany O, Walichiewicz J (2005). Repbase update, a database of eukaryotic repetitive elements. Cytogenet Genome Res.

[77] Braunschweig U, Barbosa-Morais NL, Pan Q, Nachman EN, Alipanahi B, Gonatopoulos-Pournatzis T, Frey B, Irimia M, Blencowe BJ (2014). Widespread intron retention in mammals functionally tunes transcriptomes. Genome Res.

[78] Tibshirani R (1996). Regression shrinkage and selection via the lasso. J R Statist Soc B.

[79] Yeo G, Burge CB (2004). Maximum entropy modeling of short sequence motifs with applications to RNA splicing signals. J Comput Biol.

[80] Medstrand P, Mager DL (1998). Human-specific integrations of the HERV-K endogenous retrovirus family. J Virol.

[81] Macfarlane C, Simmonds P (2004). Allelic variation of HERV-K(HML-2) endogenous retroviral elements in human populations. J Mol Evol.

[82] Subramanian RP, Wildschutte JH, Russo C, Coffin JM (2011). Identification, characterization, and comparative genomic distribution of the HERV-K (HML-2) group of human endogenous retroviruses. Retrovirology.

[83] Büscher K, Trefzer U, Hofmann M, Sterry W, Kurth R, Denner J (2005). Expression of human endogenous retrovirus K in melanomas and melanoma cell lines. Cancer Res.

[84] Howard G, Eiges R, Gaudet F, Jaenisch R, Eden A (2008). Activation and transposition of endogenous retroviral elements in hypomethylation induced tumors in mice. Oncogene.

[85] Iskow RC, McCabe MT, Mills RE, Torene S, Pittard WS, Neuwald AF, Van Meir EG, Vertino PM, Devine SE (2010). Natural mutagenesis of human genomes by endogenous retrotransposons. Cell.

[86] Lee E, Iskow R, Yang L, Gokcumen O, Haseley P, Luquette LJ 3rd, Lohr JG, Harris CC, Ding L, Wilson RK, Wheeler DA, Gibbs RA, Kucherlapati R, Lee C, Kharchenko PV, Park PJ, C.G.A.R.N. Landscape of somatic retrotransposition in human cancers. Science. 2012;337(6097):967–971. doi:10.1126/science.1222077. 10.1126/science.1222077 PMC3656569 22745252

[87] Criscione SW, Zhang Y, Thompson W, Sedivy JM, Neretti N (2014). Transcriptional landscape of repetitive elements in normal and cancer human cells. BMC Genomics.

[88] La Mantia G, Majello B, Di Cristofano A, Strazzullo M, Minchiotti G, Lania L (1992). Identification of regulatory elements within the minimal promoter region of the human endogenous ERV9 proviruses: accurate transcription initiation is controlled by an Inr-like element. Nucleic Acids Res.

[89] Yu X, Zhu X, Pi W, Ling J, Ko L, Takeda Y, Tuan D (2005). The long terminal repeat (LTR) of ERV-9 human endogenous retrovirus binds to NF-Y in the assembly of an active LTR enhancer complex NF-Y/MZF1/GATA-2. J Biol Chem.

[90] Ling J, Pi W, Bollag R, Zeng S, Keskintepe M, Saliman H, Krantz S, Whitney B, Tuan D (2002). The solitary long terminal repeats of ERV-9 endogenous retrovirus are conserved during primate evolution and possess enhancer activities in embryonic and hematopoietic cells. J Virol.

[91] Sokol M, Jessen KM, Pedersen FS (2015). Human endogenous retroviruses sustain complex and cooperative regulation of gene-containing loci and unannotated megabase-sized regions. Retrovirology.

[92] Whisnant AW, Bogerd HP, Flores O, Ho P, Powers JG, Sharova N, Stevenson M, Chen C-H, Cullen BR (2013). In-depth analysis of the interaction of HIV-1 with cellular microRNA biogenesis and effector mechanisms. MBio.

[93] Lahens NF, Kavakli IH, Zhang R, Hayer K, Black MB, Dueck H, Pizarro A, Kim J, Irizarry R, Thomas RS, Grant GR, Hogenesch JB (2014). IVT-seq reveals extreme bias in RNA sequencing. Genome Biol.

[94] Hockett RD, Kilby JM, Derdeyn CA, Saag MS, Sillers M, Squires K, Chiz S, Nowak MA, Shaw GM, Bucy RP (1999). Constant mean viral copy number per infected cell in tissues regardless of high, low, or undetectable plasma HIV RNA. J Exp Med.

[95] De Boer RJ, Ribeiro RM, Perelson AS (2010). Current estimates for HIV-1 production imply rapid viral clearance in lymphoid tissues. PLoS Comput Biol.

[96] Berry CC, Ocwieja K, Malani N, Bushman FD (2014). Comparing DNA integration site clusters with Scan Statistics. Bioinformatics.

[97] Pääbo S, Irwin DM, Wilson AC (1990). DNA damage promotes jumping between templates during enzymatic amplification. J Biol Chem.

[98] Odelberg SJ, Weiss RB, Hata A, White R (1995). Template-switching during DNA synthesis by Thermus aquaticus DNA polymerase I. Nucleic Acids Res.

[99] Zeng X-C, Wang S-X (2002). Evidence that BmTXK beta-BmKCT cDNA from Chinese scorpion Buthus martensii Karsch is an artifact generated in the reverse transcription process. FEBS Lett.

[100] Tasic B, Nabholz CE, Baldwin KK, Kim Y, Rueckert EH, Ribich SA, Cramer P, Wu Q, Axel R, Maniatis T (2002). Promoter choice determines splice site selection in protocadherin alpha and gamma pre-mRNA splicing. Mol Cell.

[101] Geiszt M, Lekstrom K, Leto TL (2004). Analysis of mRNA transcripts from the NAD(P)H oxidase 1 (Nox1) gene. Evidence against production of the NADPH oxidase homolog-1 short (NOH-1S) transcript variant. J Biol Chem.

[102] Cocquet J, Chong A, Zhang G, Veitia RA (2006). Reverse transcriptase template switching and false alternative transcripts. Genomics.

[103] McManus CJ, Coolon JD, Duff MO, Eipper-Mains J, Graveley BR, Wittkopp PJ (2010). Regulatory divergence in Drosophila revealed by mRNA-seq. Genome Res.

[104] Cogné B, Snyder R, Lindenbaum P, Dupont J-B, Redon R, Moullier P, Leger A (2014). NGS library preparation may generate artifactual integration sites of AAV vectors. Nat Med.

[105] Gilboa E, Mitra SW, Goff S, Baltimore D (1979). A detailed model of reverse transcription and tests of crucial aspects. Cell.

[106] Luo GX, Taylor J (1990). Template switching by reverse transcriptase during DNA synthesis. J Virol.

[107] Houseley J, Tollervey D (2010). Apparent non-canonical trans-splicing is generated by reverse transcriptase in vitro. PLoS One.

[108] Meyerhans A, Vartanian JP, Wain-Hobson S (1990). DNA recombination during PCR. Nucleic Acids Res.

[109] Lahr DJG, Katz LA (2009). Reducing the impact of PCR-mediated recombination in molecular evolution and environmental studies using a new-generation high-fidelity DNA polymerase. Biotechniques.

[110] Brady T, Agosto LM, Malani N, Berry CC, O’Doherty U, Bushman F (2009). HIV integration site distributions in resting and activated CD4+ T cells infected in culture. AIDS.

[111] Magrane M, UniProt Consortium. UniProt Knowledgebase: a hub of integrated protein data. Database (Oxford). 2011;2011:009. doi:10.1093/database/bar009. 10.1093/database/bar009 PMC3070428 21447597

[112] UniProt Consortium. UniProt: a hub for protein information. Nucleic Acids Res. 2015;43(Database issue):204–212. doi:10.1093/nar/gku989. 10.1093/nar/gku989 PMC4384041 25348405

[113] Wang GP, Ciuffi A, Leipzig J, Berry CC, Bushman FD (2007). HIV integration site selection: analysis by massively parallel pyrosequencing reveals association with epigenetic modifications. Genome Res.

[114] Maldarelli F, Xiang C, Chamoun G, Zeichner SL (1998). The expression of the essential nuclear splicing factor SC35 is altered by human immunodeficiency virus infection. Virus Res.

[115] Monette A, Ajamian L, López-Lastra M, Mouland AJ (2009). Human immunodeficiency virus type 1 (HIV-1) induces the cytoplasmic retention of heterogeneous nuclear ribonucleoprotein A1 by disrupting nuclear import: implications for HIV-1 gene expression. J Biol Chem.

[116] Li XL, Blackford JA, Judge CS, Liu M, Xiao W, Kalvakolanu DV, Hassel BA (2000). RNase-L-dependent destabilization of interferon-induced mRNAs. A role for the 2–5A system in attenuation of the interferon response. J Biol Chem.

[117] Al-Ahmadi W, Al-Haj L, Al-Mohanna FA, Silverman RH, Khabar KSA (2009). RNase L downmodulation of the RNA-binding protein, HuR, and cellular growth. Oncogene.

[118] Malim MH, Hauber J, Le SY, Maizel JV, Cullen BR (1989). The HIV-1 rev trans-activator acts through a structured target sequence to activate nuclear export of unspliced viral mRNA. Nature.

[119] Malim MH, Böhnlein S, Hauber J, Cullen BR (1989). Functional dissection of the HIV-1 Rev trans-activator-derivation of a trans-dominant repressor of Rev function. Cell.

[120] Mitrovich QM, Anderson P (2000). Unproductively spliced ribosomal protein mRNAs are natural targets of mRNA surveillance in C. elegans. Genes Dev.

[121] Cuccurese M, Russo G, Russo A, Pietropaolo C (2005). Alternative splicing and nonsense-mediated mRNA decay regulate mammalian ribosomal gene expression. Nucleic Acids Res.

[122] Malygin AA, Parakhnevitch NM, Ivanov AV, Eperon IC, Karpova GG (2007). Human ribosomal protein S13 regulates expression of its own gene at the splicing step by a feedback mechanism. Nucleic Acids Res.

[123] Wreschner DH, James TC, Silverman RH, Kerr IM (1981). Ribosomal RNA cleavage, nuclease activation and 2–5A(ppp(A2’p)nA) in interferon-treated cells. Nucleic Acids Res.

[124] Cooper DA, Jha BK, Silverman RH, Hesselberth JR, Barton DJ (2014). Ribonuclease L and metal-ion-independent endoribonuclease cleavage sites in host and viral RNAs. Nucleic Acids Res.

[125] Gupta R, Kim S, Taylor MW (2012). Suppression of ribosomal protein synthesis and protein translation factors by Peg-interferon alpha/ribavirin in HCV patients blood mononuclear cells (PBMC). J Transl Med.

[126] Henig N, Avidan N, Mandel I, Staun-Ram E, Ginzburg E, Paperna T, Pinter RY, Miller A (2013). Interferon-beta induces distinct gene expression response patterns in human monocytes versus T cells. PLoS One.

[127] Boller K, Janssen O, Schuldes H, Tönjes RR, Kurth R (1997). Characterization of the antibody response specific for the human endogenous retrovirus HTDV/HERV-K. J Virol.

[128] Pi W, Yang Z, Wang J, Ruan L, Yu X, Ling J, Krantz S, Isales C, Conway SJ, Lin S, Tuan D (2004). The LTR enhancer of ERV-9 human endogenous retrovirus is active in oocytes and progenitor cells in transgenic zebrafish and humans. Proc Natl Acad Sci USA.

[129] Zhang XH-F, Chasin LA (2006). Comparison of multiple vertebrate genomes reveals the birth and evolution of human exons. Proc Natl Acad Sci USA.

[130] Zeng M, Hu Z, Shi X, Li X, Zhan X, Li XD, Wang J, Choi JH, Wang KW, Purrington T, Tang M, Fina M, DeBerardinis RJ, Moresco EMY, Pedersen G, McInerney GM, Karlsson Hedestam GB, Chen ZJ, Beutler B. MAVS, cGAS, and endogenous retroviruses in T-independent B cell responses. Science. 2014;346(6216):1486–1492. doi:10.1126/science.346.6216.1486. 10.1126/science.346.6216.1486 PMC4391621 25525240

[131] Grow EJ, Flynn RA, Chavez SL, Bayless NL, Wossidlo M, Wesche DJ, Martin L, Ware CB, Blish CA, Chang HY, Pera RAR, Wysocka J (2015). Intrinsic retroviral reactivation in human preimplantation embryos and pluripotent cells. Nature.

[132] Smith J, Azad A, Deacon N (1992). Identification of two novel human immunodeficiency virus type 1 splice acceptor sites in infected T cell lines. J Gen Virol.

[133] Carrera C, Pinilla M, Pérez-Alvarez L, Thomson MM (2010). Identification of unusual and novel HIV type 1 spliced transcripts generated in vivo. AIDS Res Hum Retroviruses.

[134] Pollard VW, Malim MH (1998). The HIV-1 Rev protein. Annu Rev Microbiol.

[135] Rosenberg N, Jolicoeur P. Retroviral pathogenesis. In: Coffin J, Hughes S, Varmus H, editors. Retroviruses. Cold Spring Harbor: Cold Spring Harbor Laboratory Press; 1997. http://www.ncbi.nlm.nih.gov/books/NBK19378/. 21433341

[136] Ott MG, Schmidt M, Schwarzwaelder K, Stein S, Siler U, Koehl U, Glimm H, Kühlcke K, Schilz A, Kunkel H, Naundorf S, Brinkmann A, Deichmann A, Fischer M, Ball C, Pilz I, Dunbar C, Du Y, Jenkins NA, Copeland NG, Lüthi U, Hassan M, Thrasher AJ, Hoelzer D, von Kalle C, Seger R, Grez M (2006). Correction of X-linked chronic granulomatous disease by gene therapy, augmented by insertional activation of MDS1-EVI1, PRDM16 or SETBP1. Nat Med.

[137] Braun CJ, Boztug K, Paruzynski A, Witzel M, Schwarzer A, Rothe M, Modlich U, Beier R, Göhring G, Steinemann D, Fronza R, Ball CR, Haemmerle R, Naundorf S, Kühlcke K, Rose M, Fraser C, Mathias L, Ferrari R, Abboud MR, Al-Herz W, Kondratenko I, Maródi L, Glimm H, Schlegelberger B, Schambach A, Albert MH, Schmidt M, von Kalle C, Klein C (2014). Gene therapy for Wiskott-Aldrich syndrome-long-term efficacy and genotoxicity. Sci Transl Med.

[138] Santoni FA, Guerra J, Luban J (2012). HERV-H RNA is abundant in human embryonic stem cells and a precise marker for pluripotency. Retrovirology.

[139] Fuchs NV, Loewer S, Daley GQ, Izsvák Z, Löwer J, Löwer R (2013). Human endogenous retrovirus K (HML-2) RNA and protein expression is a marker for human embryonic and induced pluripotent stem cells. Retrovirology.

[140] Fort A, Hashimoto K, Yamada D, Salimullah M, Keya CA, Saxena A, Bonetti A, Voineagu I, Bertin N, Kratz A, Noro Y, Wong C-H, de Hoon M, Andersson R, Sandelin A, Suzuki H, Wei C-L, Koseki H; F.A.N.T.O.M.C., Hasegawa Y, Forrest AR, Carninci P. Deep transcriptome profiling of mammalian stem cells supports a regulatory role for retrotransposons in pluripotency maintenance. Nat Genet. 2014;46(6):558–66. doi:10.1038/ng.2965. 10.1038/ng.2965 24777452

[141] Wang J, Xie G, Singh M, Ghanbarian AT, Raskó T, Szvetnik A, Cai H, Besser D, Prigione A, Fuchs NV, Schumann GG, Chen W, Lorincz MC, Ivics Z, Hurst LD, Izsvák Z (2014). Primate-specific endogenous retrovirus-driven transcription defines naive-like stem cells. Nature.

[142] Joos B, Fischer M, Kuster H, Pillai SK, Wong JK, Böni J, Hirschel B, Weber R, Trkola A, Günthard HF; S.H.I.V.C.S. HIV rebounds from latently infected cells, rather than from continuing low-level replication. Proc Natl Acad Sci USA. 2008;105(43):16725–30. doi:10.1073/pnas.0804192105. 10.1073/pnas.0804192105 PMC2575487 18936487

[143] Brennan TP, Woods JO, Sedaghat AR, Siliciano JD, Siliciano RF, Wilke CO (2009). Analysis of human immunodeficiency virus type 1 viremia and provirus in resting CD4+ T cells reveals a novel source of residual viremia in patients on antiretroviral therapy. J Virol.

[144] Wagner TA, McKernan JL, Tobin NH, Tapia KA, Mullins JI, Frenkel LM (2013). An increasing proportion of monotypic HIV-1 DNA sequences during antiretroviral treatment suggests proliferation of HIV-infected cells. J Virol.

[145] Kearney MF, Spindler J, Shao W, Yu S, Anderson EM, O’Shea A, Rehm C, Poethke C, Kovacs N, Mellors JW, Coffin JM, Maldarelli F (2014). Lack of detectable HIV-1 molecular evolution during suppressive antiretroviral therapy. PLoS Pathog.

[146] Kent WJ (2002). BLAT-the BLAST-like alignment tool. Genome Res.

[147] Langmead B, Trapnell C, Pop M, Salzberg SL (2009). Ultrafast and memory-efficient alignment of short DNA sequences to the human genome. Genome Biol.

[148] Grant GR, Farkas MH, Pizarro AD, Lahens NF, Schug J, Brunk BP, Stoeckert CJ, Hogenesch JB, Pierce EA (2011). Comparative analysis of RNA-Seq alignment algorithms and the RNA-Seq unified mapper (RUM). Bioinformatics.

[149] Trapnell C, Williams BA, Pertea G, Mortazavi A, Kwan G, van Baren MJ, Salzberg SL, Wold BJ, Pachter L (2010). Transcript assembly and quantification by RNA-Seq reveals unannotated transcripts and isoform switching during cell differentiation. Nat Biotechnol.

[150] R Core Team. R: a language and environment for statistical computing. Vienna: R Foundation for Statistical Computing; 2012. R Foundation for Statistical Computing.

[151] Kent WJ, Sugnet CW, Furey TS, Roskin KM, Pringle TH, Zahler AM, Haussler D (2002). The human genome browser at UCSC. Genome Res.

[152] Li H, Handsaker B, Wysoker A, Fennell T, Ruan J, Homer N, Marth G, Abecasis G, Durbin R; G.P.D.P.S. The sequence alignment/map format and SAMtools. Bioinformatics. 2009;25(16):2078–2079. doi:10.1093/bioinformatics/btp352. 10.1093/bioinformatics/btp352 PMC2723002 19505943

[153] Friedman J, Hastie T, Tibshirani R (2010). Regularization paths for generalized linear models via coordinate descent. J Stat Softw.

[154] La Mantia G, Maglione D, Pengue G, Di Cristofano A, Simeone A, Lanfrancone L, Lania L (1991). Identification and characterization of novel human endogenous retroviral sequences prefentially expressed in undifferentiated embryonal carcinoma cells. Nucleic Acids Res.

[155] Plant KE, Routledge SJ, Proudfoot NJ (2001). Intergenic transcription in the human beta-globin gene cluster. Mol Cell Biol.

[156] Edgar RC (2004). MUSCLE: a multiple sequence alignment method with reduced time and space complexity. BMC Bioinform..

