# Supplementary material for: Gene activity in primary T cells infected with HIV89.6: intron retention and induction of genomic repeats
Source: Retrovirology. 2015 Sep 17;12:79. doi: 10.1186/s12977-015-0205-1 (PMC4574318; doi:10.1186/s12977-015-0205-1)
Supplement: Supplementary file 5 — 10.1186/s12977-015-0205-1 Expression observed in the top 50 most differentially expressed introns in ribosomal protein genes and ERV-9/LTR12C regions with expression continuing for greater than 1000bp downstream. A) RNA-Seq read counts for the 50 most differentially expressed in introns in ribosomal protein genes. The number of reads covering each base is shown on the y-axis. UCSC genes and RepeatMasker LTRs are annotated below with lines indicating introns and boxes indicating exons or repeats. Dashed vertical lines highlight the region detected as differentially expressed. B) As in A but showing RNA-Seq read counts for ERV-9/LTR12C with expression continuing for greater than 1000 bp 3′ of the LTR region. LTRs were selected by filtering for regions with a 90th percentile coverage higher than 30 and looking for the furthest 3′ base with continuous (no gap greater than 200 bp) coverage of at least 10 % of the 90th percentile of expression within the LTR. [file 12977_2015_205_MOESM5_ESM.pdf]

A.1

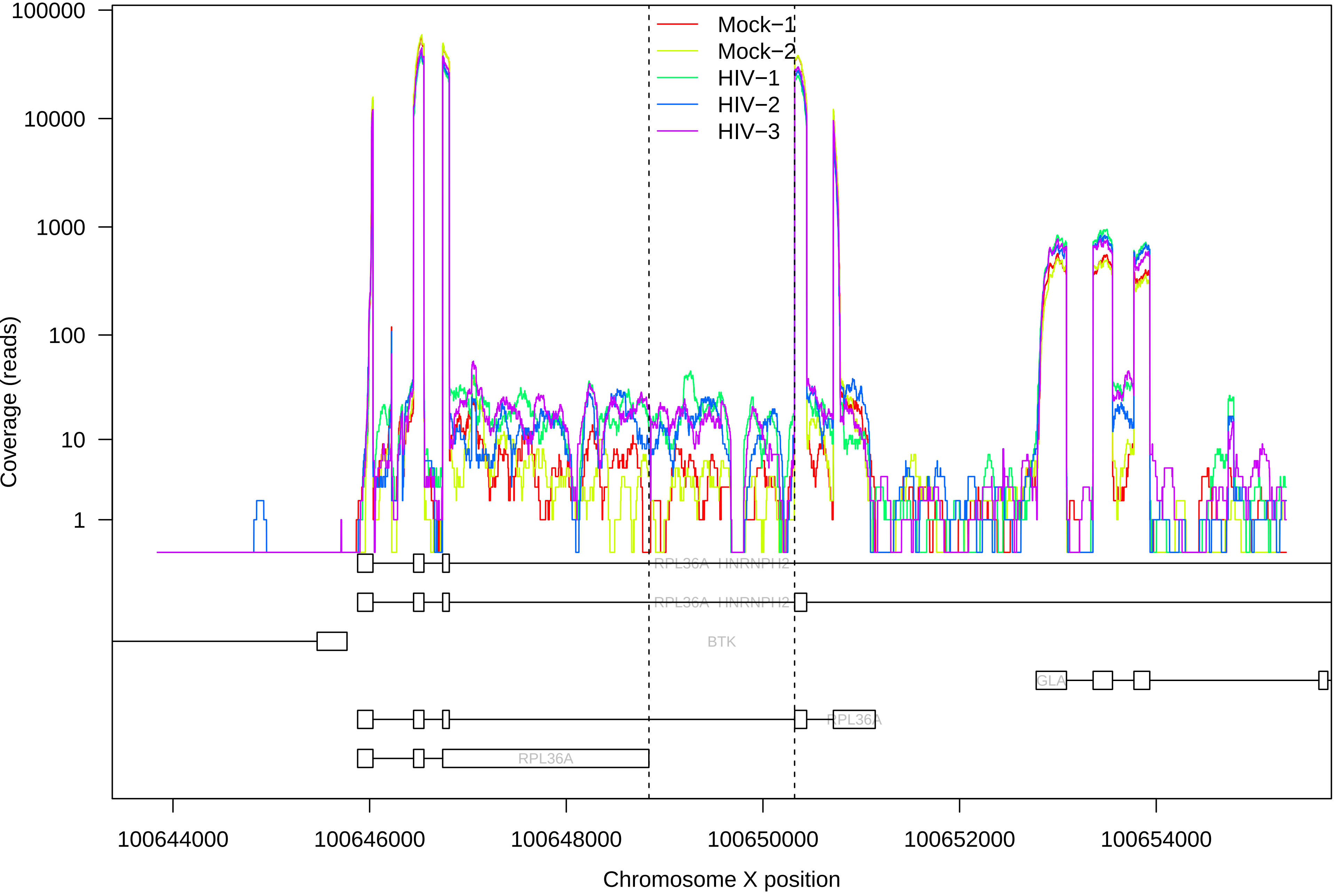
chrX:100648841−100650322
Coverage (reads)
100000
10000
1000
100
10
1
Mock−1
Mock−2
HIV−1
HIV−2
HIV−3
RPL36A−HNRNPH2
RPL36A−HNRNPH2
BTK
GLA
RPL36A
RPL36A
100644000
100646000
100648000
100650000
100652000
100654000
Chromosome X position

A.2

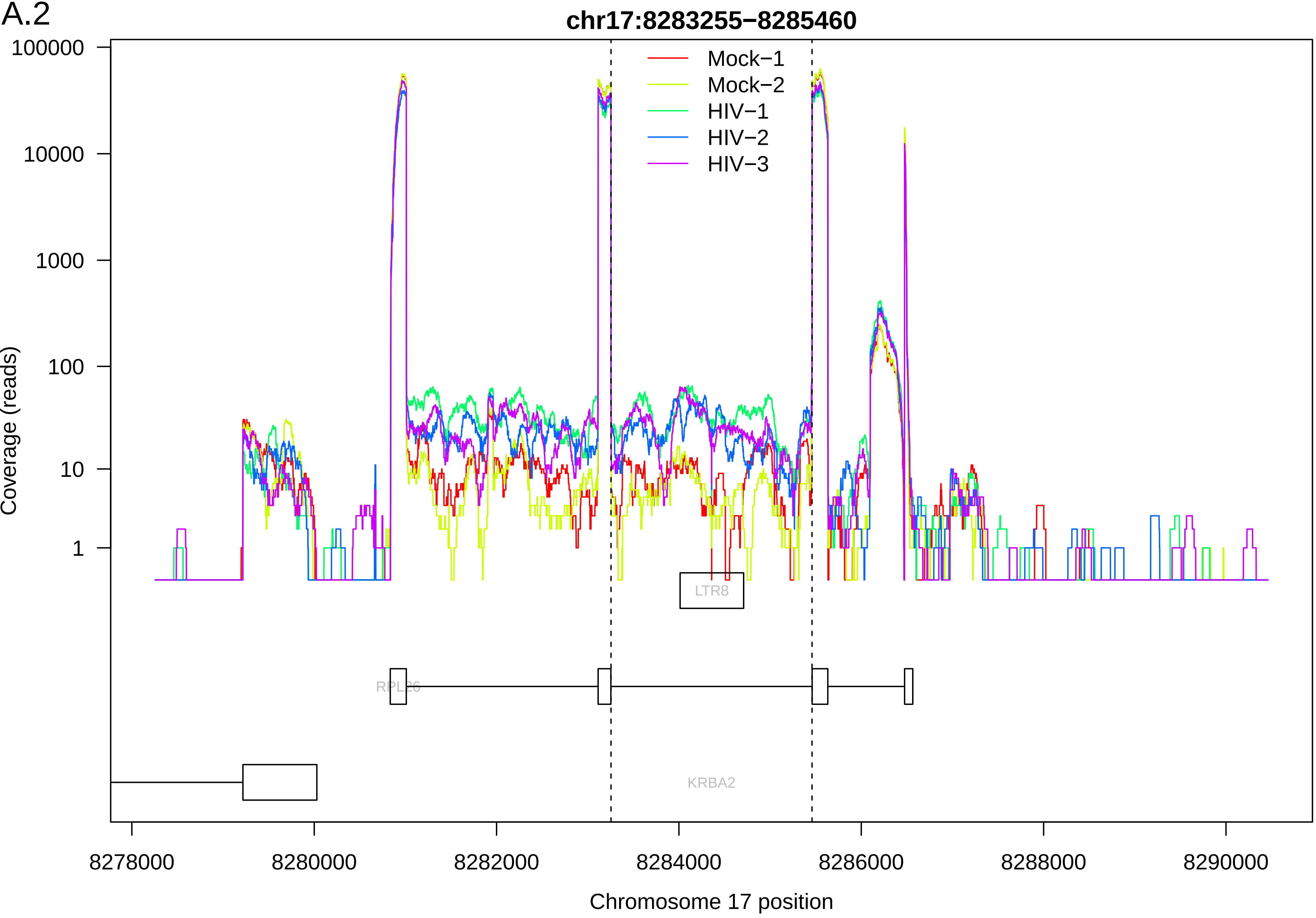

chr17:8283255−8285460
Mock−1
Mock−2
HIV−1
HIV−2
HIV−3
100000
10000
1000
100
10
1
Coverage (reads)
LTR8
RPL26
KRBA2
8278000
8280000
8282000
8284000
8286000
8288000
8290000
Chromosome 17 position

A.3

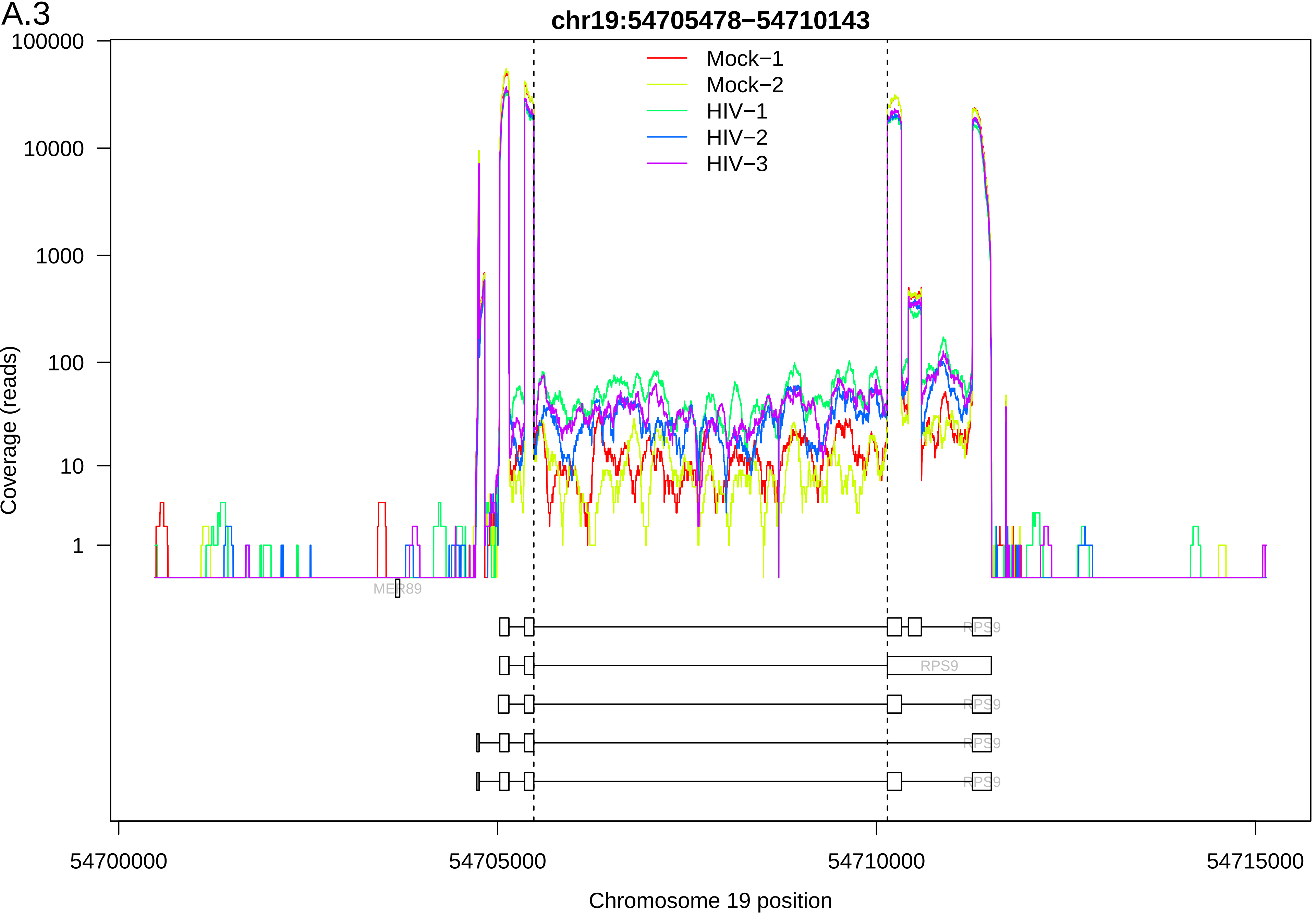
chr19:54705478−54710143
Mock−1
Mock−2
HIV−1
HIV−2
HIV−3
Coverage (reads)
100000
10000
1000
100
10
1
MER89
RPS9
RPS9
RPS9
RPS9
RPS9
54700000
54705000
54710000
54715000
Chromosome 19 position

A.4

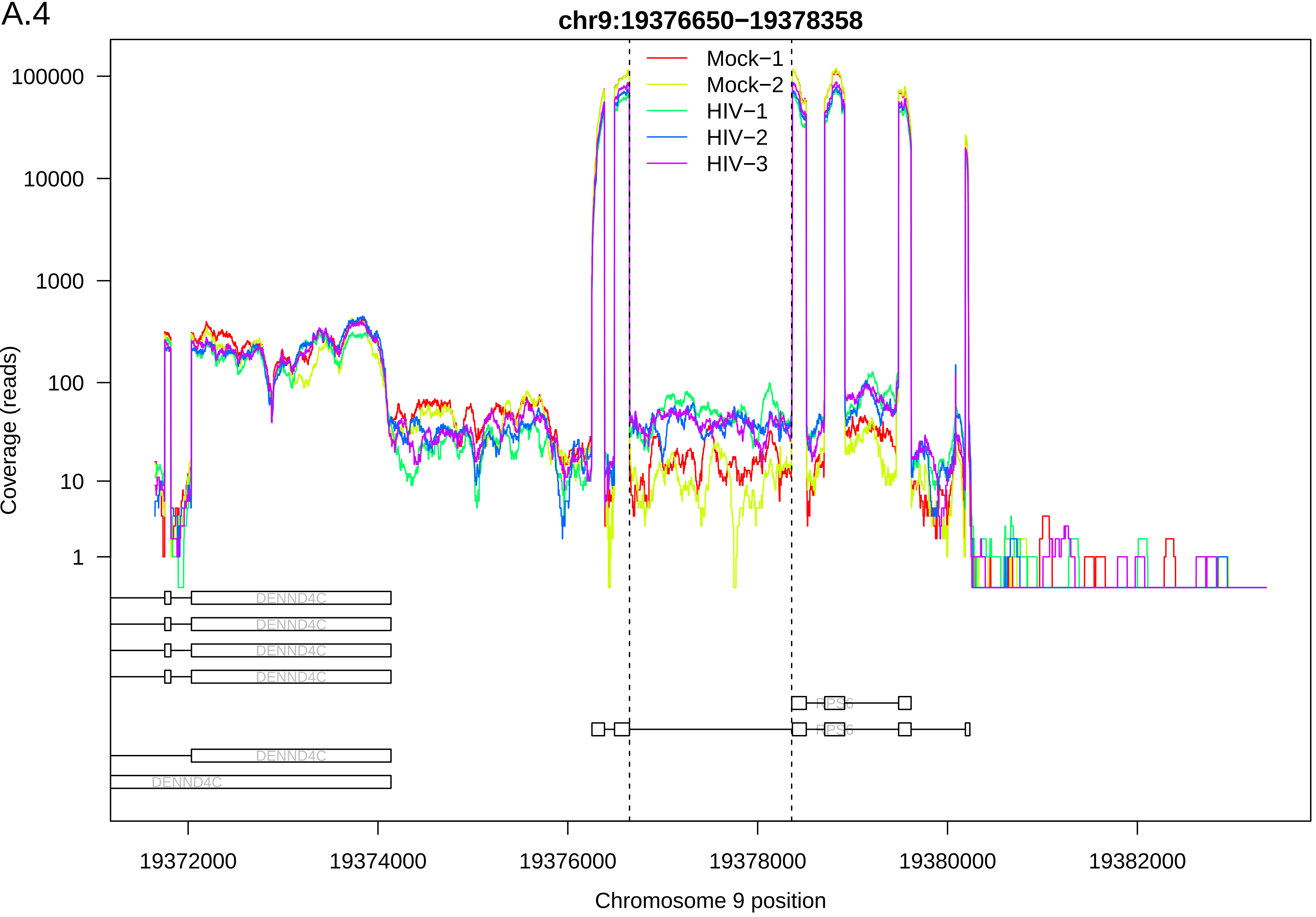
chr9:19376650−19378358
Mock−1
Mock−2
HIV−1
HIV−2
HIV−3
100000
10000
1000
100
10
1
Coverage (reads)
DENND4C
DENND4C
DENND4C
DENND4C
RPS6
RPS6
DENND4C
DENND4C
19372000
19374000
19376000
19378000
19380000
19382000
Chromosome 9 position

A.5

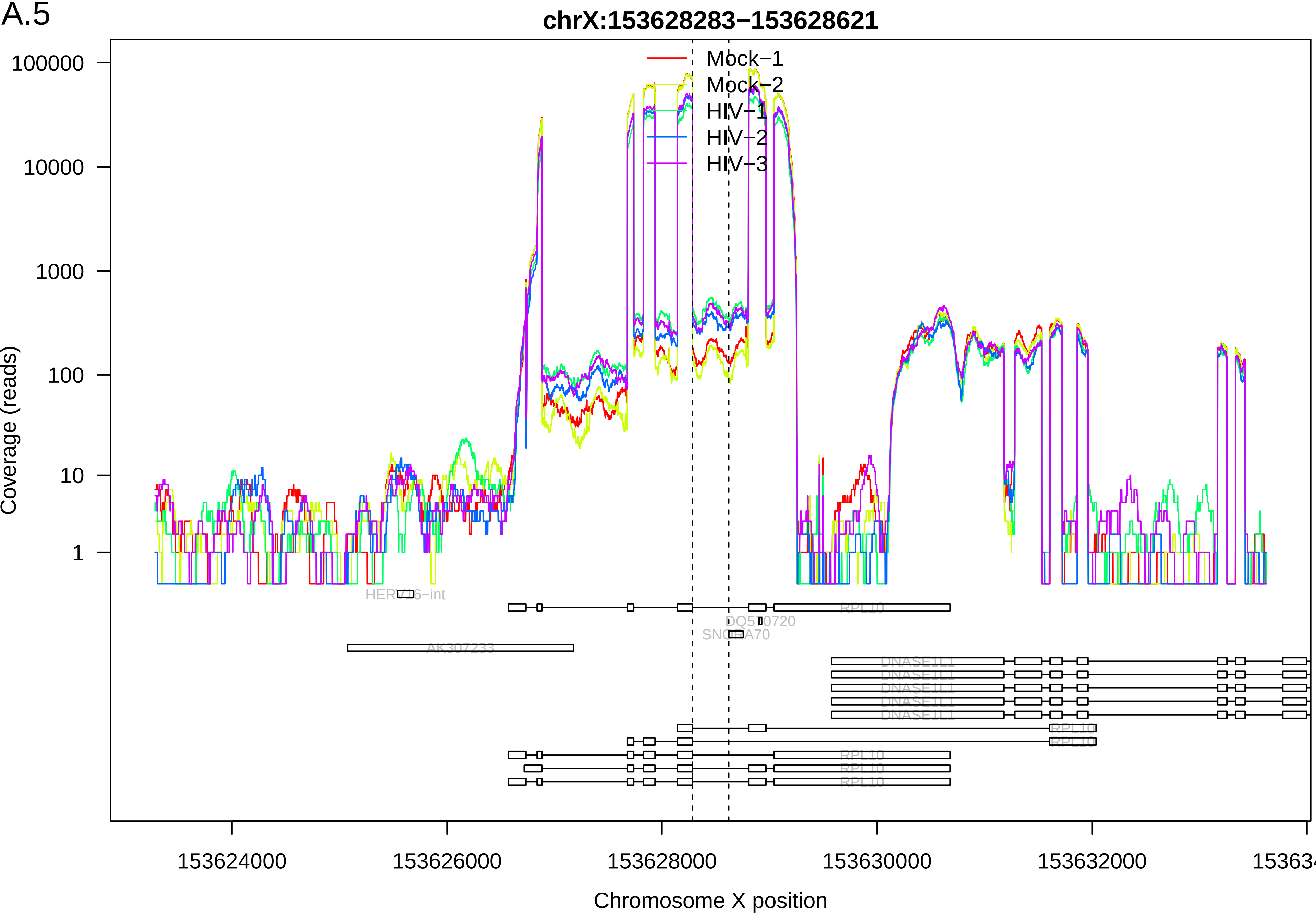
chrX:153628283−153628621
Mock−1
Mock−2
HIV−1
HIV−2
HIV−3
100000
10000
1000
100
10
1
Coverage (reads)
HERV16−int
RPL10
DQ570720
SNORA70
AK307233
DNASE1L1
153624000
153626000
153628000
153630000
153632000
Chromosome X position

A.6

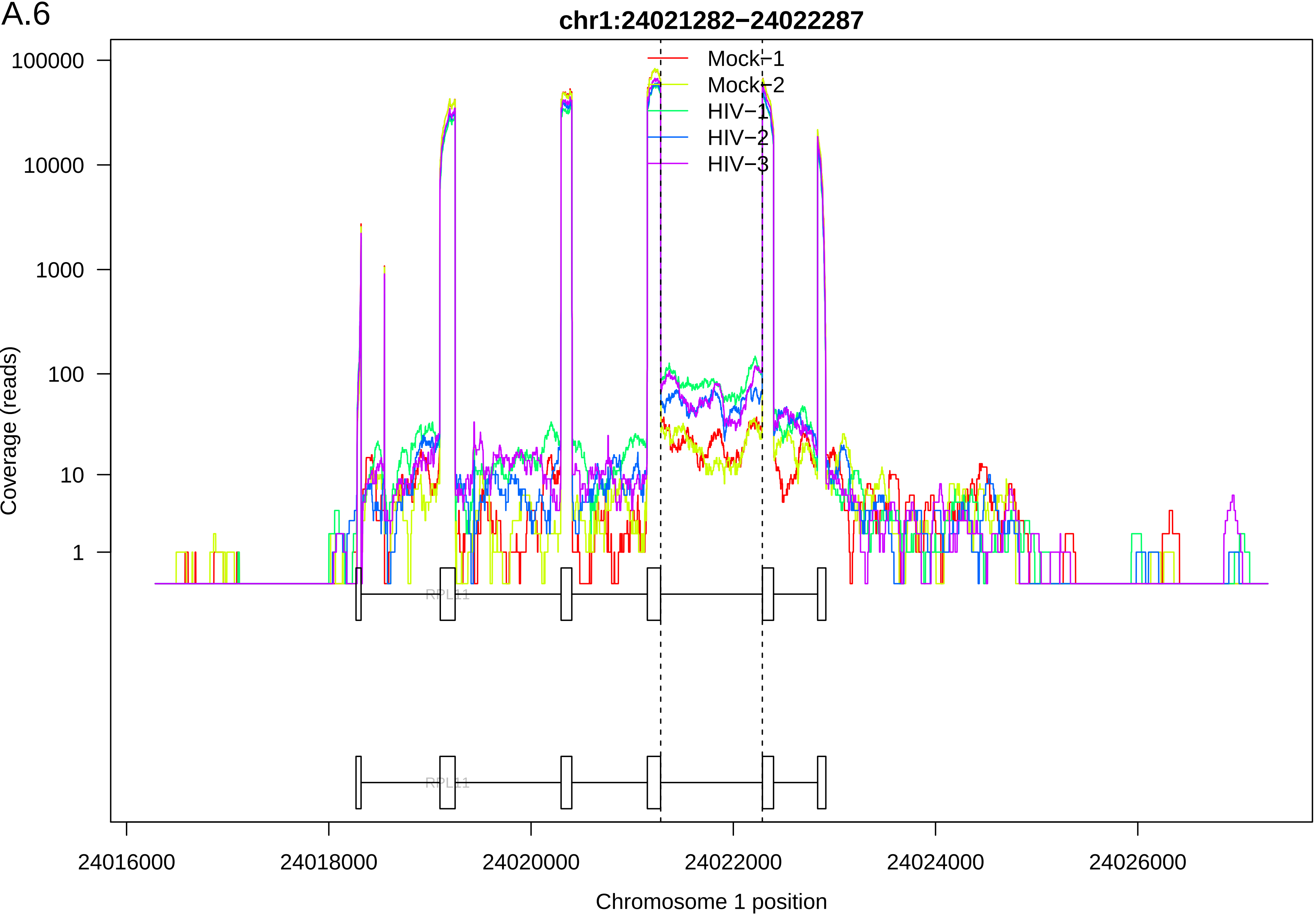
chr1:24021282−24022287
Mock−1
Mock−2
HIV−1
HIV−2
HIV−3
100000
10000
1000
100
10
1
Coverage (reads)
RPL11
RPL11
24016000
24018000
24020000
24022000
24024000
24026000
Chromosome 1 position

A.7

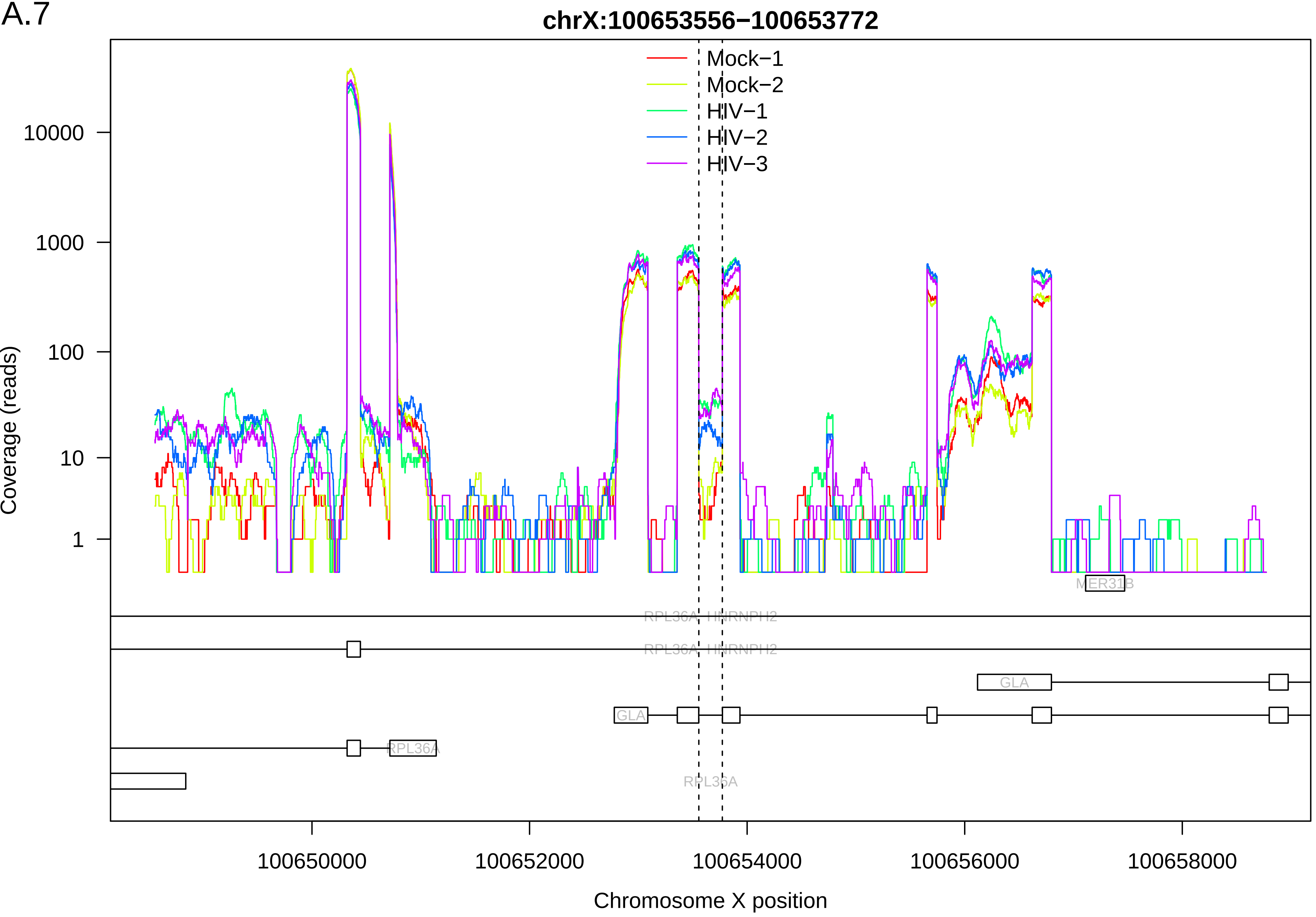
chrX:100653556−100653772
Mock−1
Mock−2
HIV−1
HIV−2
HIV−3
10000
1000
100
10
1
Coverage (reads)
MER31B
RPL36A−HNRNPH2
RPL36A−HNRNPH2
GLA
GLA
RPL36A
RPL36A
100650000
100652000
100654000
100656000
100658000
Chromosome X position

A.8

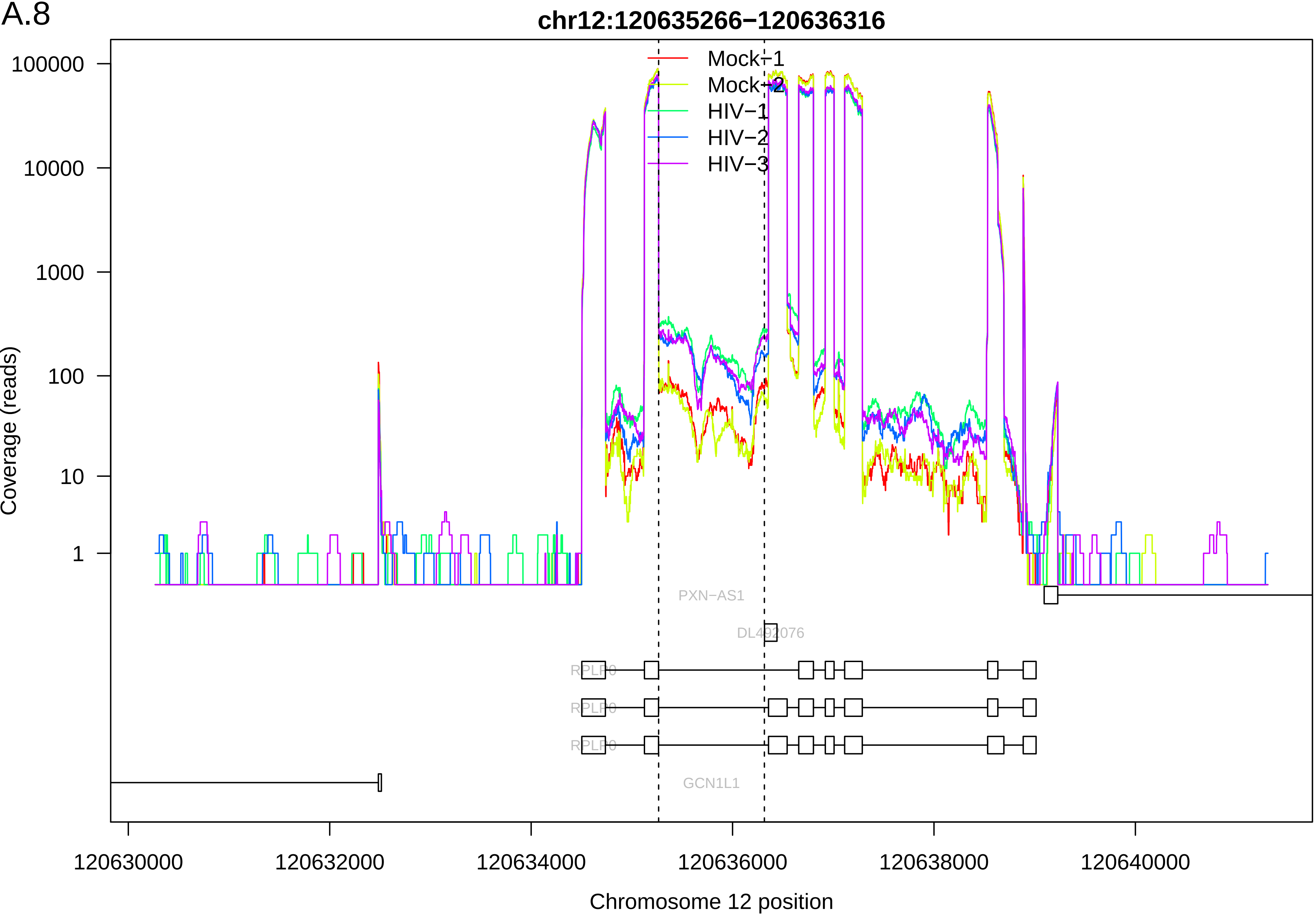
chr12:120635266−120636316
Mock−1
Mock−2
HIV−1
HIV−2
HIV−3
Coverage (reads)
100000
10000
1000
100
10
1
PXN−AS1
DL492076
RPLP0
RPLP0
RPLP0
GCN1L1
120630000
120632000
120634000
120636000
120638000
120640000
Chromosome 12 position

A.9

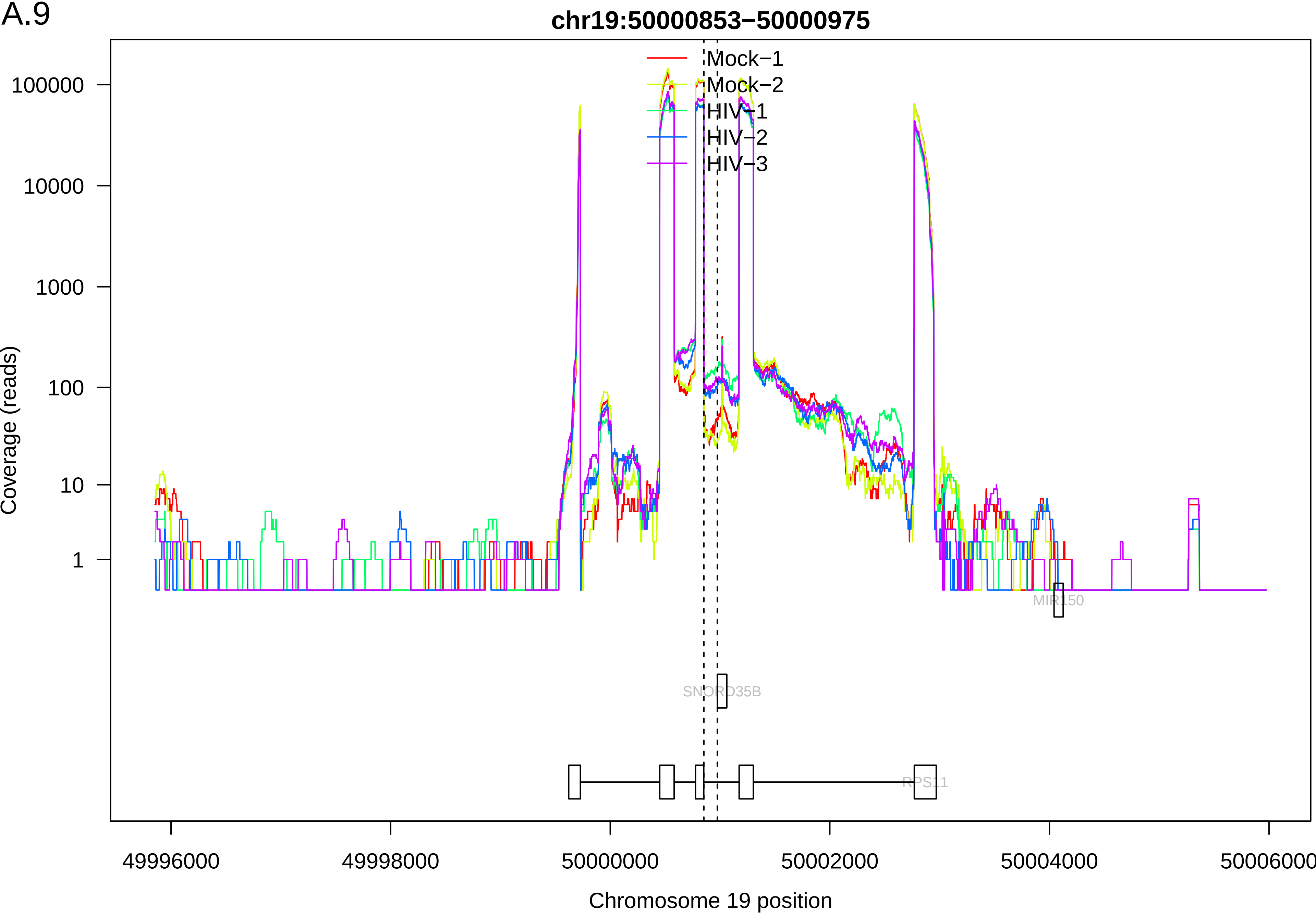
chr19:50000853−50000975
Mock−1
Mock−2
HIV−1
HIV−2
HIV−3
100000
10000
1000
100
10
1
Coverage (reads)
MIR150
SNORD35B
RPS11
49996000
49998000
50000000
50002000
50004000
50006000
Chromosome 19 position

A.10

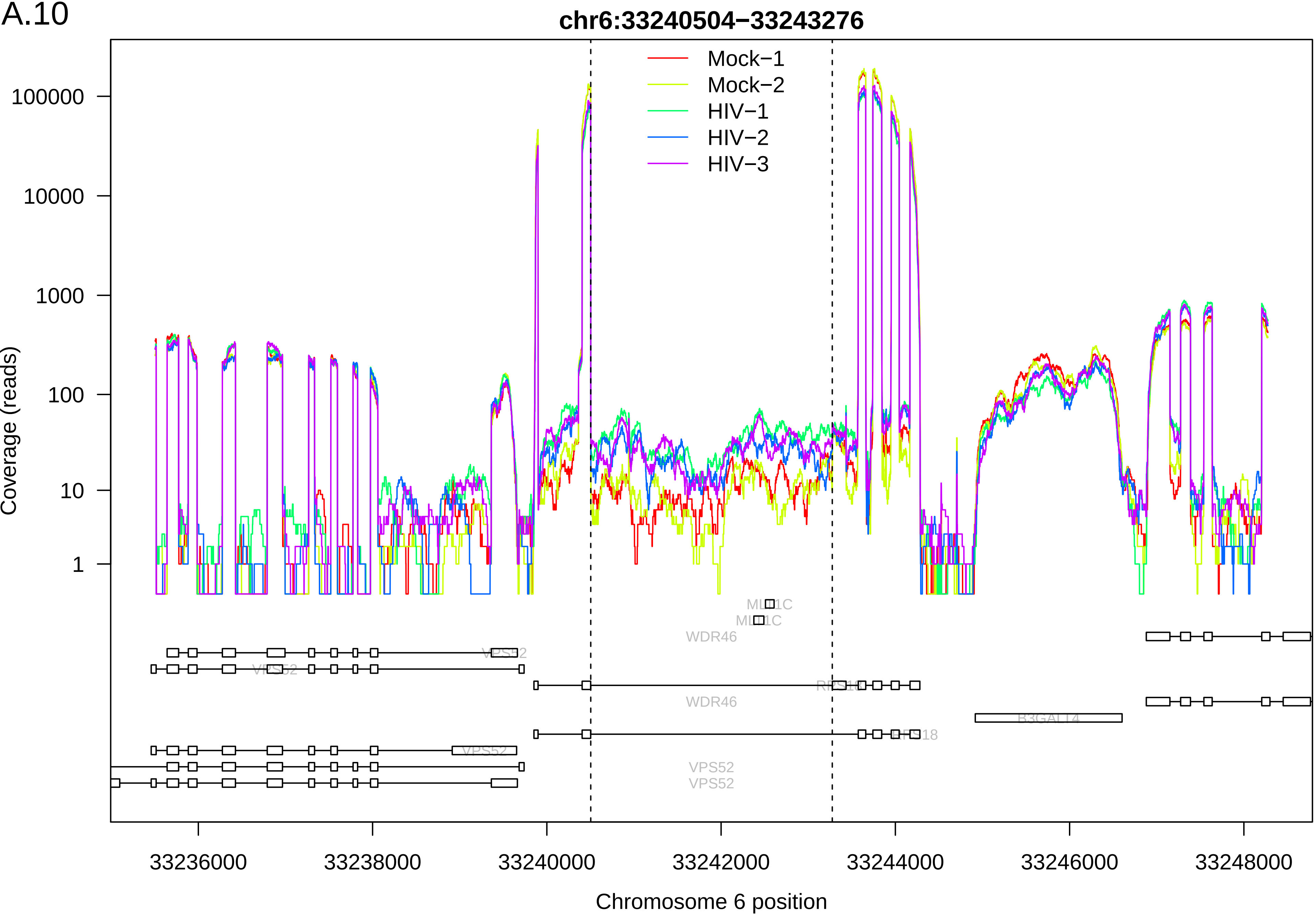

chr6:33240504−33243276
Mock−1
Mock−2
HIV−1
HIV−2
HIV−3
100000
10000
1000
100
10
1
Coverage (reads)
MLN1C
MLN1C
WDR46
VPS52
VPS52
RPS18
WDR46
B3GALT4
RPS18
VPS52
VPS52
VPS52
33236000
33238000
33240000
33242000
33244000
33246000
33248000
Chromosome 6 position

A.11

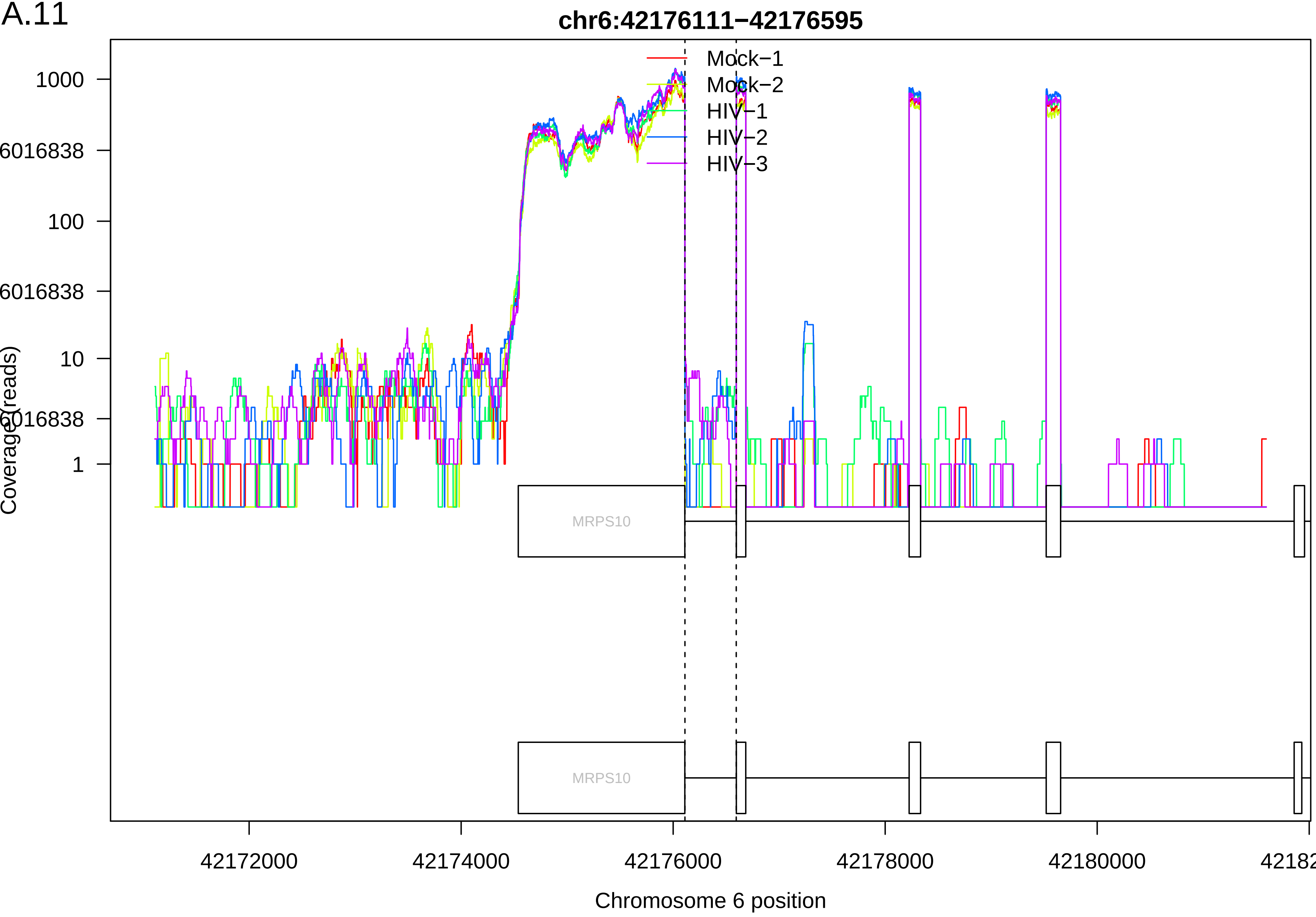
chr6:42176111−42176595
Mock−1
Mock−2
HIV−1
HIV−2
HIV−3
1000
6016838
100
6016838
10
6016838
1
Coverage (reads)
MRPS10
MRPS10
42172000
42174000
42176000
42178000
42180000
Chromosome 6 position

A.12

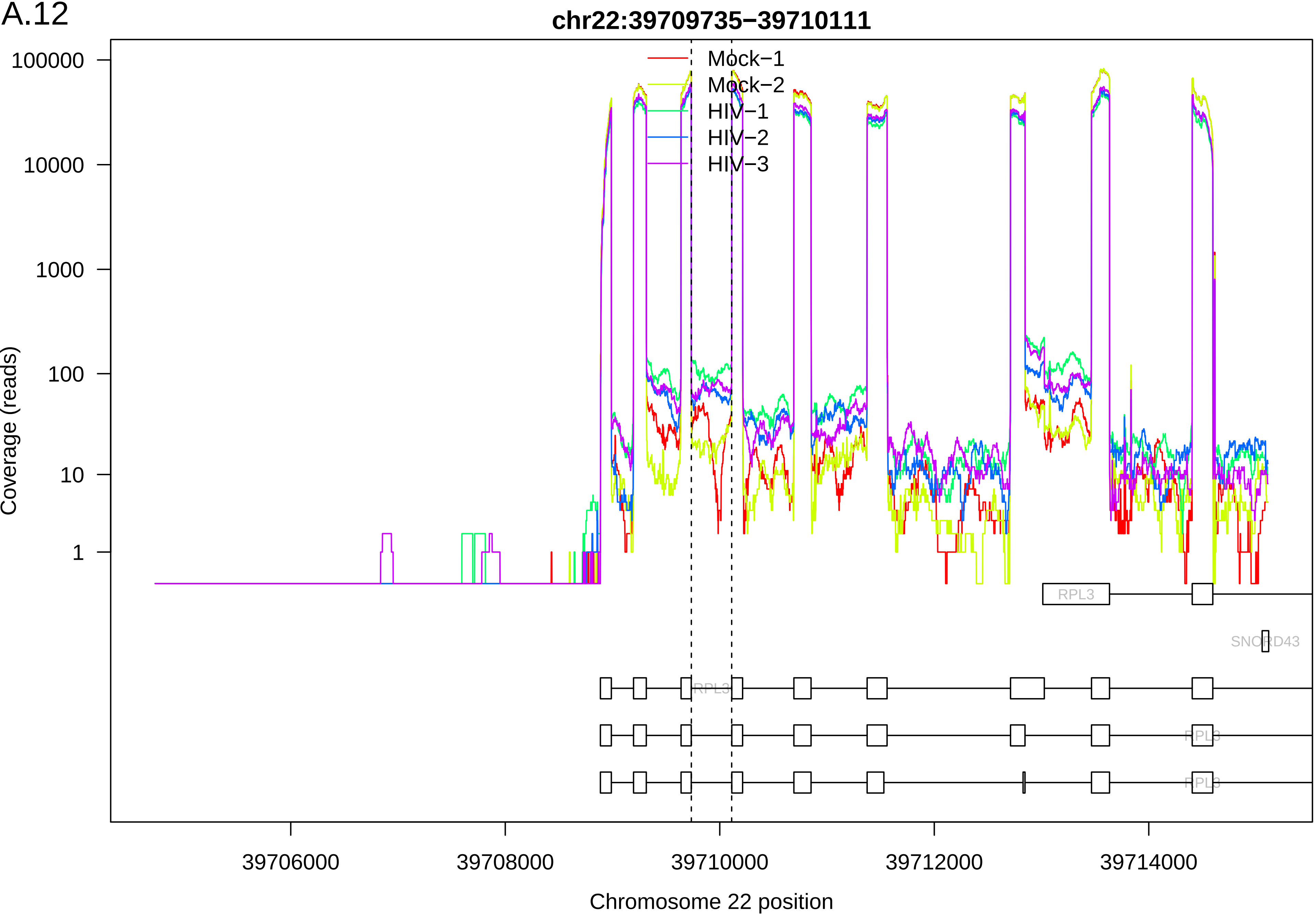

chr22:39709735−39710111
Mock−1
Mock−2
HIV−1
HIV−2
HIV−3
Coverage (reads)
100000
10000
1000
100
10
1
RPL3
SNORD43
RPL3
RPL3
RPL3
39706000
39708000
39710000
39712000
39714000
Chromosome 22 position

A.13

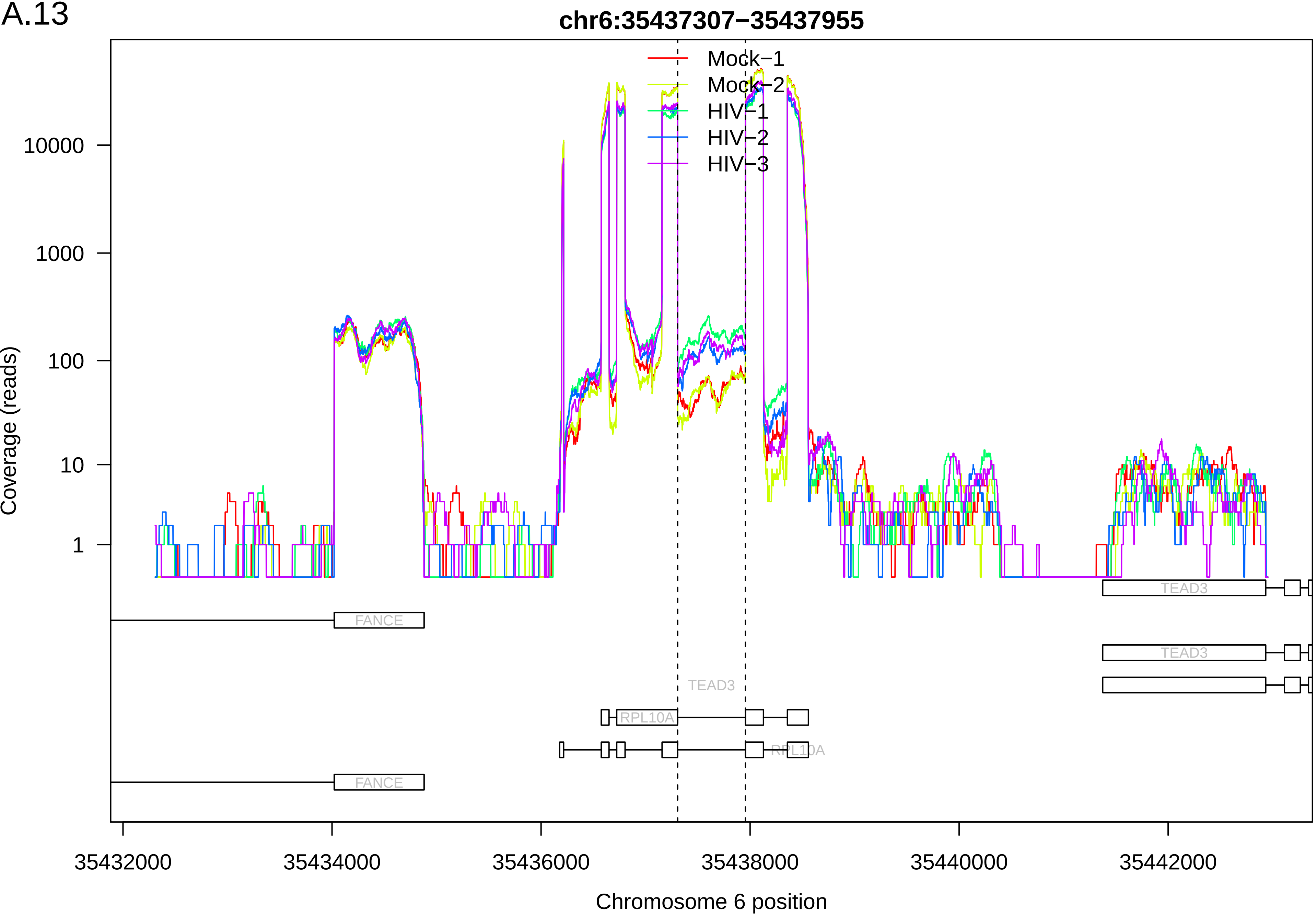
chr6:35437307−35437955
Mock−1
Mock−2
HIV−1
HIV−2
HIV−3
Coverage (reads)
10000
1000
100
10
1
TEAD3
FANCE
TEAD3
TEAD3
RPL10A
RPL10A
FANCE
35432000
35434000
35436000
35438000
35440000
35442000
Chromosome 6 position

A.14

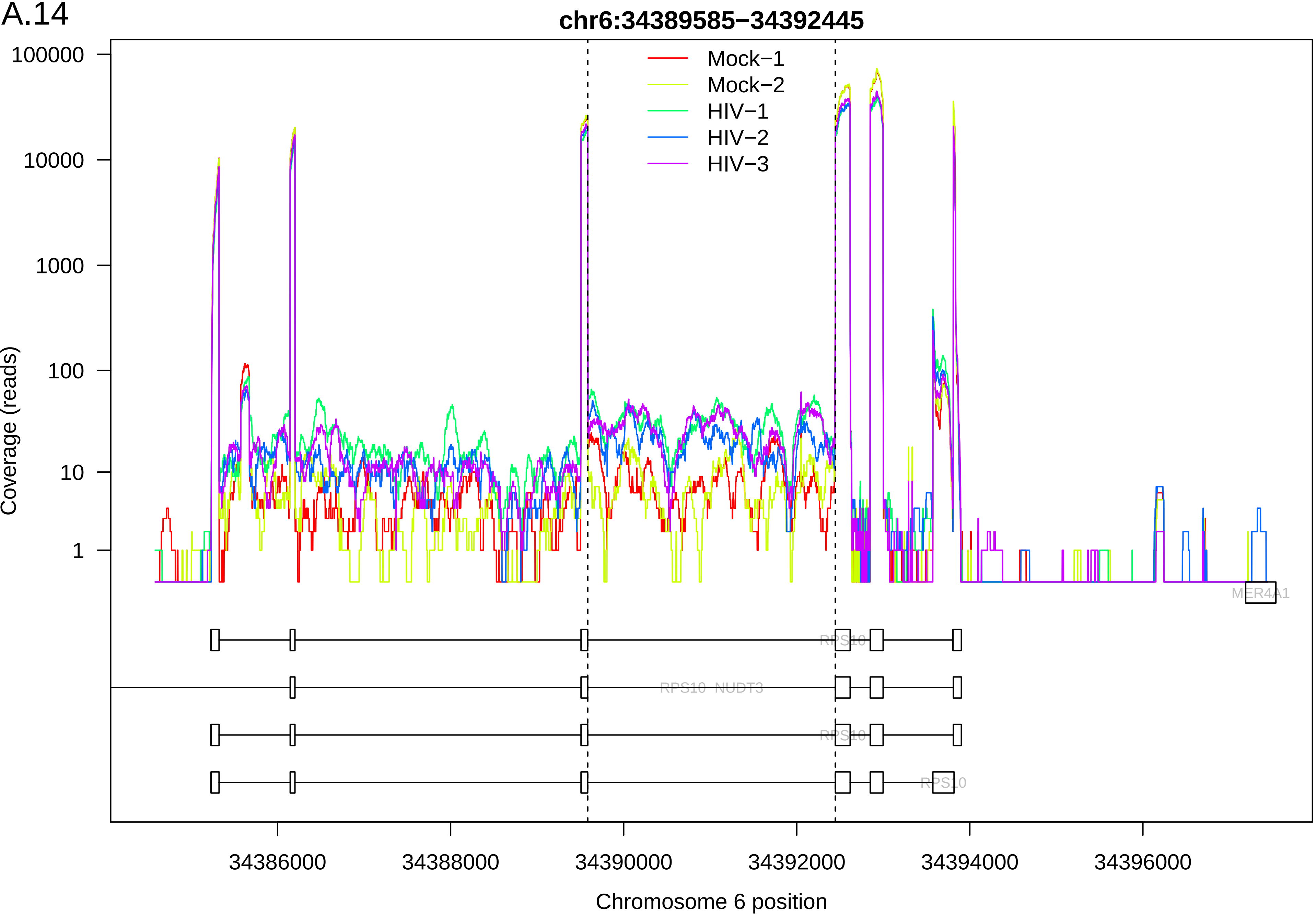

chr6:34389585−34392445
Mock−1
Mock−2
HIV−1
HIV−2
HIV−3
Coverage (reads)
100000
10000
1000
100
10
1
MER4A1
RPS10
RPS10−NUDT3
RPS10
RPS10
34386000
34388000
34390000
34392000
34394000
34396000
Chromosome 6 position

A.15

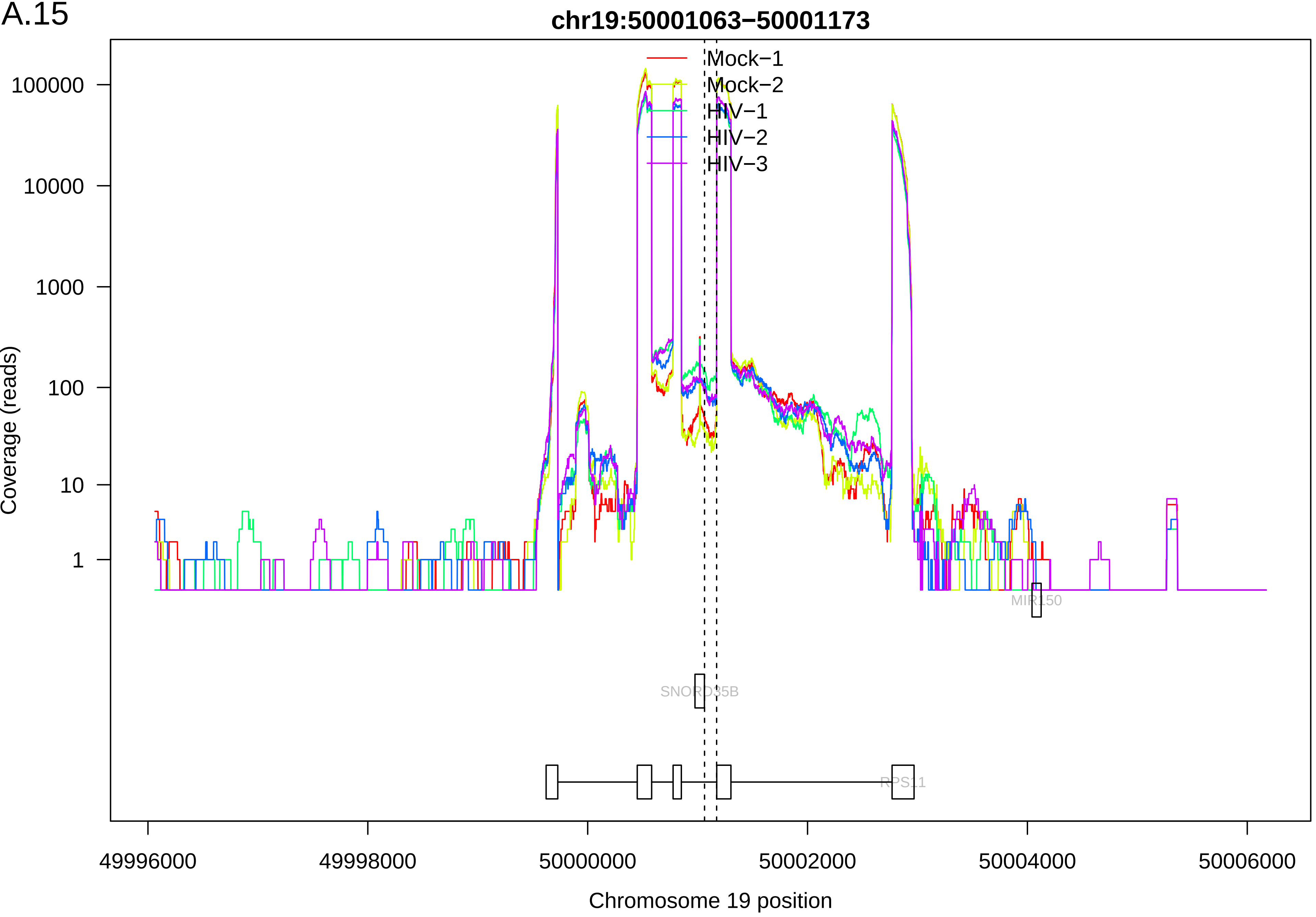

chr19:50001063−50001173
Mock−1
Mock−2
HIV−1
HIV−2
HIV−3
100000
10000
1000
100
10
1
Coverage (reads)
MIR150
SNORD35B
RPS11
49996000
49998000
50000000
50002000
50004000
50006000
Chromosome 19 position

A.16

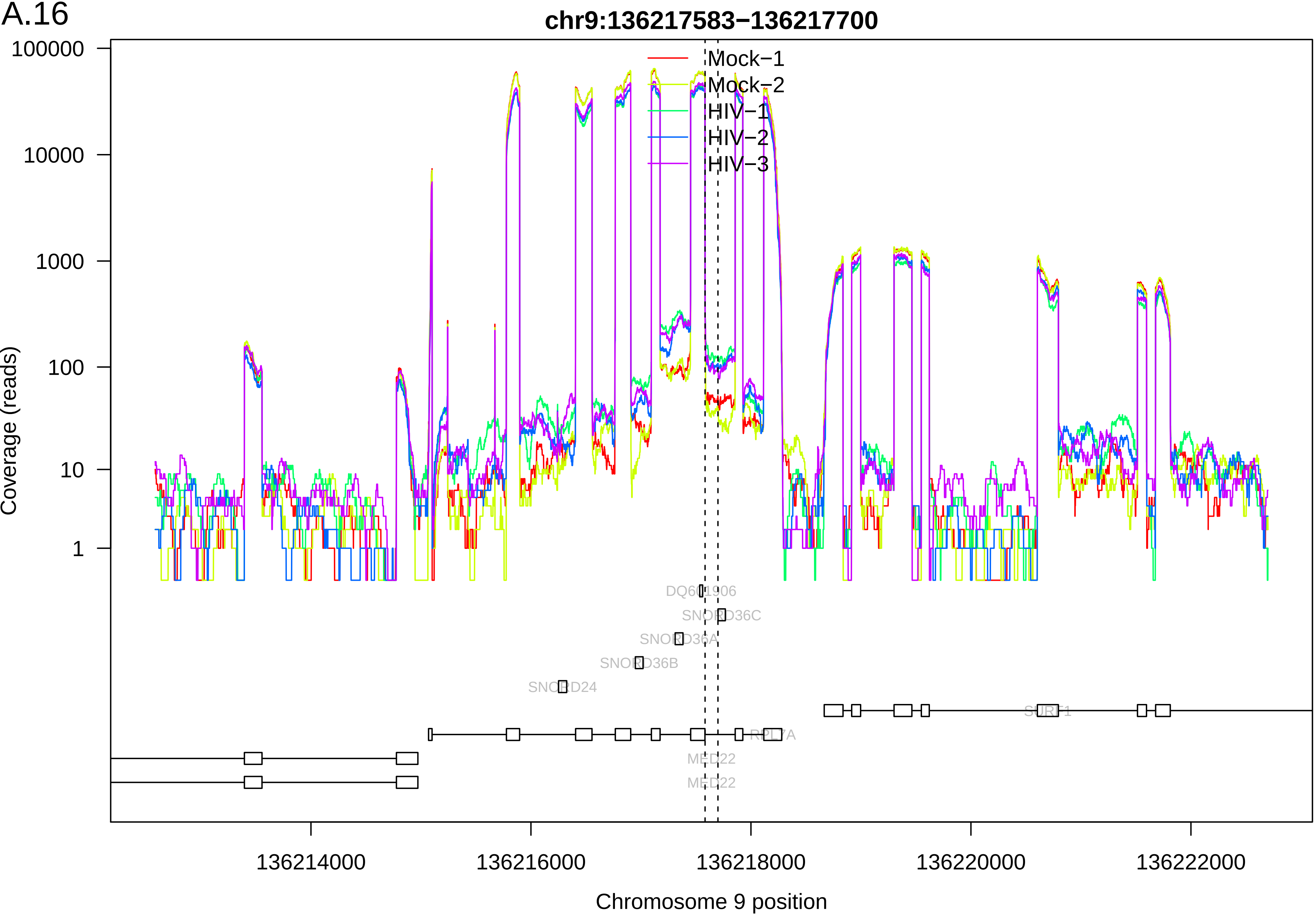
chr9:136217583−136217700
Mock−1
Mock−2
HIV−1
HIV−2
HIV−3
100000
10000
1000
100
10
1
Coverage (reads)
DQ601906
SNORD36C
SNORD36A
SNORD36B
SNORD24
SURF1
RPL7A
MED22
MED22
136214000
136216000
136218000
136220000
136222000
Chromosome 9 position

A.17

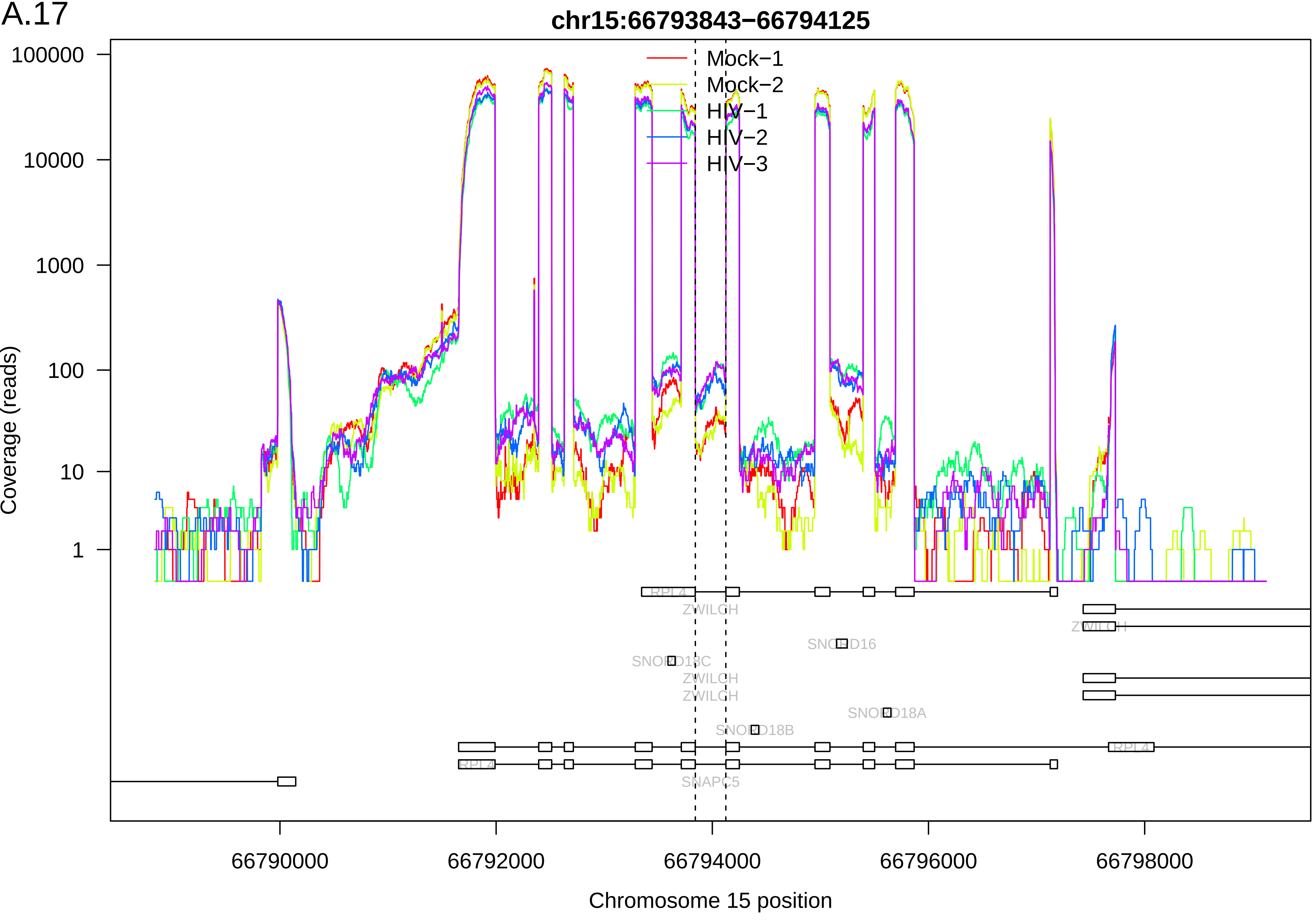

chr15:66793843−66794125
Mock−1
Mock−2
HIV−1
HIV−2
HIV−3
100000
10000
1000
100
10
1
Coverage (reads)
RPL4
ZWILCH
ZWILCH
SNORD16
SNORD18C
ZWILCH
ZWILCH
SNORD18A
SNORD18B
RPL4
RPL4
SNAPC5
66790000
66792000
66794000
66796000
66798000
Chromosome 15 position

A.18

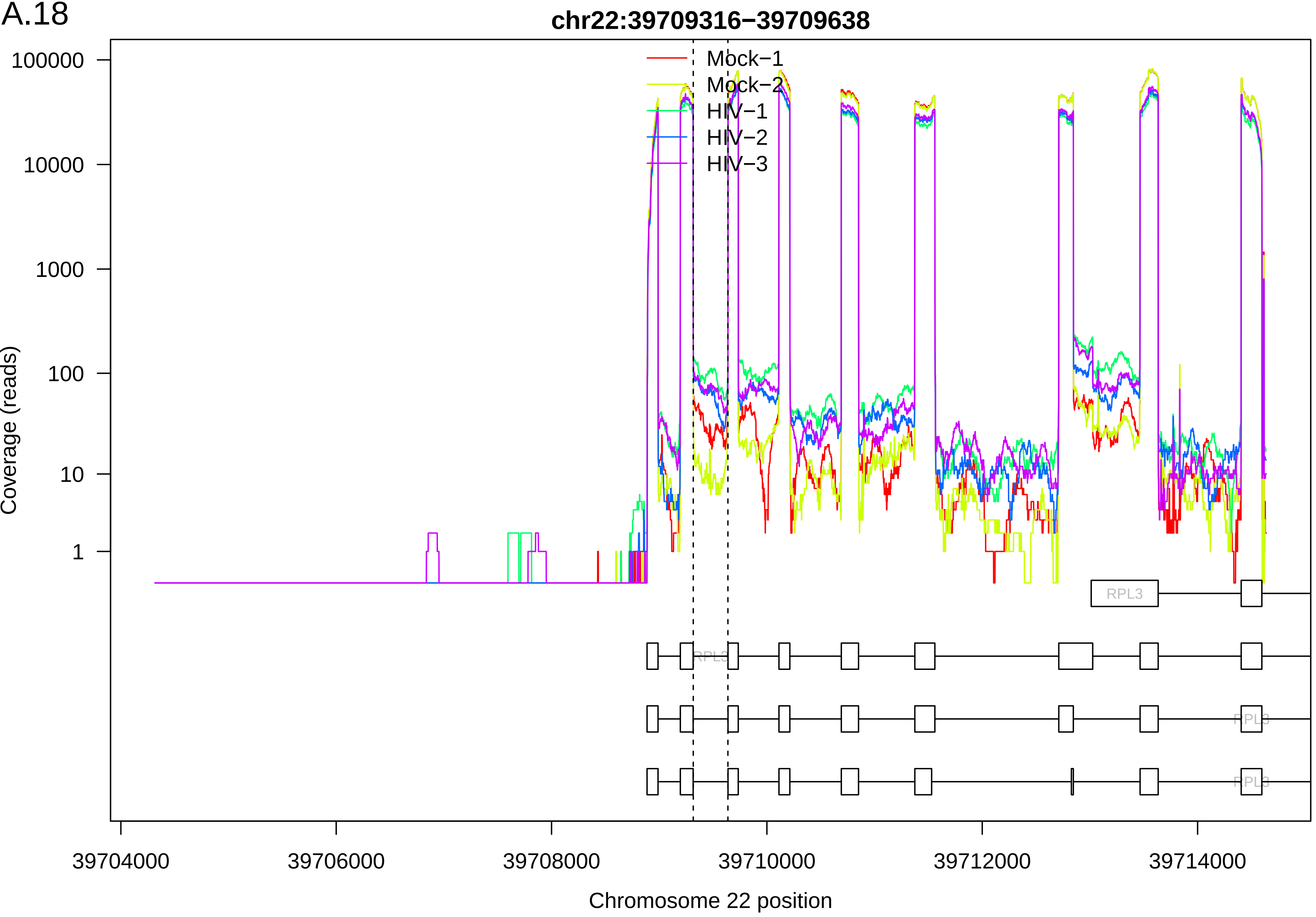
chr22:39709316−39709638
Mock−1
Mock−2
HIV−1
HIV−2
HIV−3
100000
10000
1000
100
10
1
Coverage (reads)
RPL3
RPL3
RPL3
RPL3
39704000
39706000
39708000
39710000
39712000
39714000
Chromosome 22 position

A.19

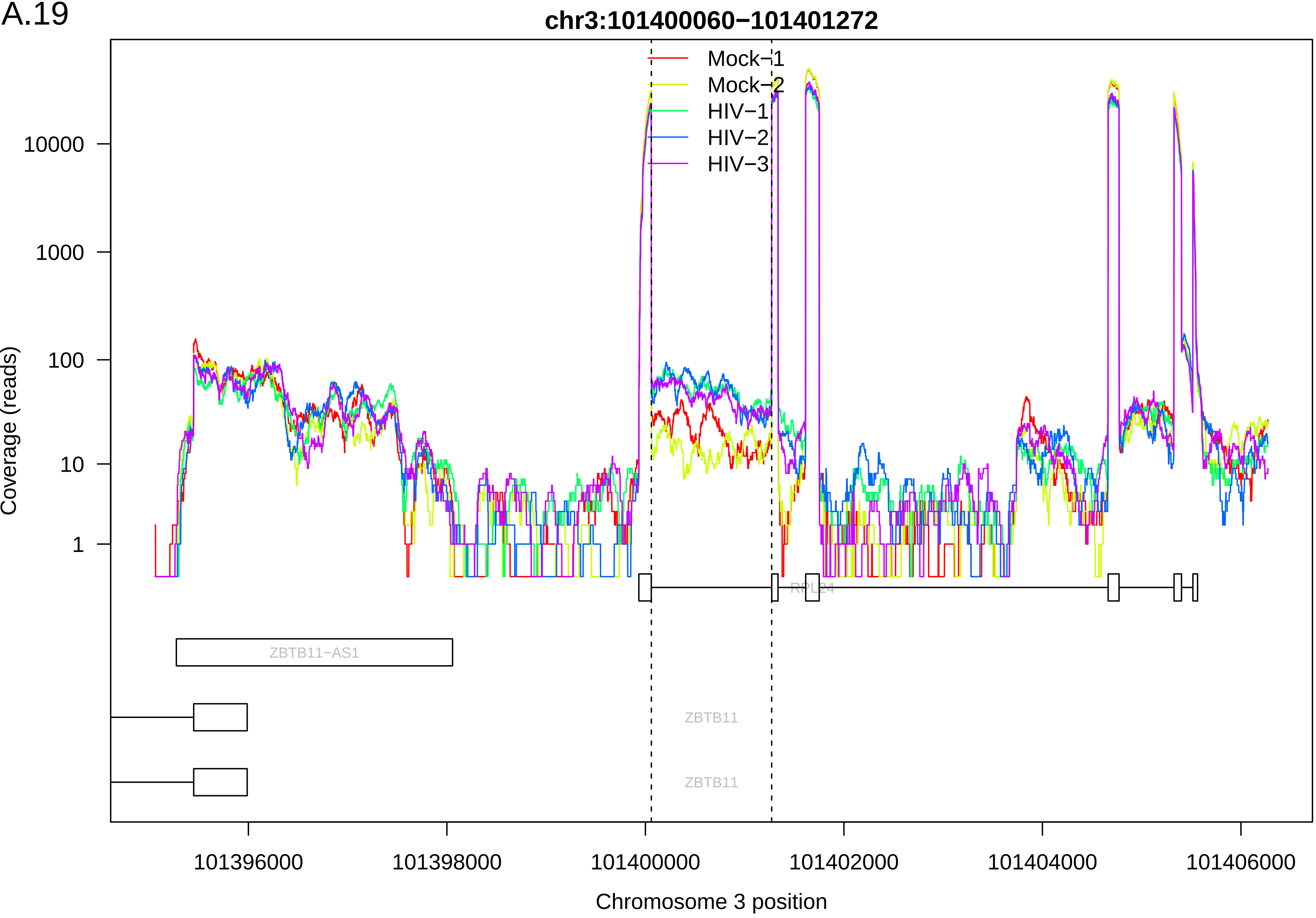
chr3:101400060−101401272
Mock−1
Mock−2
HIV−1
HIV−2
HIV−3
10000
1000
100
10
1
Coverage (reads)
RPL24
ZBTB11−AS1
ZBTB11
ZBTB11
101396000
101398000
101400000
101402000
101404000
101406000
Chromosome 3 position

A.20

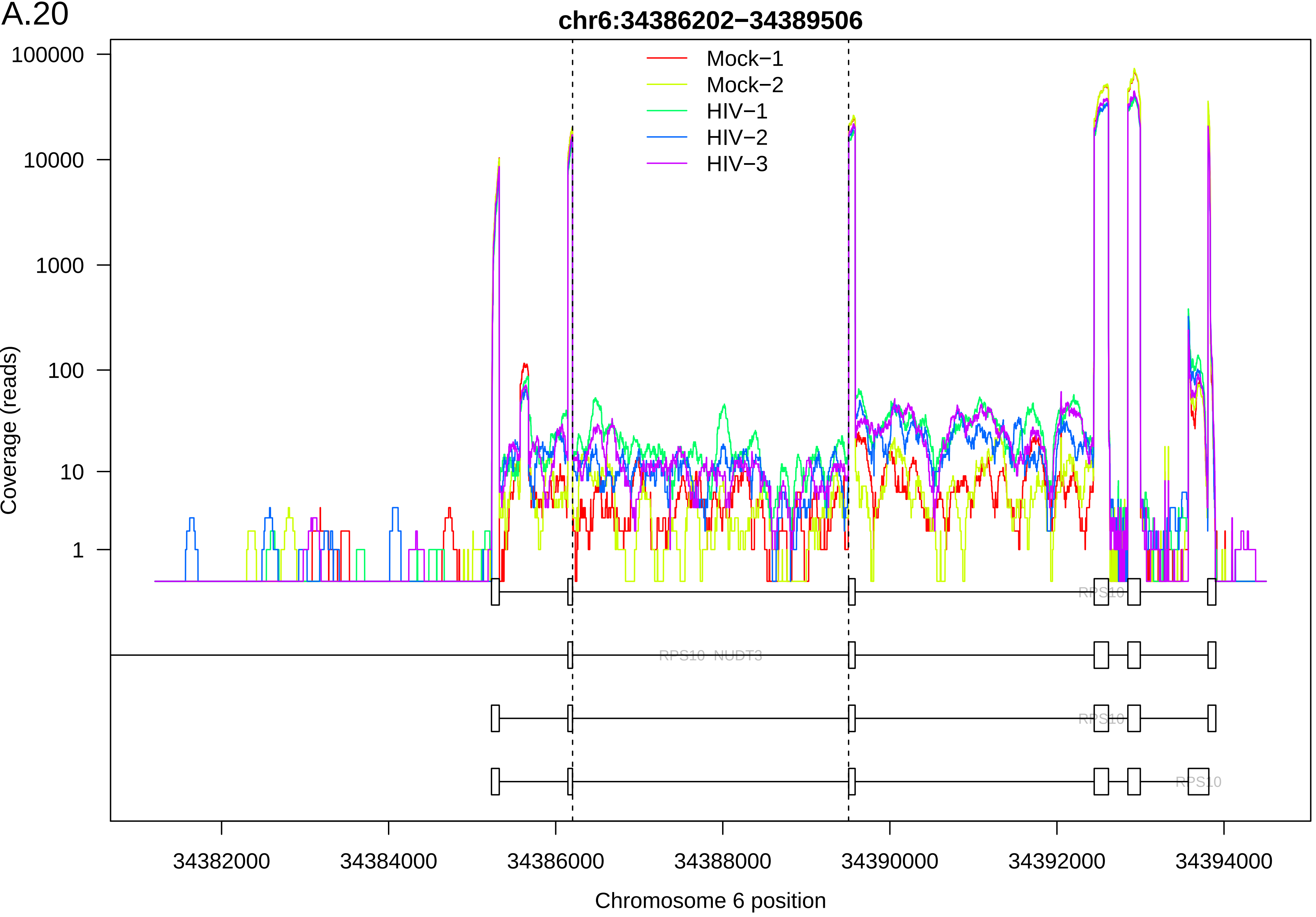
chr6:34386202−34389506
Mock−1
Mock−2
HIV−1
HIV−2
HIV−3
Coverage (reads)
100000
10000
1000
100
10
1
RPS10
RPS10_NUDT3
RPS10
RPS10
34382000
34384000
34386000
34388000
34390000
34392000
34394000
Chromosome 6 position

A.21

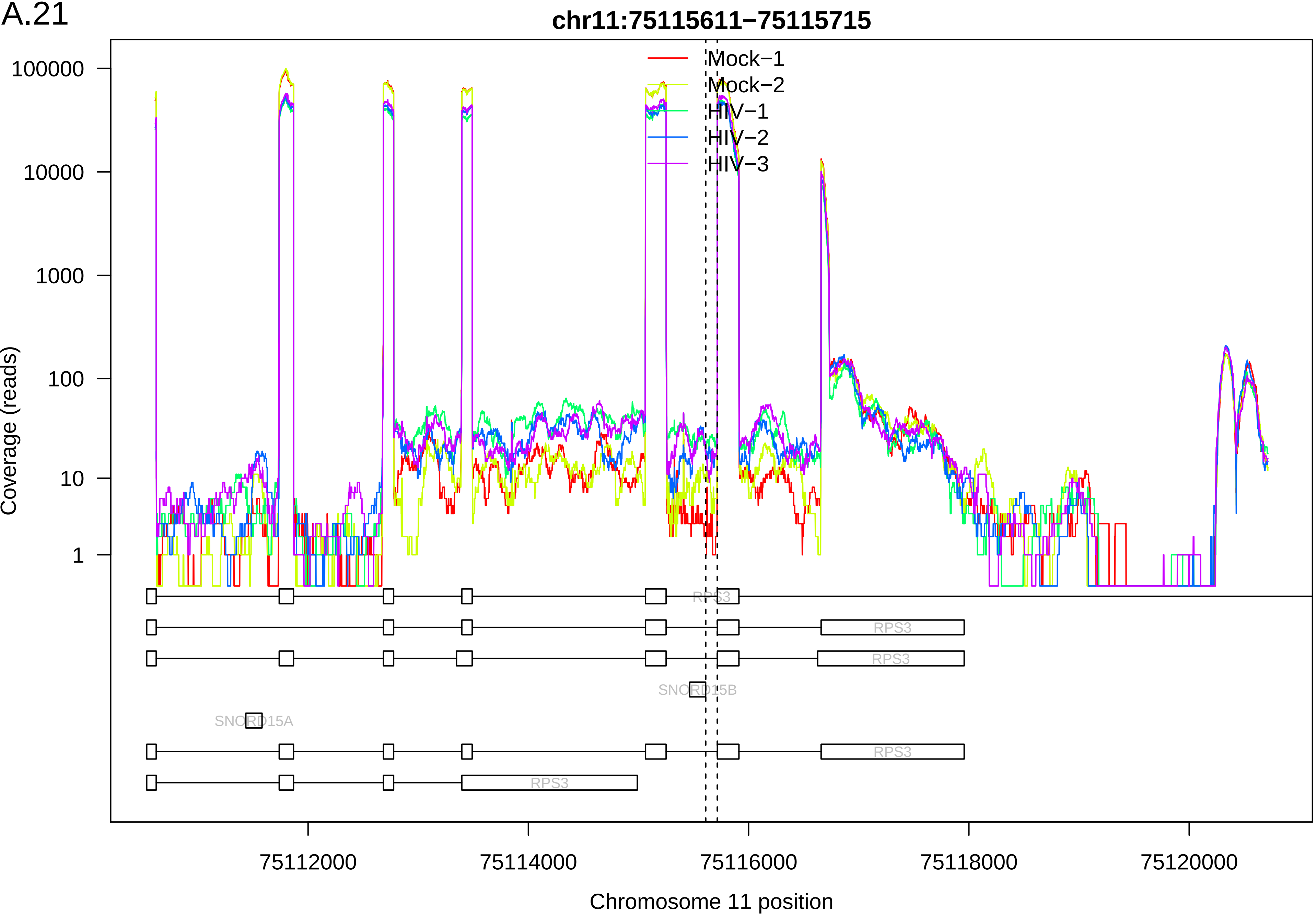
chr11:75115611−75115715
Mock−1
Mock−2
HIV−1
HIV−2
HIV−3
100000
10000
1000
100
10
1
Coverage (reads)
RPS3
RPS3
RPS3
SNORD15B
SNORD15A
RPS3
RPS3
75112000
75114000
75116000
75118000
75120000
Chromosome 11 position

A.22

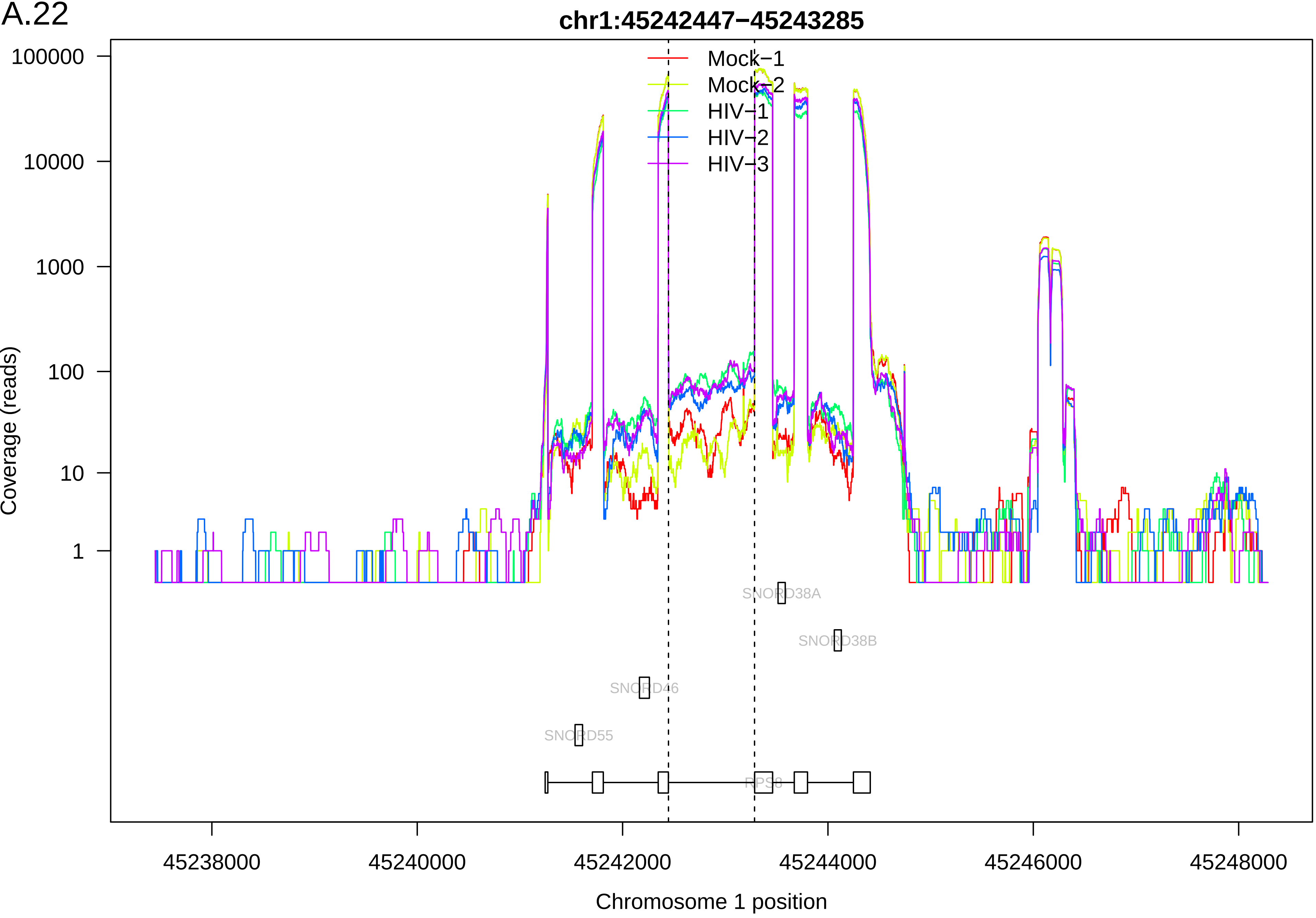
chr1:45242447−45243285
Mock−1
Mock−2
HIV−1
HIV−2
HIV−3
Coverage (reads)
100000
10000
1000
100
10
1
SNORD38A
SNORD38B
SNORD46
SNORD55
RPS8
45238000
45240000
45242000
45244000
45246000
45248000
Chromosome 1 position

A.23

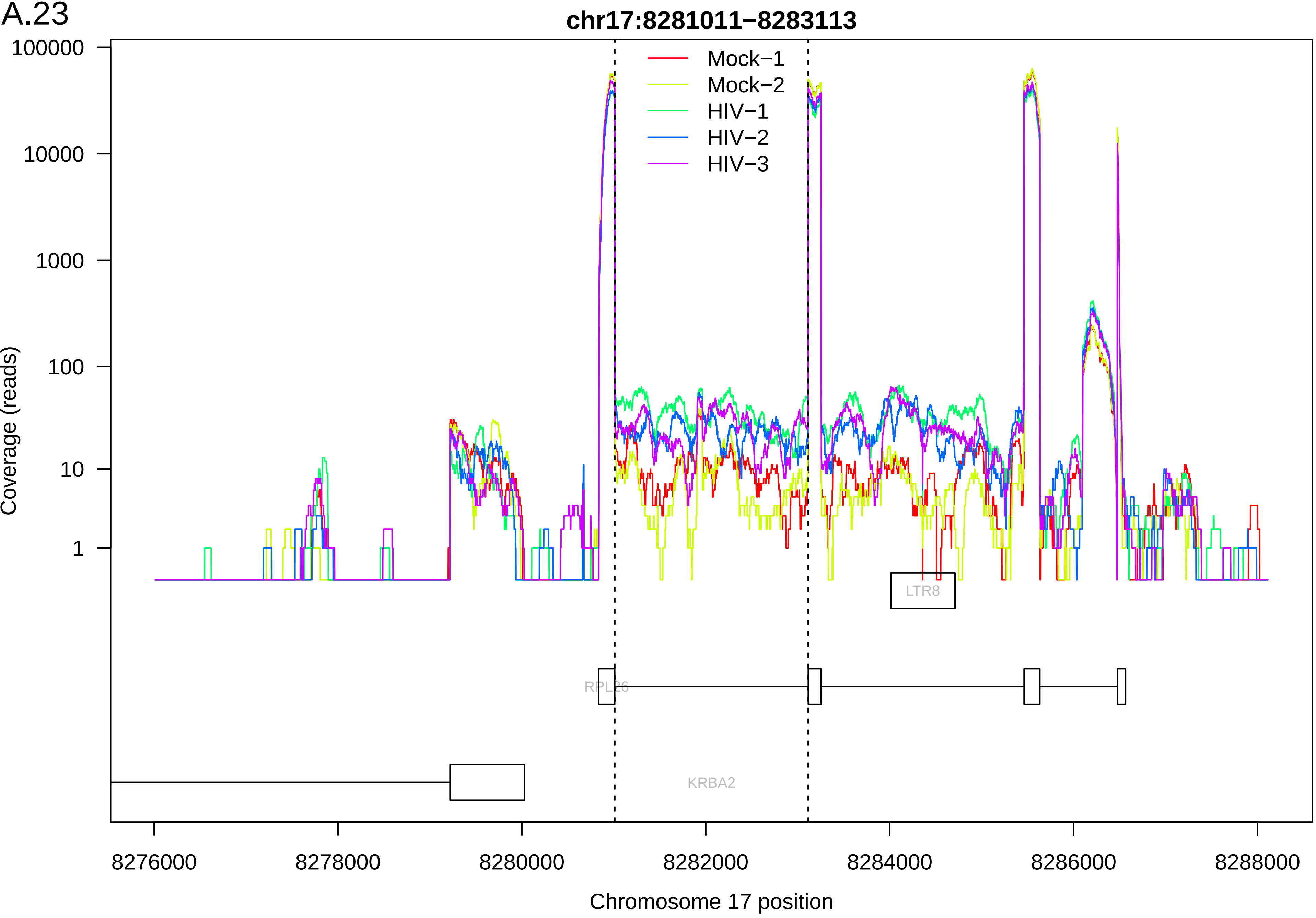

chr17:8281011−8283113
Mock−1
Mock−2
HIV−1
HIV−2
HIV−3
100000
10000
1000
100
10
1
Coverage (reads)
LTR8
RPL26
KRBA2
8276000
8278000
8280000
8282000
8284000
8286000
8288000
Chromosome 17 position

A.24

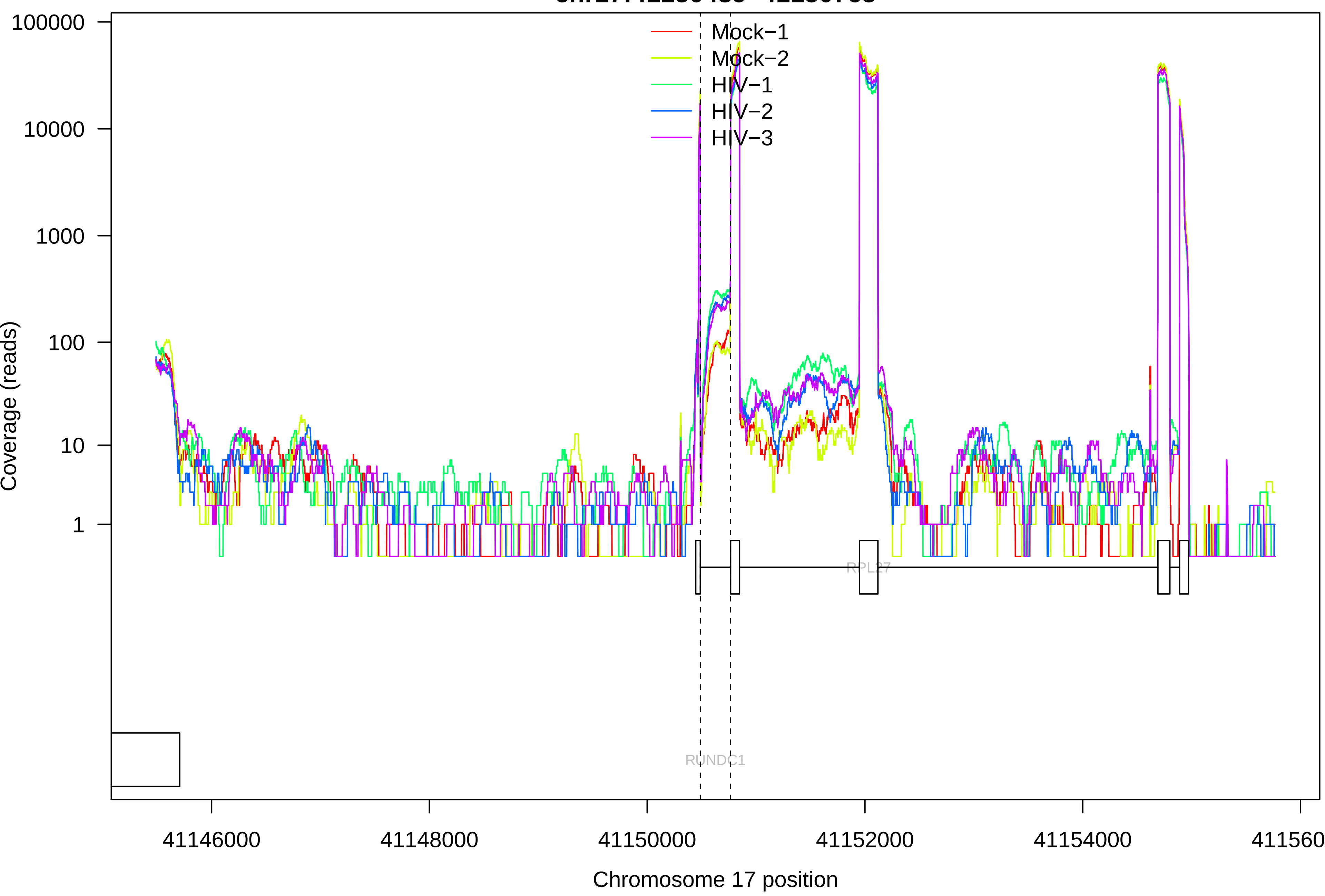
chr17:41150489−41150765
Mock−1
Mock−2
HIV−1
HIV−2
HIV−3
100000
10000
1000
100
10
1
Coverage (reads)
RPL27
RUNDC1
41146000
41148000
41150000
41152000
41154000
411560
Chromosome 17 position

A.25

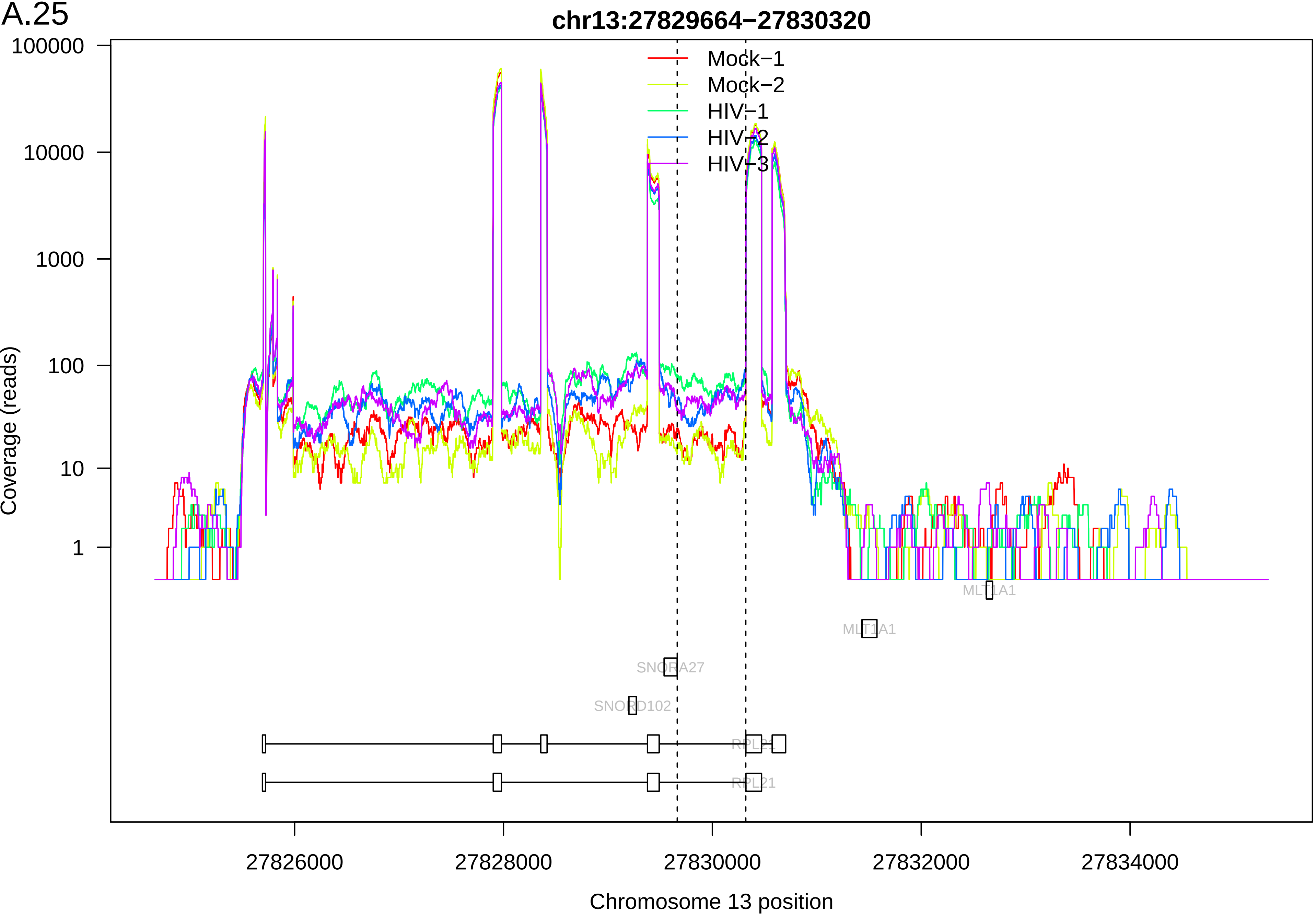
chr13:27829664−27830320
Mock−1
Mock−2
HIV−1
HIV−2
HIV−3
Coverage (reads)
100000
10000
1000
100
10
1
MLT1A1
MLT1A1
SNORA27
SNORD102
RPL21
RPL21
27826000
27828000
27830000
27832000
27834000
Chromosome 13 position

A.26

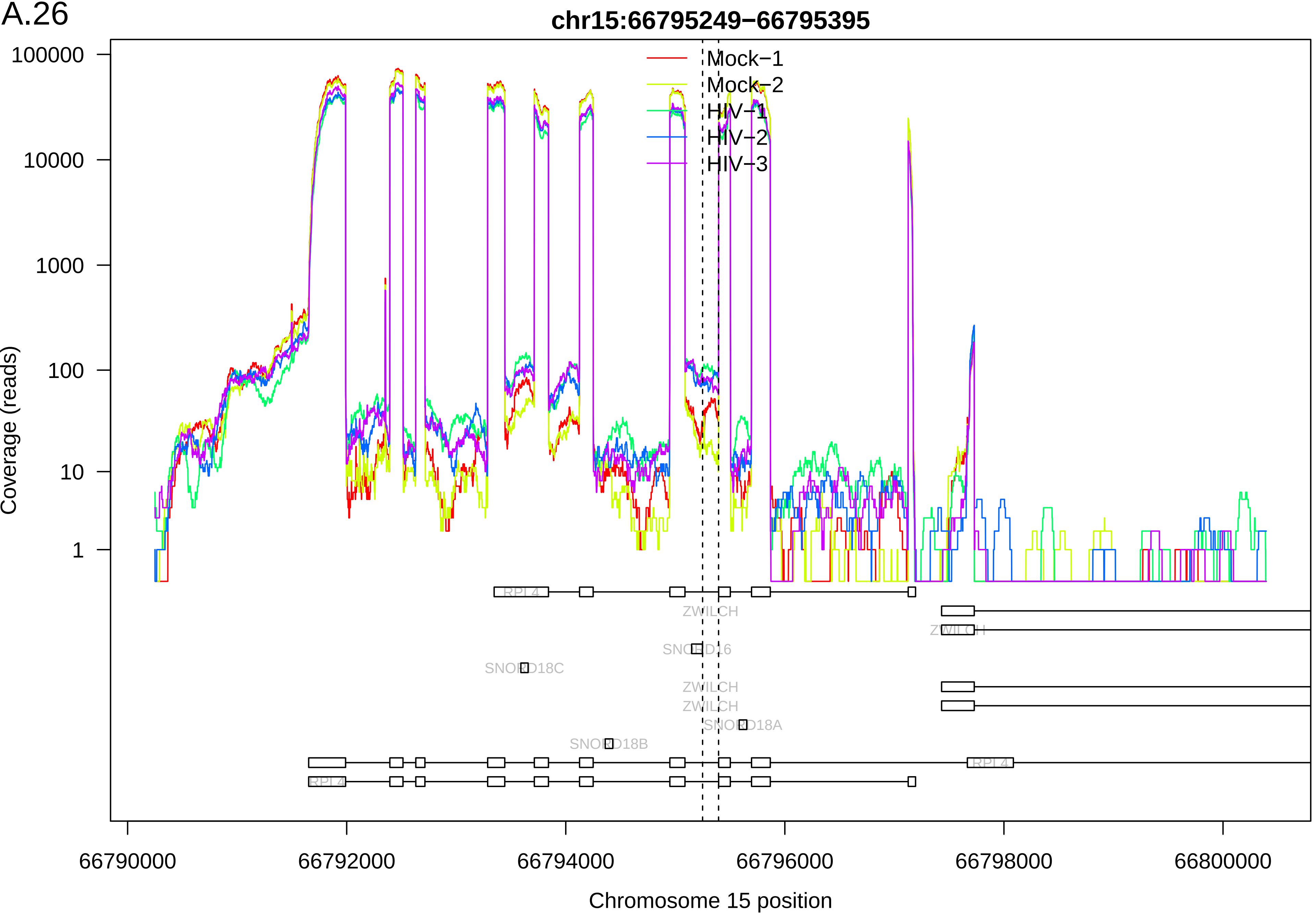

chr15:66795249−66795395
Mock−1
Mock−2
HIV−1
HIV−2
HIV−3
Coverage (reads)
100000
10000
1000
100
10
1
RPL4
ZWILCH
ZWILCH
SNORD16
SNORD18C
ZWILCH
ZWILCH
SNORD18A
SNORD18B
RPL4
RPL4
66790000
66792000
66794000
66796000
66798000
66800000
Chromosome 15 position

A.27

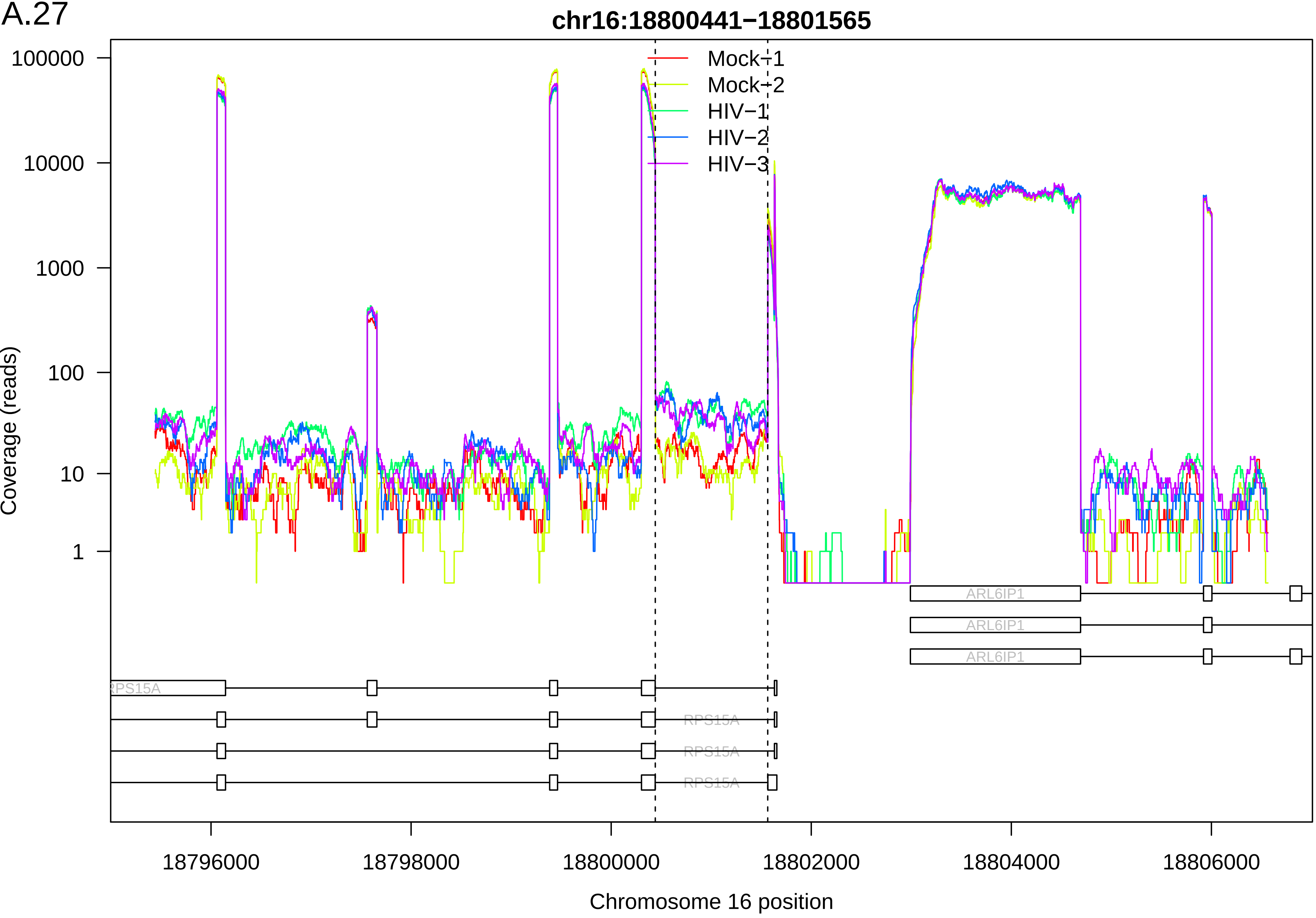

chr16:18800441−18801565
Mock−1
Mock−2
HIV−1
HIV−2
HIV−3
100000
10000
1000
100
10
1
Coverage (reads)
ARL6IP1
ARL6IP1
ARL6IP1
RPS15A
RPS15A
RPS15A
RPS15A
18796000
18798000
18800000
18802000
18804000
18806000
Chromosome 16 position

A.28

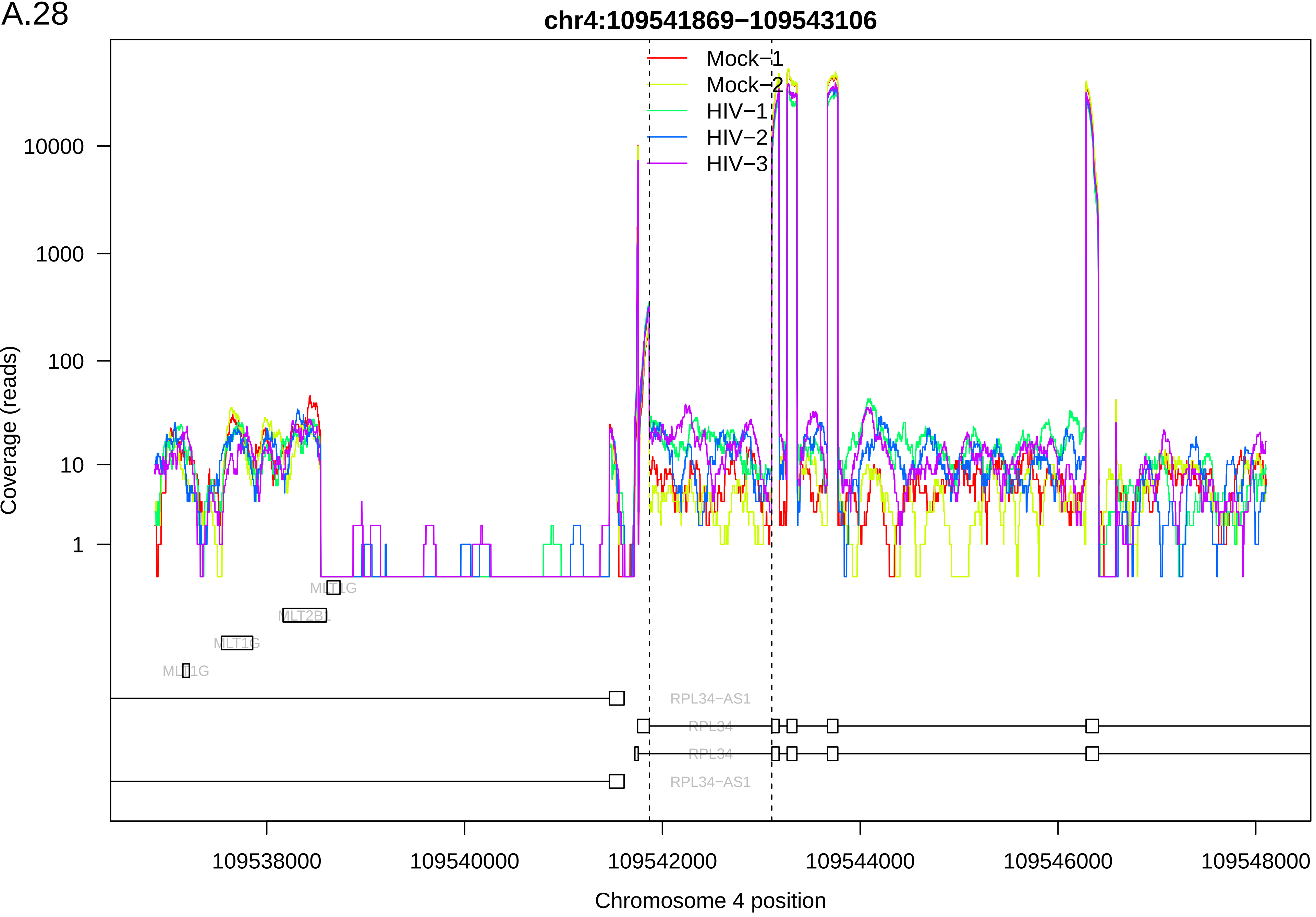

chr4:109541869−109543106
Mock−1
Mock−2
HIV−1
HIV−2
HIV−3
Coverage (reads)
10000
1000
100
10
1
MLT1G
MLT2B1
MLT1G
MLT1G
RPL34−AS1
RPL34
RPL34
RPL34−AS1
109538000
109540000
109542000
109544000
109546000
109548000
Chromosome 4 position

A.29

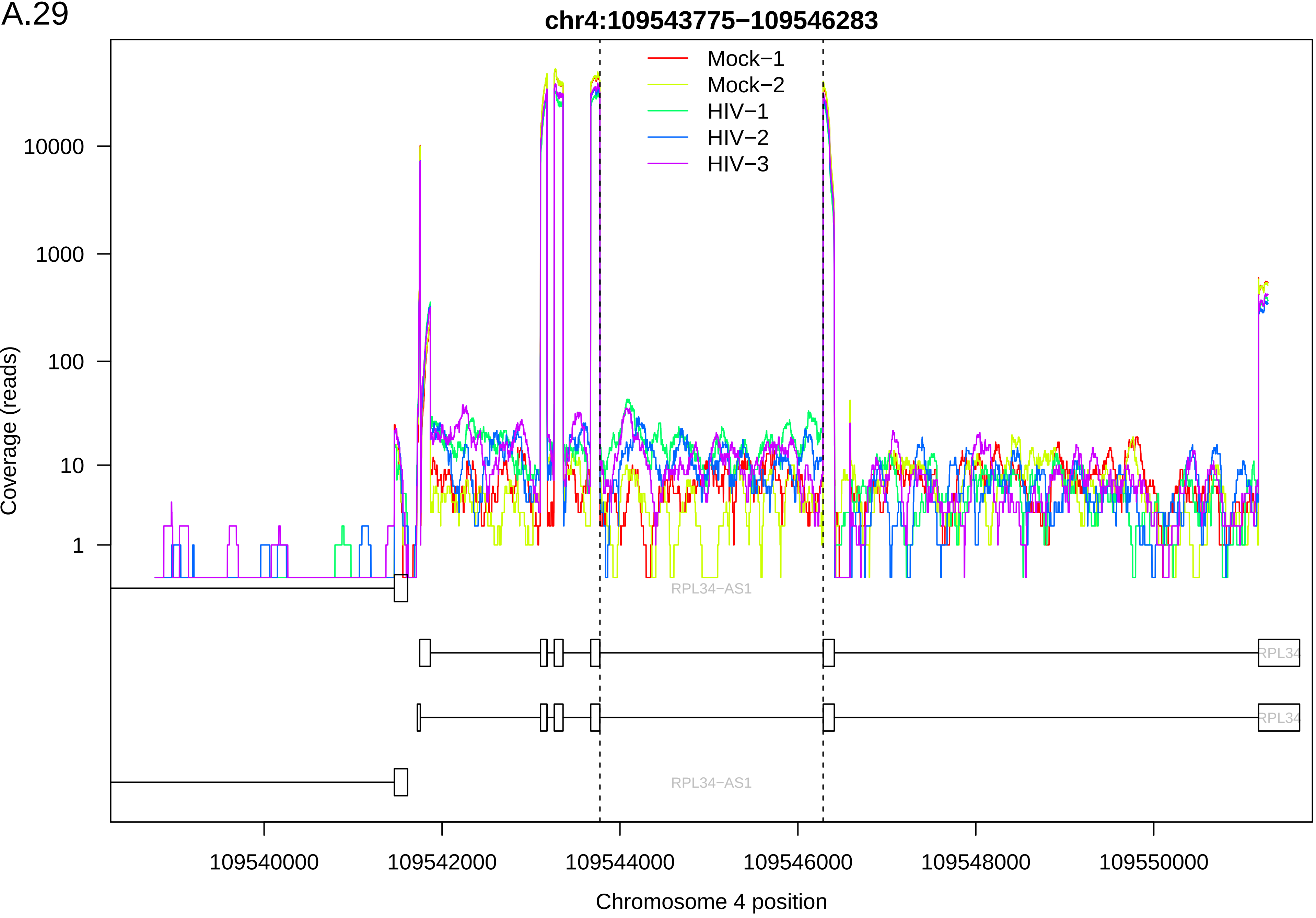
chr4:109543775−109546283
Mock−1
Mock−2
HIV−1
HIV−2
HIV−3
Coverage (reads)
10000
1000
100
10
1
RPL34−AS1
RPL34
RPL34
RPL34−AS1
109540000
109542000
109544000
109546000
109548000
109550000
Chromosome 4 position

A.30

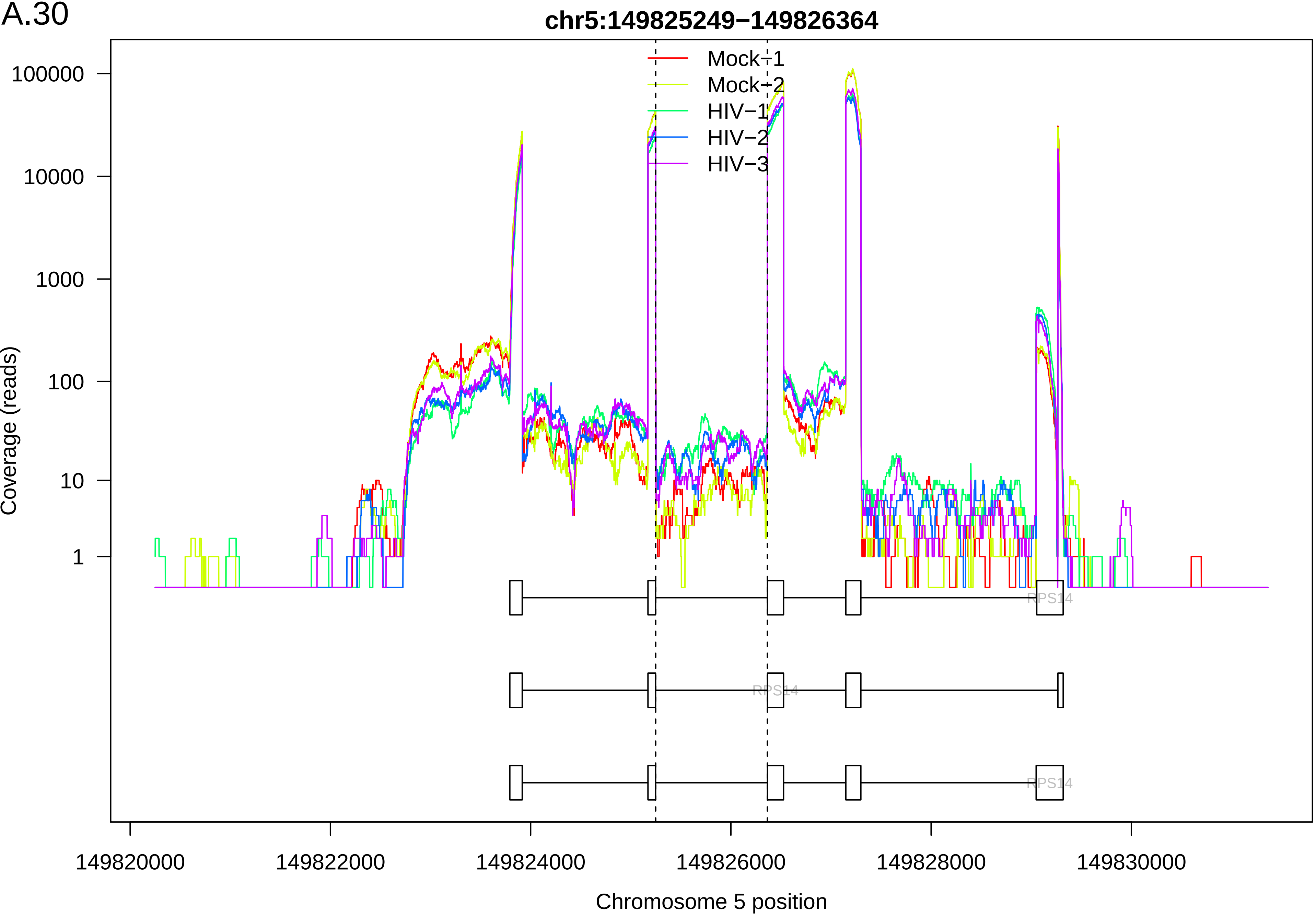
chr5:149825249−149826364
Mock−1
Mock−2
HIV−1
HIV−2
HIV−3
100000
10000
1000
100
10
1
Coverage (reads)
RPS14
RPS14
RPS14
149820000
149822000
149824000
149826000
149828000
149830000
Chromosome 5 position

A.31

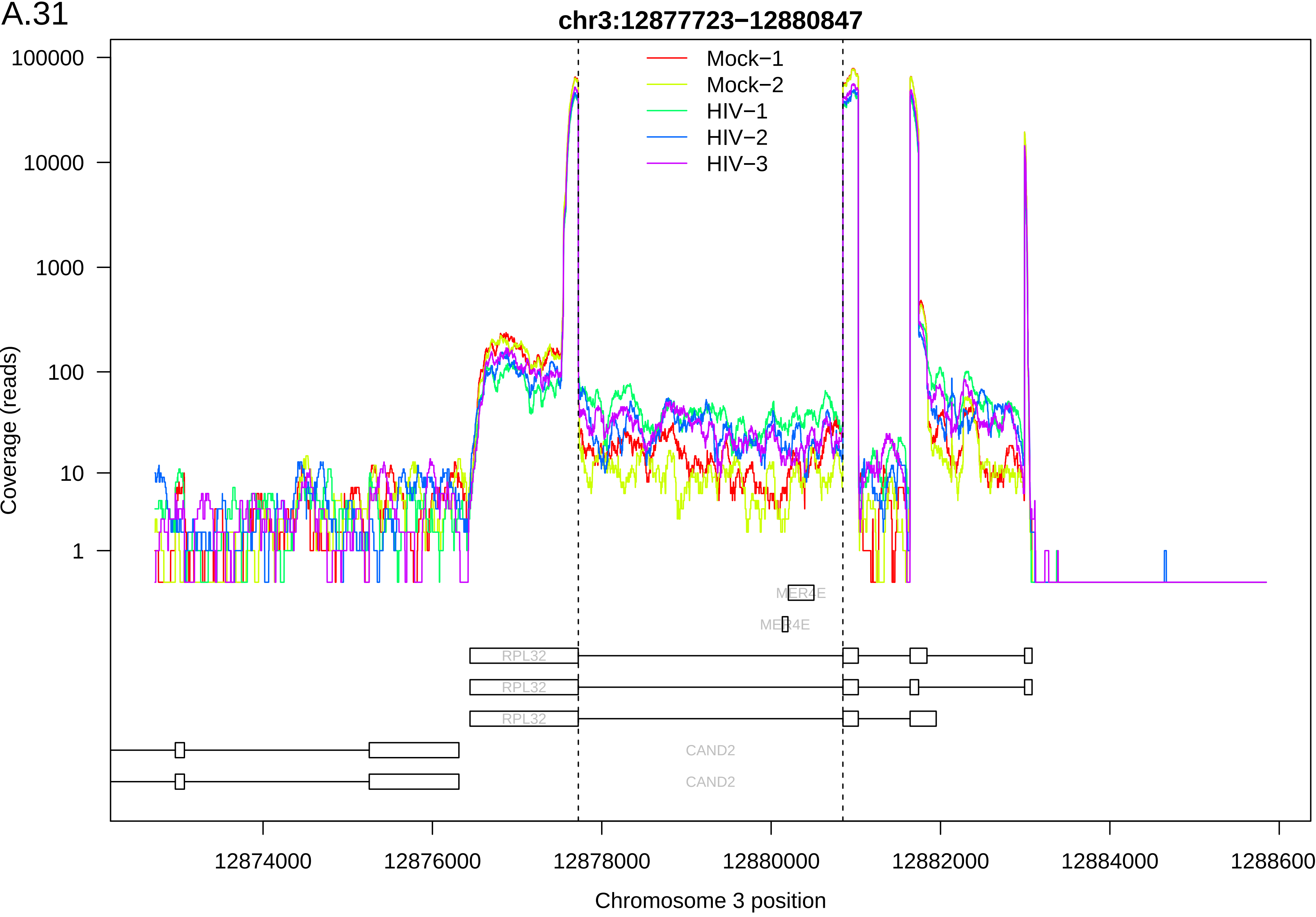
chr3:12877723−12880847
Mock−1
Mock−2
HIV−1
HIV−2
HIV−3
100000
10000
1000
100
10
1
Coverage (reads)
MER4E
MER4E
RPL32
RPL32
RPL32
CAND2
CAND2
12874000
12876000
12878000
12880000
12882000
12884000
12886000
Chromosome 3 position

A.32

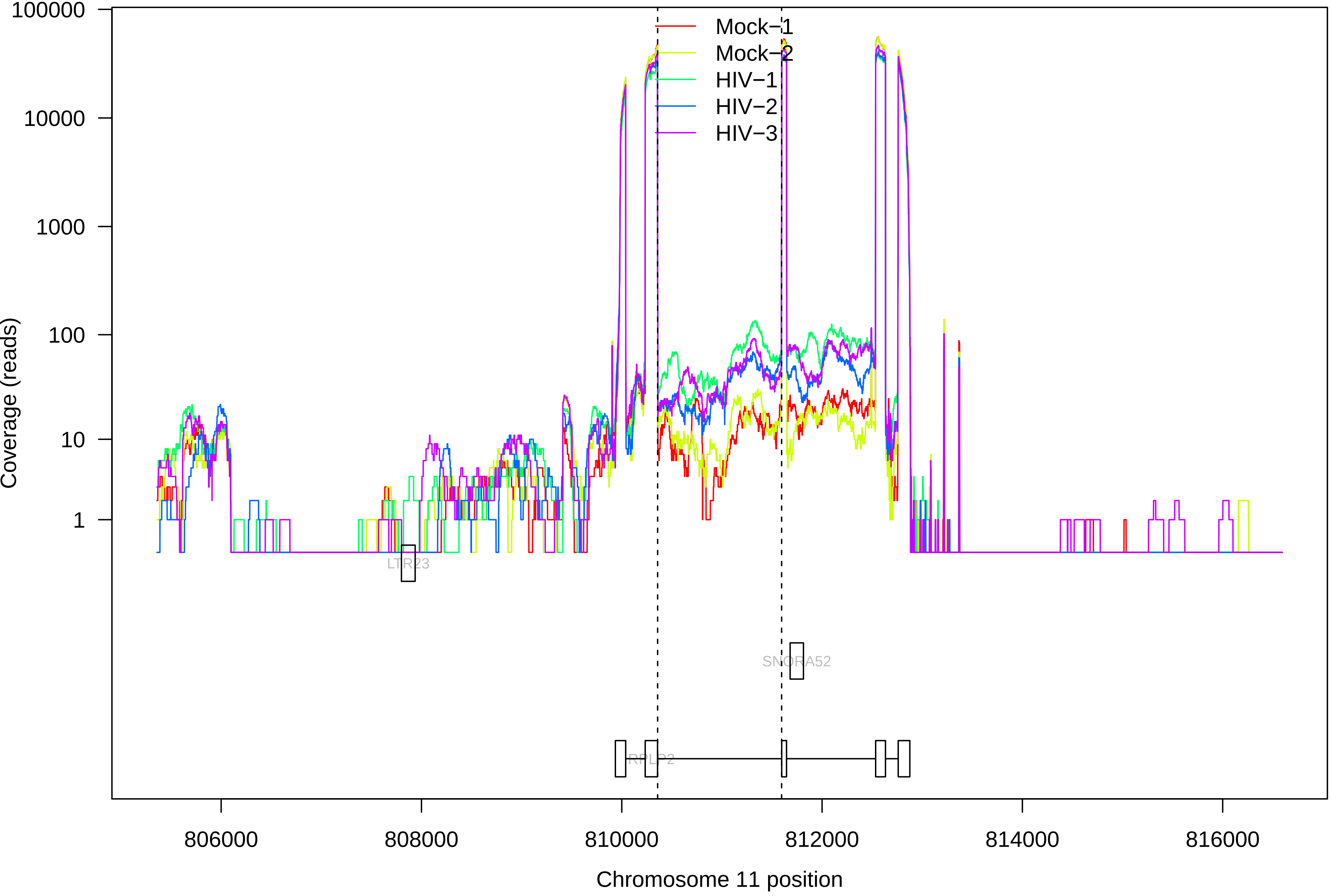
chr11:810358−811596
Mock−1
Mock−2
HIV−1
HIV−2
HIV−3
Coverage (reads)
100000
10000
1000
100
10
1
LTR23
SNORA52
RPLP2
806000
808000
810000
812000
814000
816000
Chromosome 11 position

A.33

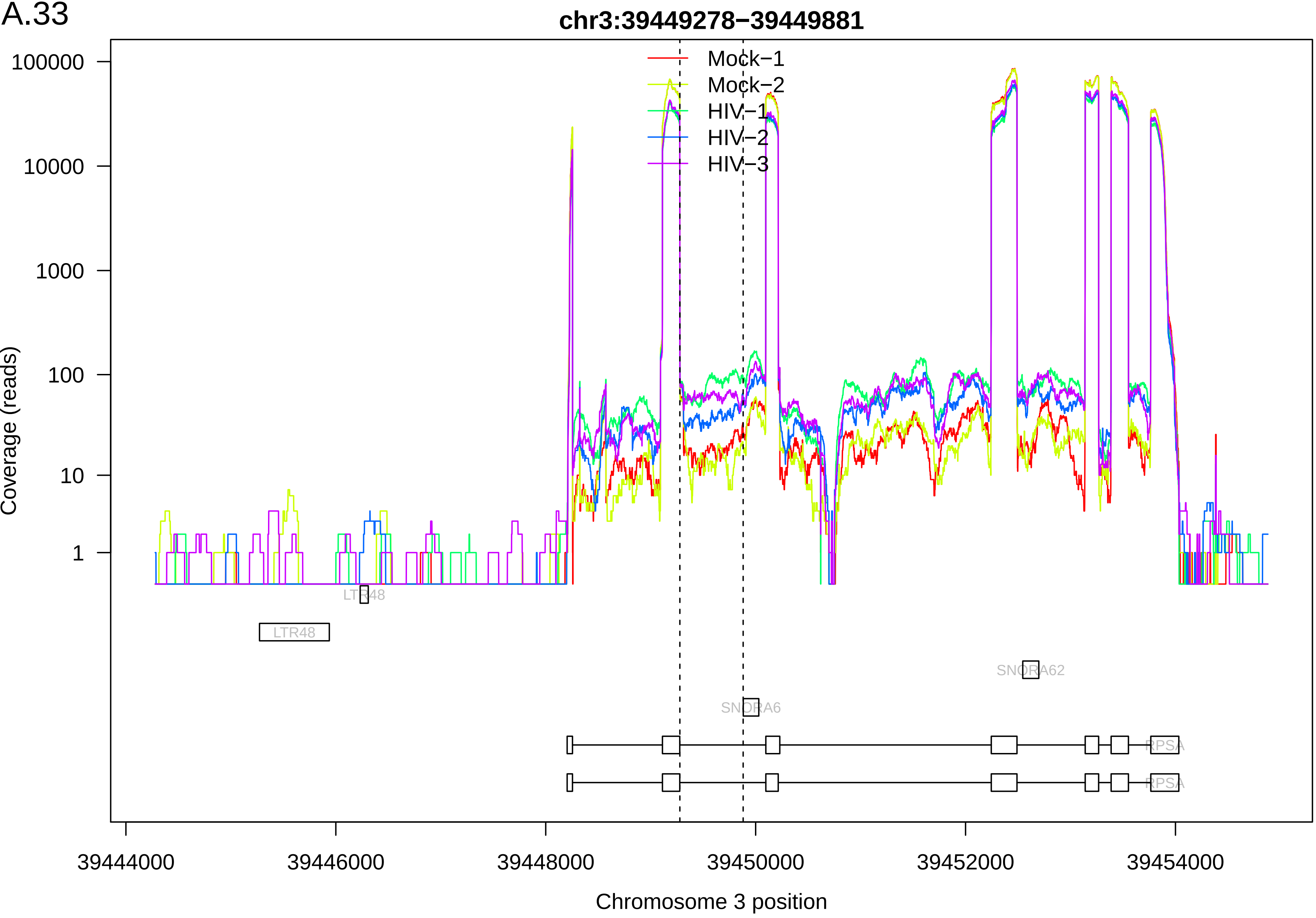

chr3:39449278−39449881
Mock−1
Mock−2
HIV−1
HIV−2
HIV−3
100000
10000
1000
100
10
1
Coverage (reads)
LTR48
LTR48
SNORA62
SNORA6
RPSA
RPSA
39444000
39446000
39448000
39450000
39452000
39454000
Chromosome 3 position

A.34

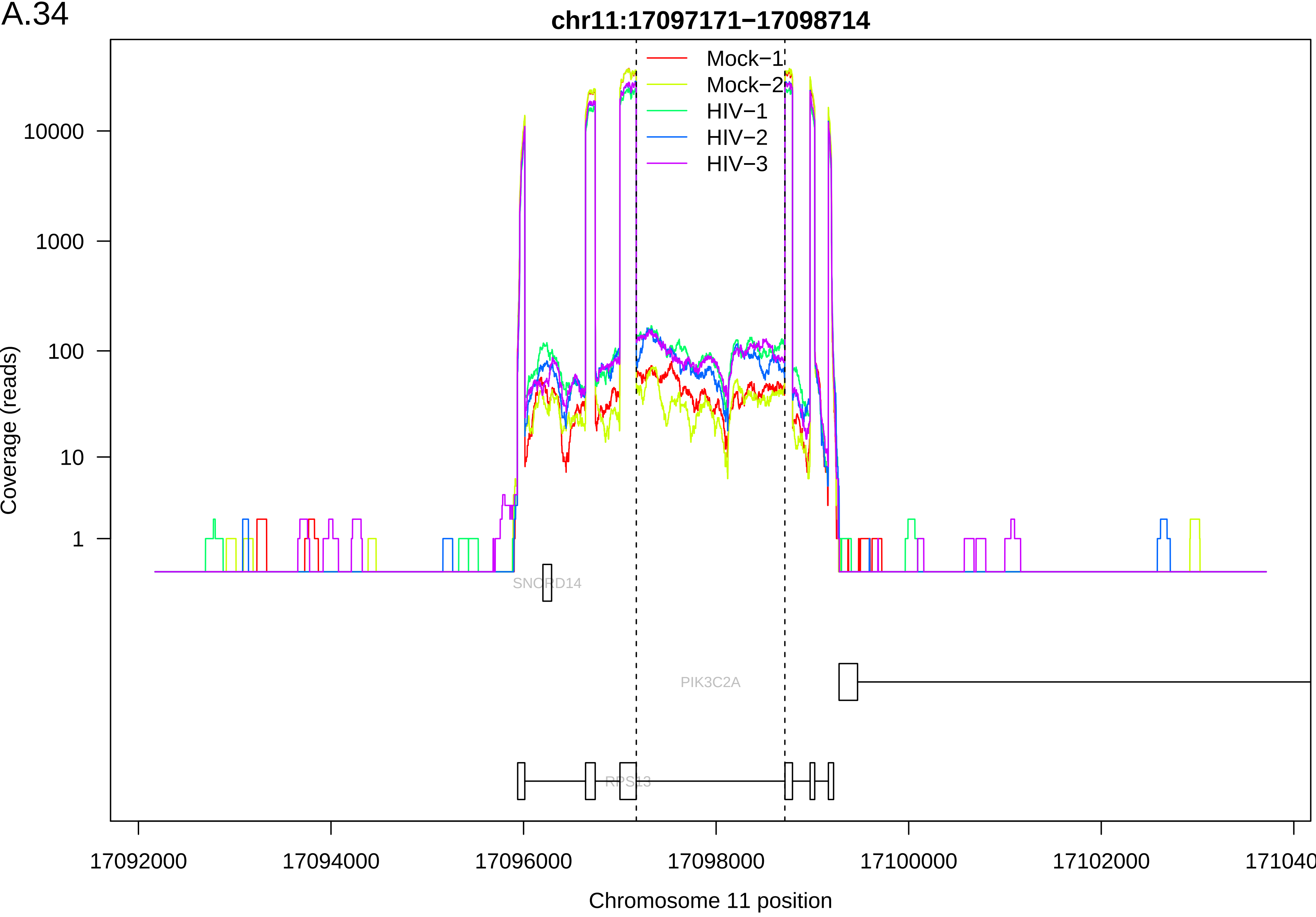
chr11:17097171−17098714
Mock−1
Mock−2
HIV−1
HIV−2
HIV−3
10000
1000
100
10
1
Coverage (reads)
SNORD14
PIK3C2A
RPS13
17092000
17094000
17096000
17098000
17100000
17102000
Chromosome 11 position

A.35

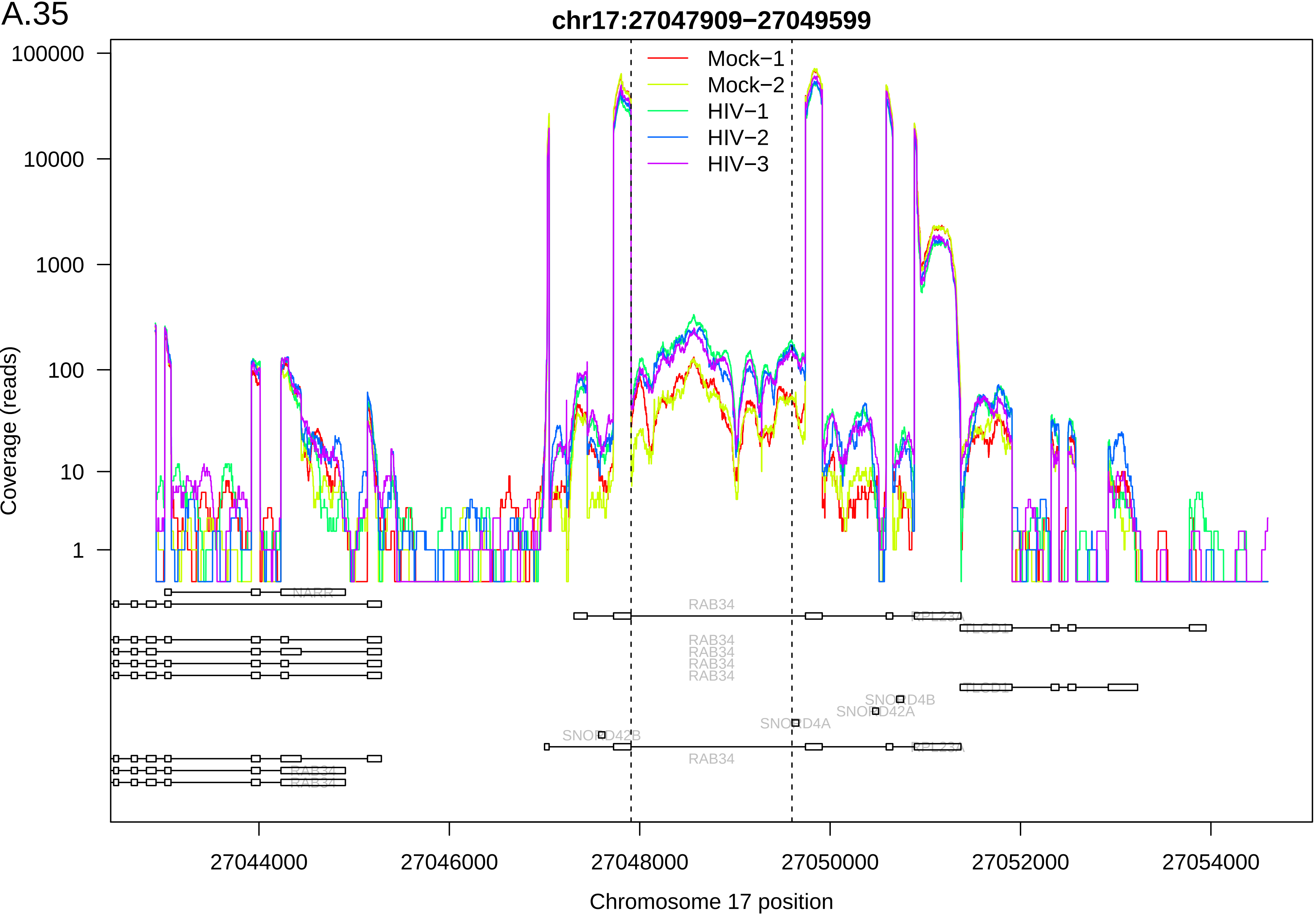
chr17:27047909−27049599
Coverage (reads)
100000
10000
1000
100
10
1
Mock−1
Mock−2
HIV−1
HIV−2
HIV−3
NARR
RAB34
RPL23A
TLCD1
SNORD4B
SNORD42A
SNORD4A
SNORD42B
27044000
27046000
27048000
27050000
27052000
27054000
Chromosome 17 position

A.36

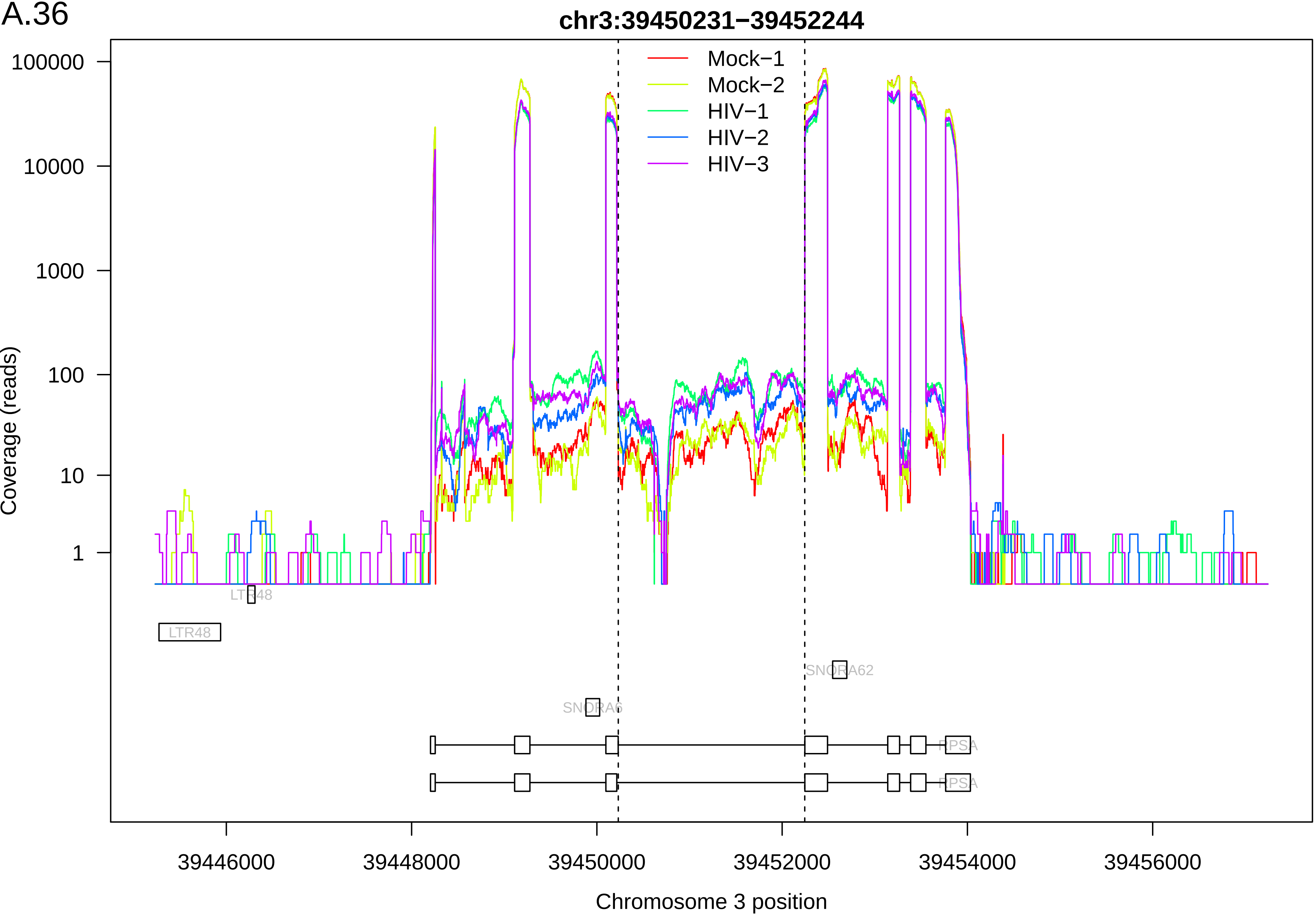
chr3:39450231−39452244
Mock−1
Mock−2
HIV−1
HIV−2
HIV−3
Coverage (reads)
100000
10000
1000
100
10
1
LTR48
LTR48
SNORA62
SNORA6
RPSA
RPSA
39446000
39448000
39450000
39452000
39454000
39456000
Chromosome 3 position

A.37

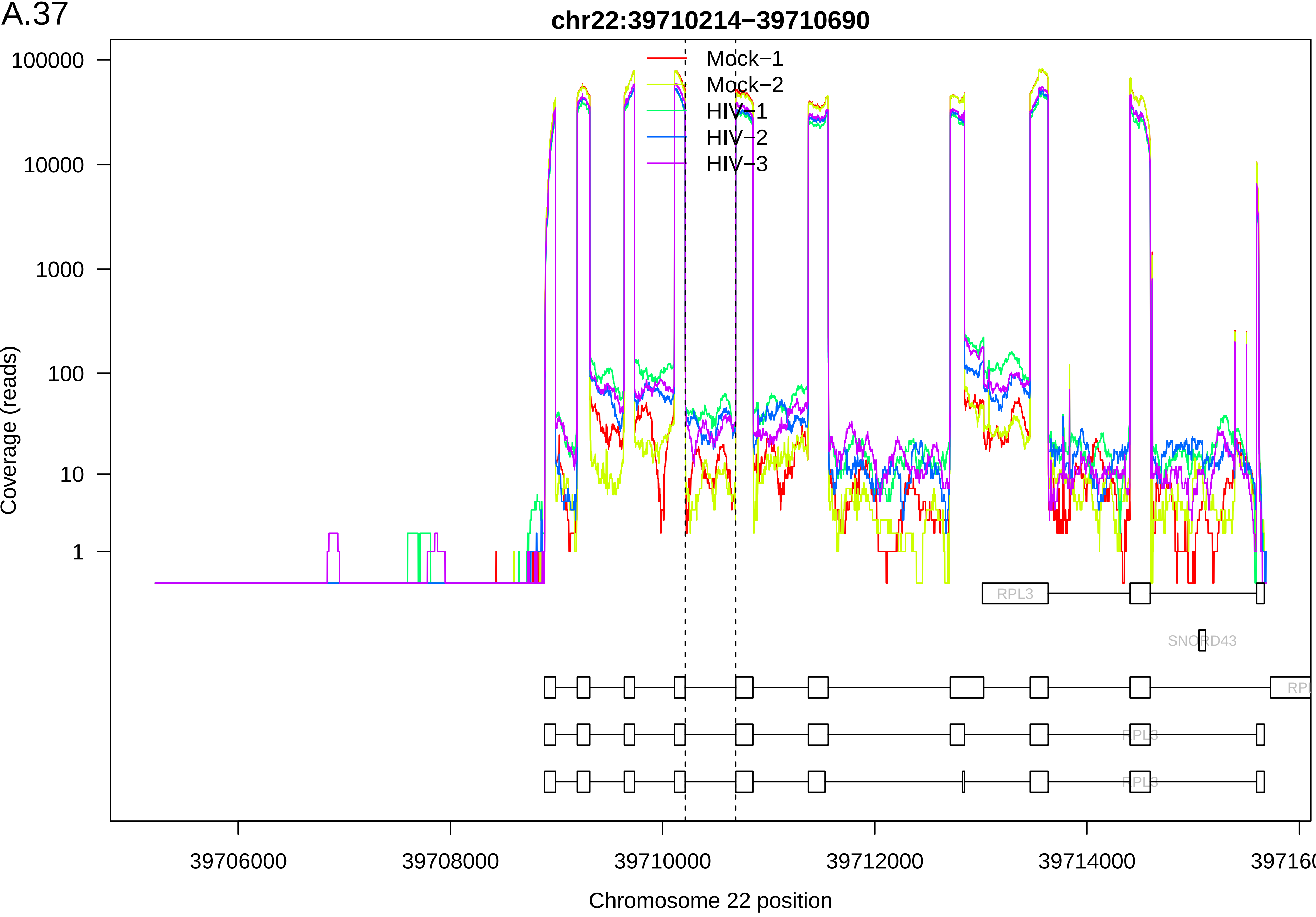

chr22:39710214−39710690
Mock−1
Mock−2
HIV−1
HIV−2
HIV−3
100000
10000
1000
100
10
1
Coverage (reads)
RPL3
SNORD43
RPL3
RPL3
39706000
39708000
39710000
39712000
39714000
Chromosome 22 position

A.38

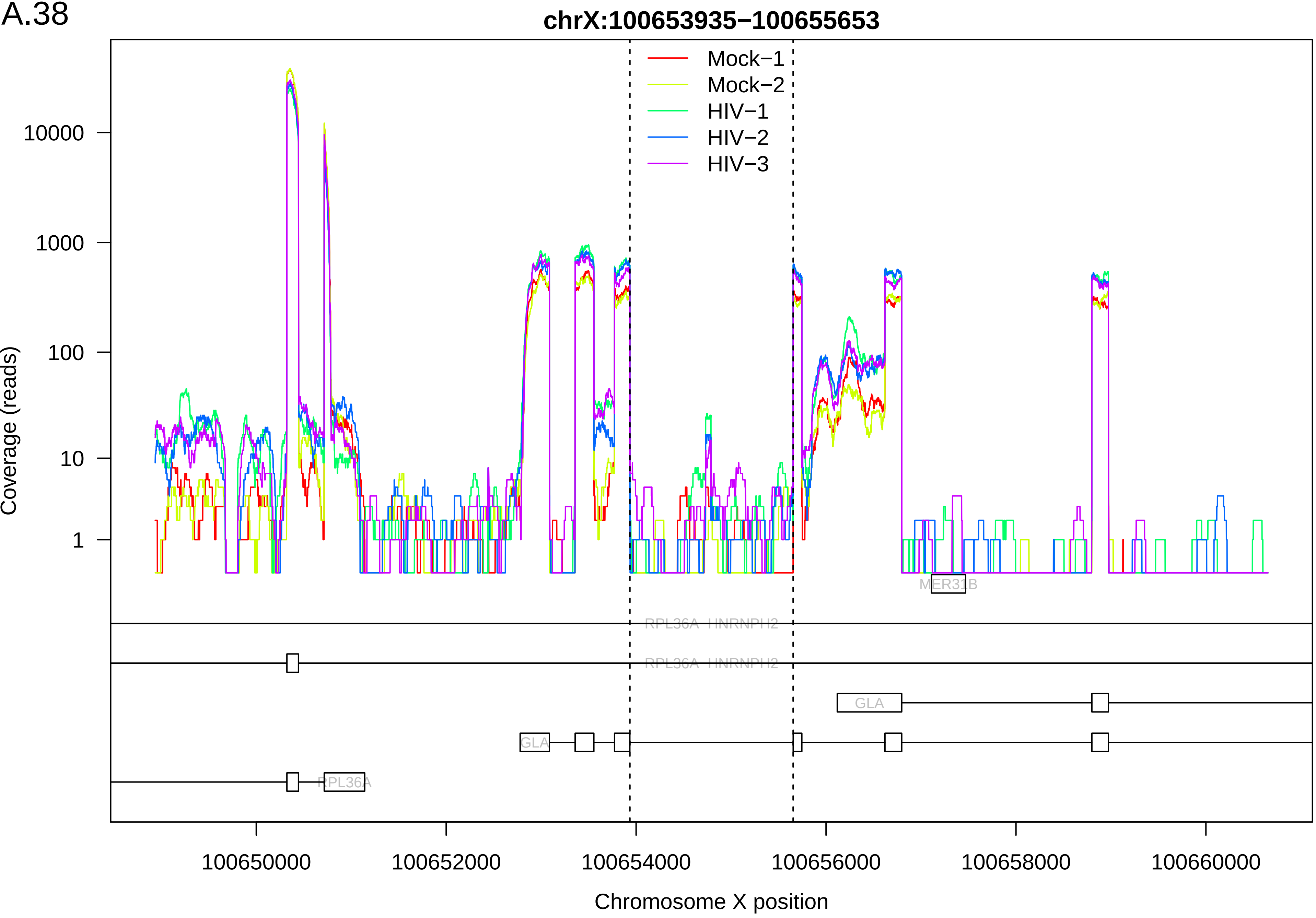
chrX:100653935−100655653
Mock−1
Mock−2
HIV−1
HIV−2
HIV−3
10000
1000
100
10
1
Coverage (reads)
MER31B
RPL36A−HNRNPH2
RPL36A−HNRNPH2
GLA
GLA
RPL36A
100650000
100652000
100654000
100656000
100658000
100660000
Chromosome X position

A.39

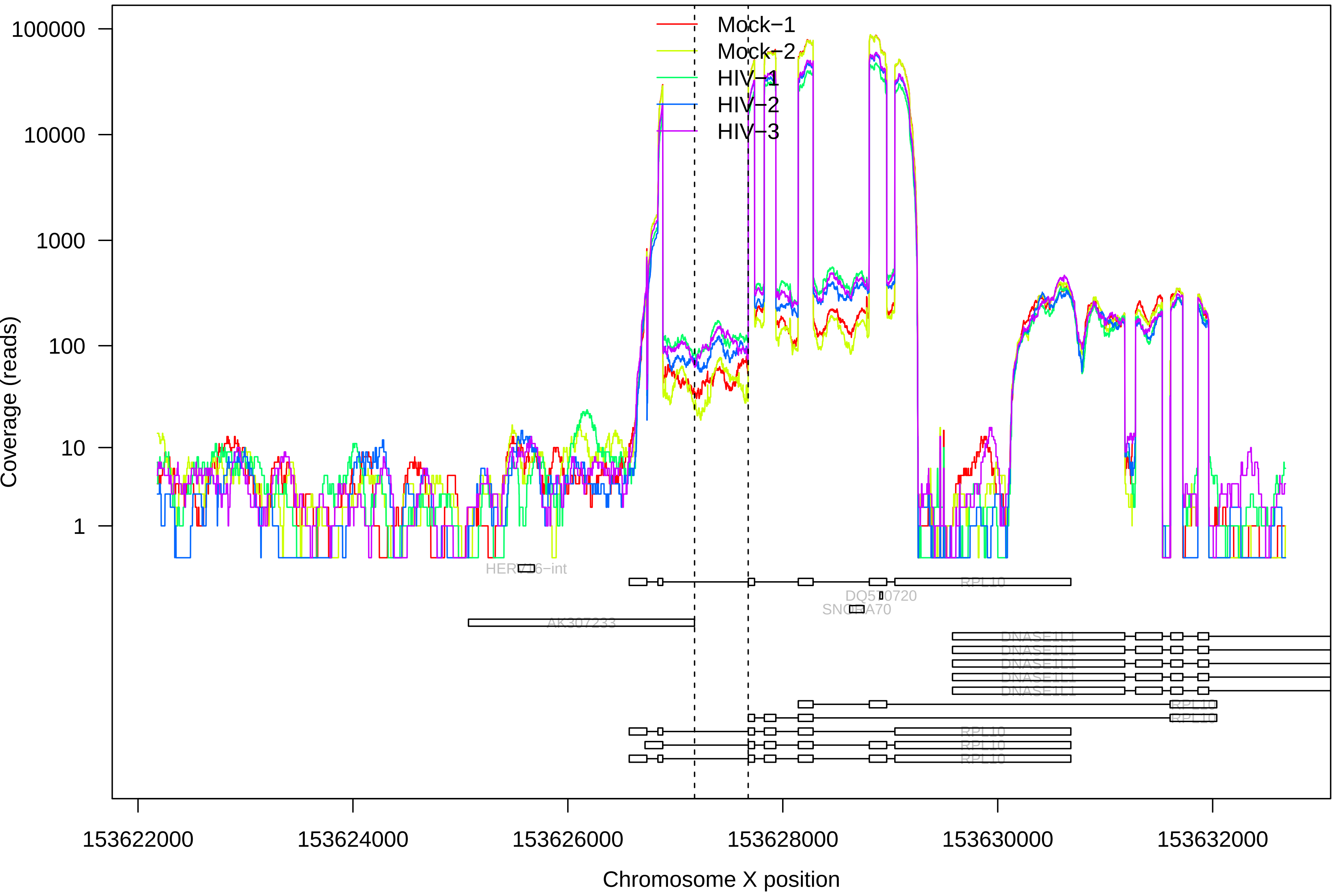
chrX:153627179−153627678
Mock−1
Mock−2
HIV−1
HIV−2
HIV−3
100000
10000
1000
100
10
1
Coverage (reads)
HERV16−int
RPL10
DQ570720
SNORA70
AK307233
DNASE1L1
153622000
153624000
153626000
153628000
153630000
153632000
Chromosome X position

A.40

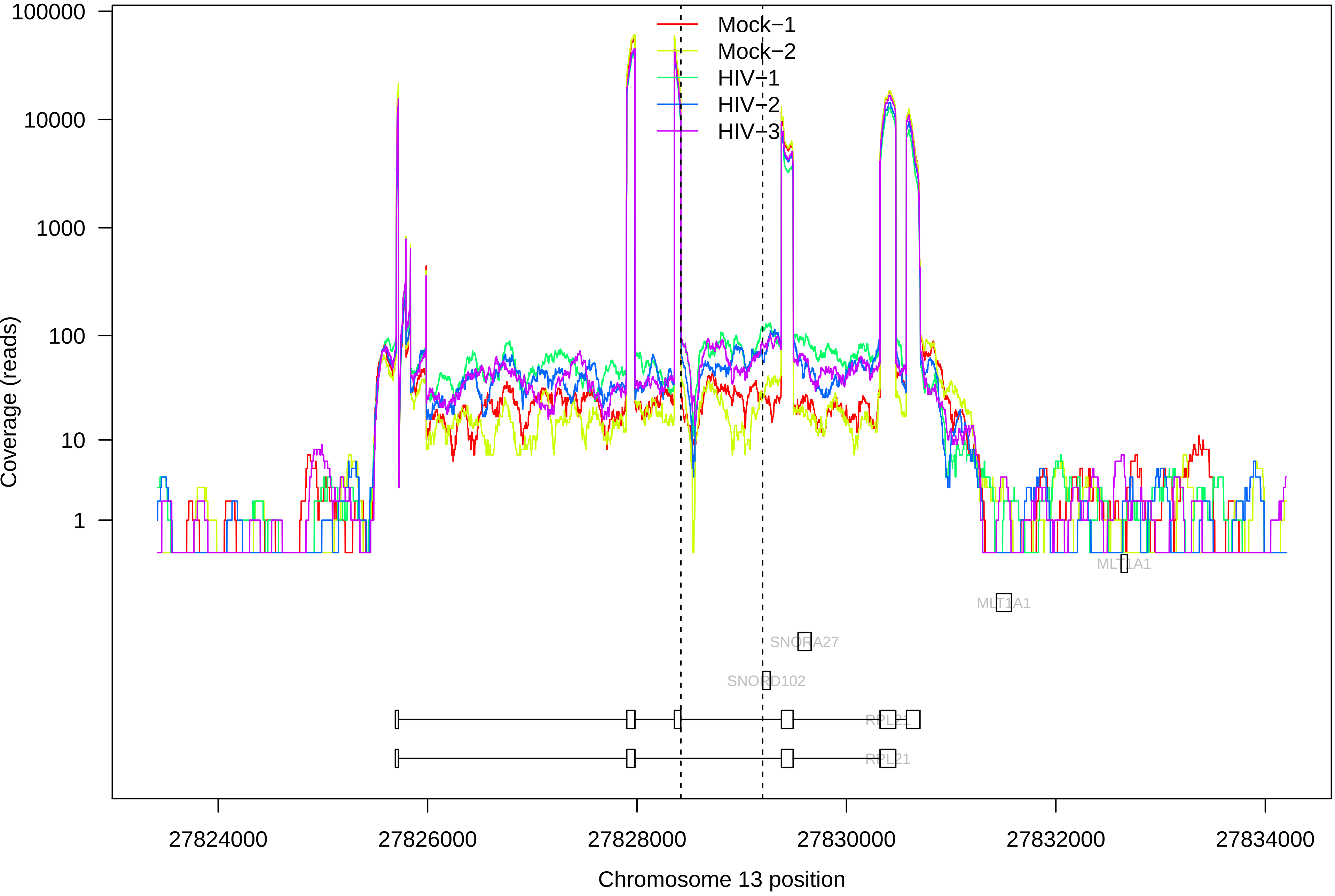
chr13:27828419−27829200
Mock−1
Mock−2
HIV−1
HIV−2
HIV−3
Coverage (reads)
100000
10000
1000
100
10
1
MLT1A1
MLT1A1
SNORA27
SNORD102
RPL21
RPL21
27824000
27826000
27828000
27830000
27832000
27834000
Chromosome 13 position

A.41

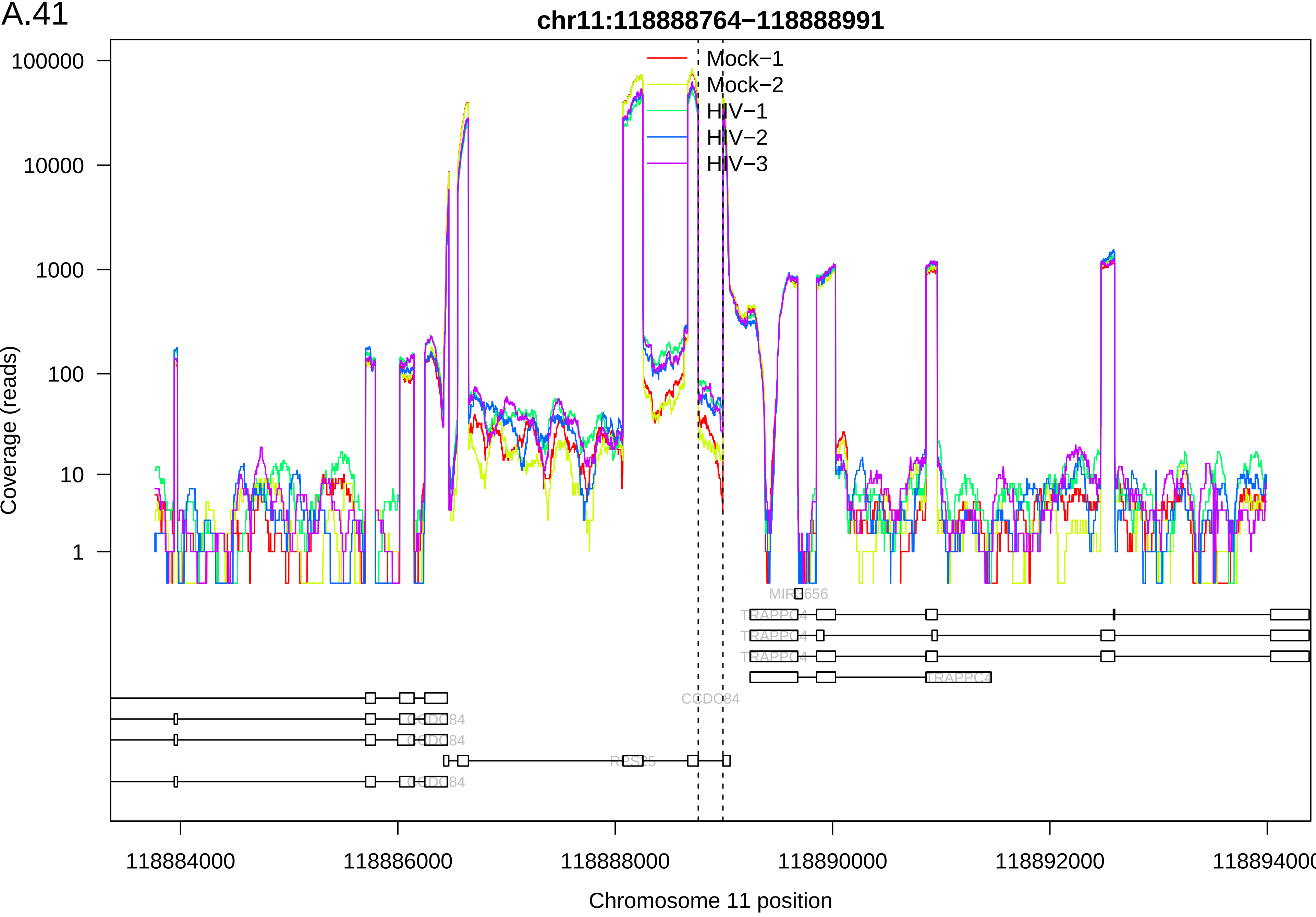
chr11:118888764−118888991
Mock−1
Mock−2
HIV−1
HIV−2
HIV−3
Coverage (reads)
100000
10000
1000
100
10
1
MIR4656
TRAPPC4
TRAPPC4
TRAPPC4
TRAPPC4
CCDC84
CCDC84
CCDC84
RPS25
CCDC84
118884000
118886000
118888000
118890000
118892000
118894000
Chromosome 11 position

A.42

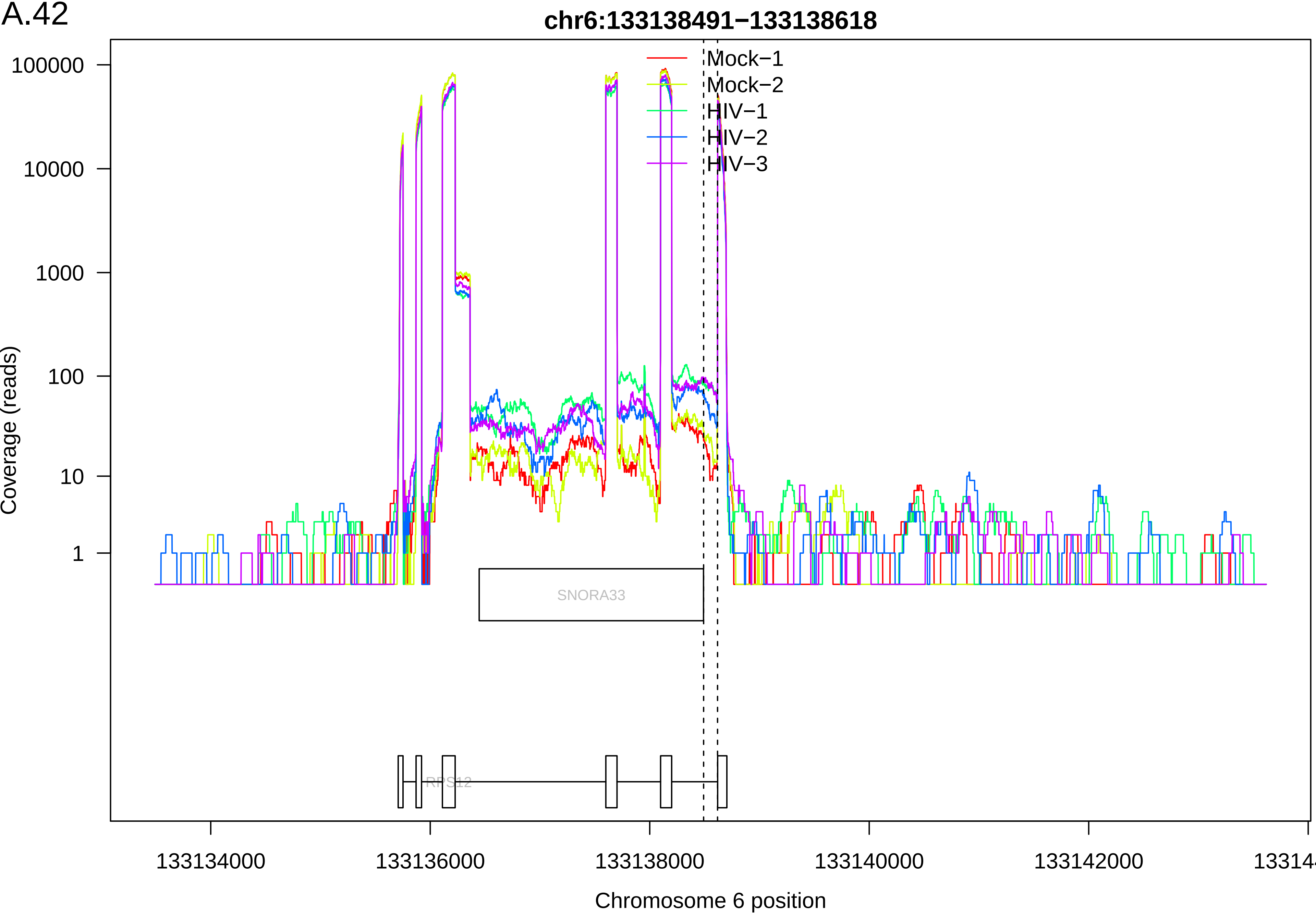

chr6:133138491−133138618
Mock−1
Mock−2
HIV−1
HIV−2
HIV−3
100000
10000
1000
100
10
1
Coverage (reads)
SNORA33
RPS12
133134000
133136000
133138000
133140000
133142000
Chromosome 6 position

A.43

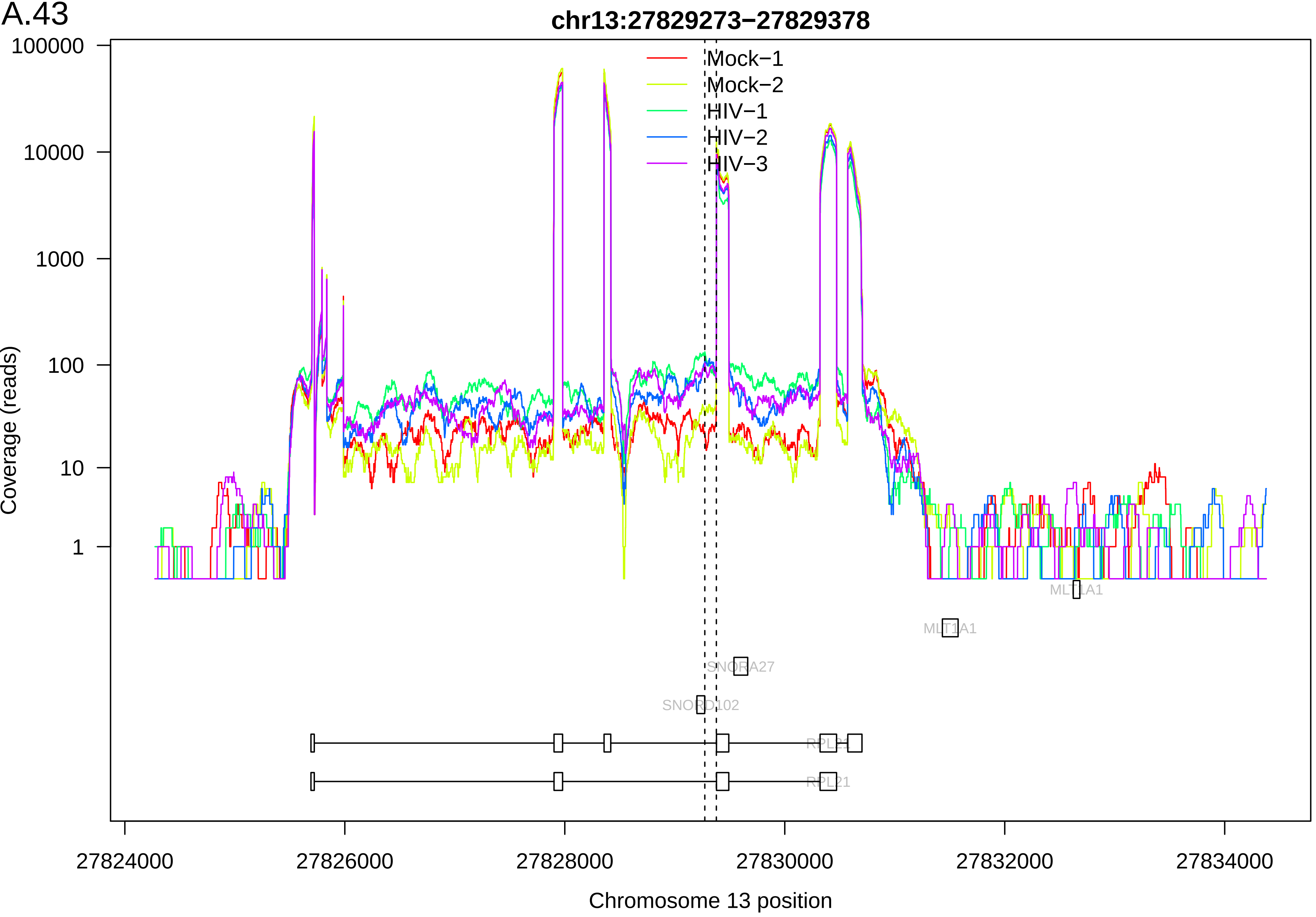
chr13:27829273−27829378
Mock−1
Mock−2
HIV−1
HIV−2
HIV−3
100000
10000
1000
100
10
1
Coverage (reads)
MLT1A1
MLT1A1
SNORA27
SNORD102
RPL21
RPL21
27824000
27826000
27828000
27830000
27832000
27834000
Chromosome 13 position

A.44

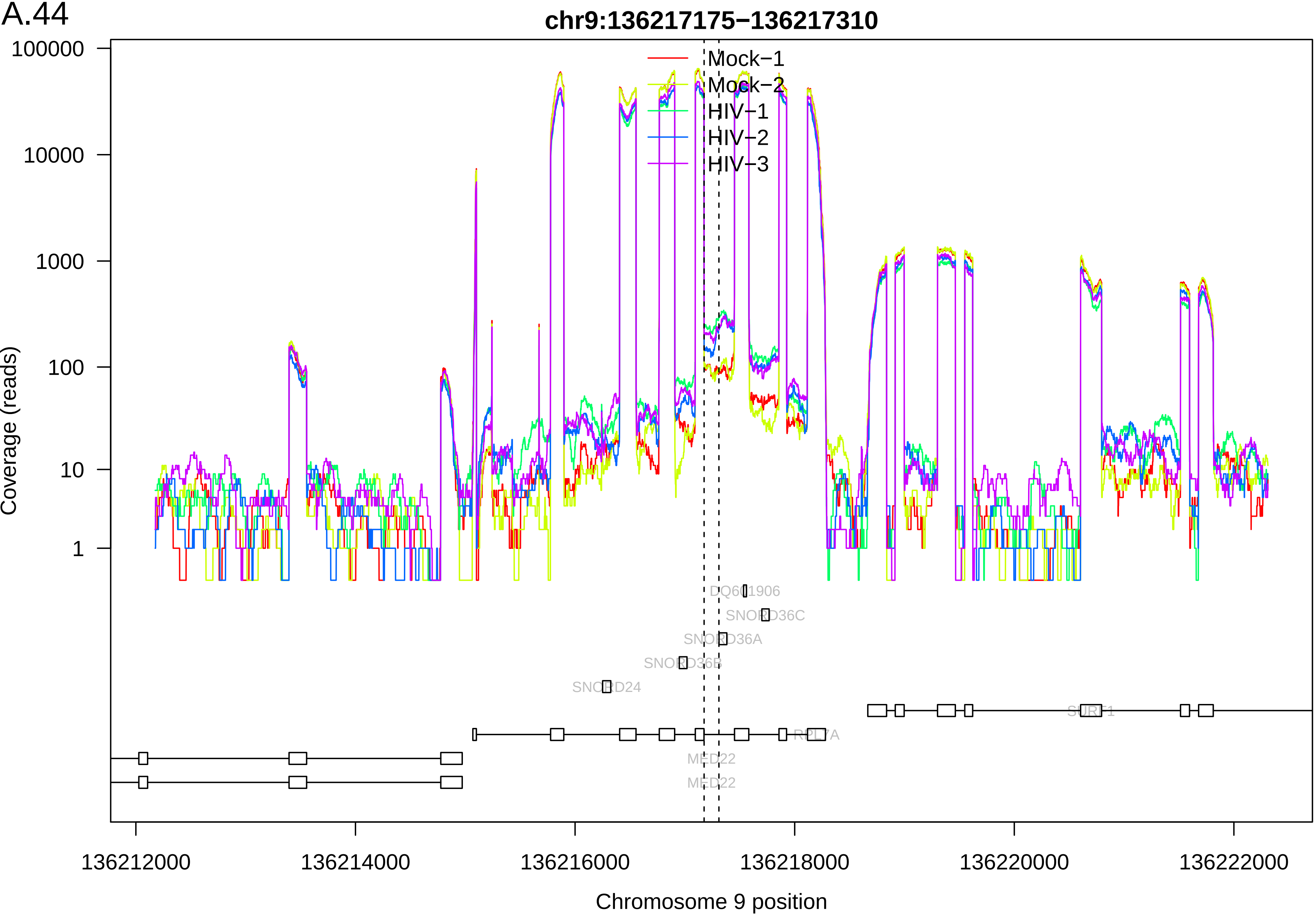
chr9:136217175−136217310
Mock−1
Mock−2
HIV−1
HIV−2
HIV−3
Coverage (reads)
100000
10000
1000
100
10
1
DQ601906
SNORD36C
SNORD36A
SNORD36B
SNORD24
SURF1
RPL7A
MED22
MED22
136212000
136214000
136216000
136218000
136220000
136222000
Chromosome 9 position

A.45

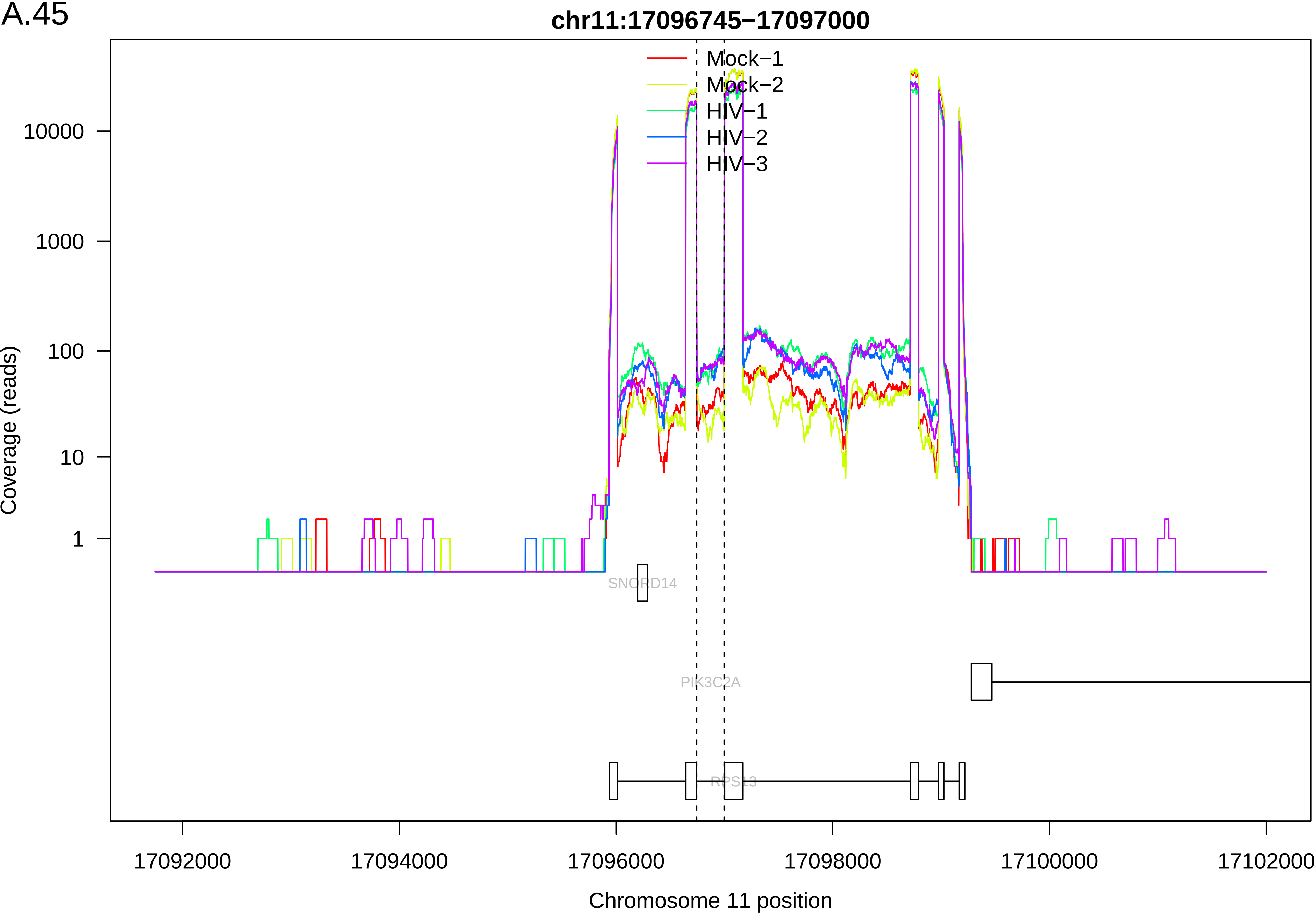

chr11:17096745−17097000
Mock−1
Mock−2
HIV−1
HIV−2
HIV−3
10000
1000
100
10
1
Coverage (reads)
SNORD14
PIK3C2A
RPS13
17092000
17094000
17096000
17098000
17100000
17102000
Chromosome 11 position

A.46

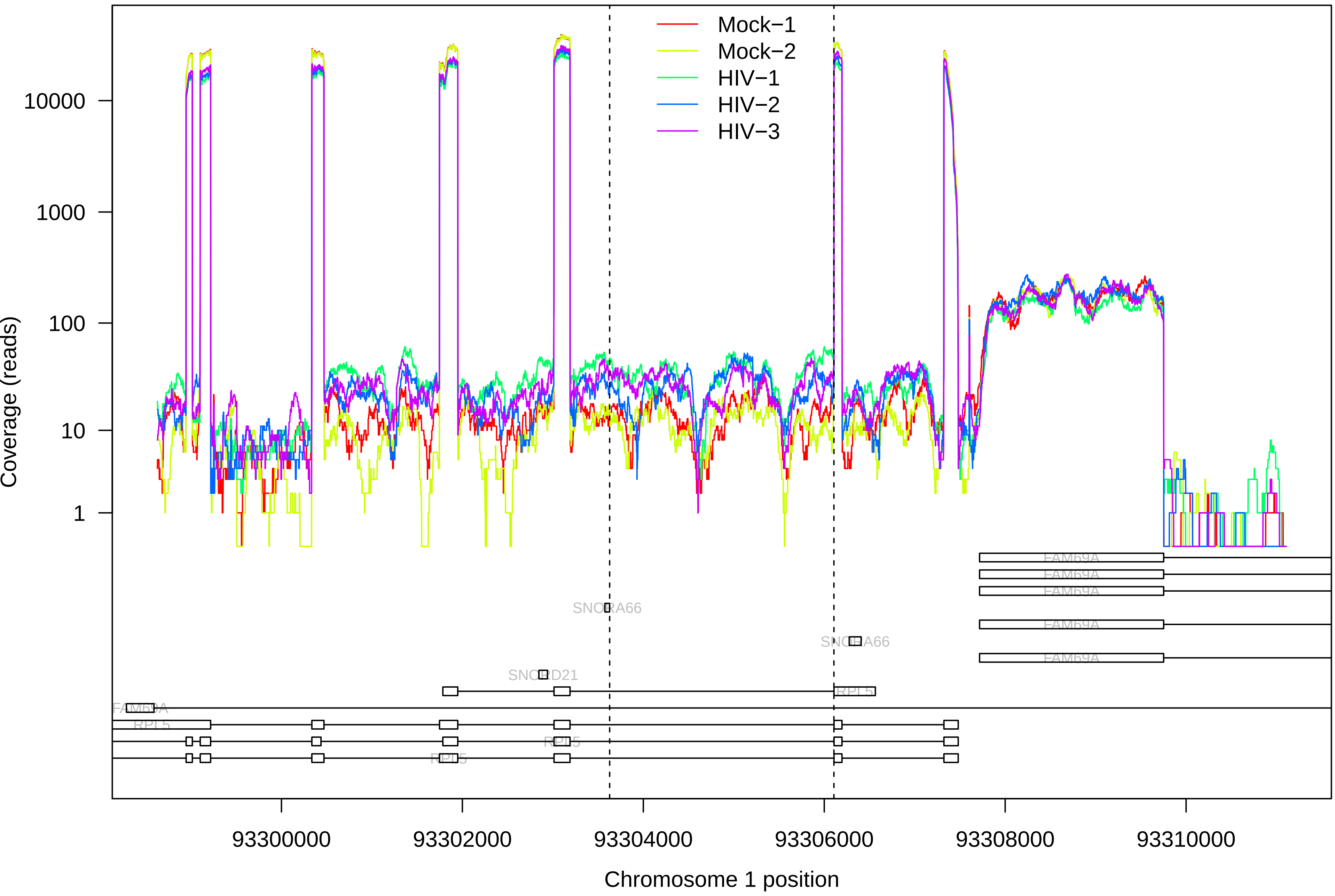

chr1:93303628−93306107
Mock−1
Mock−2
HIV−1
HIV−2
HIV−3
10000
1000
100
10
1
Coverage (reads)
FAM69A
FAM69A
FAM69A
FAM69A
FAM69A
SNORA66
SNORA66
SNORD21
RPL5
FAM69A
RPL5
RPL5
RPL5
93300000
93302000
93304000
93306000
93308000
93310000
Chromosome 1 position

A.47

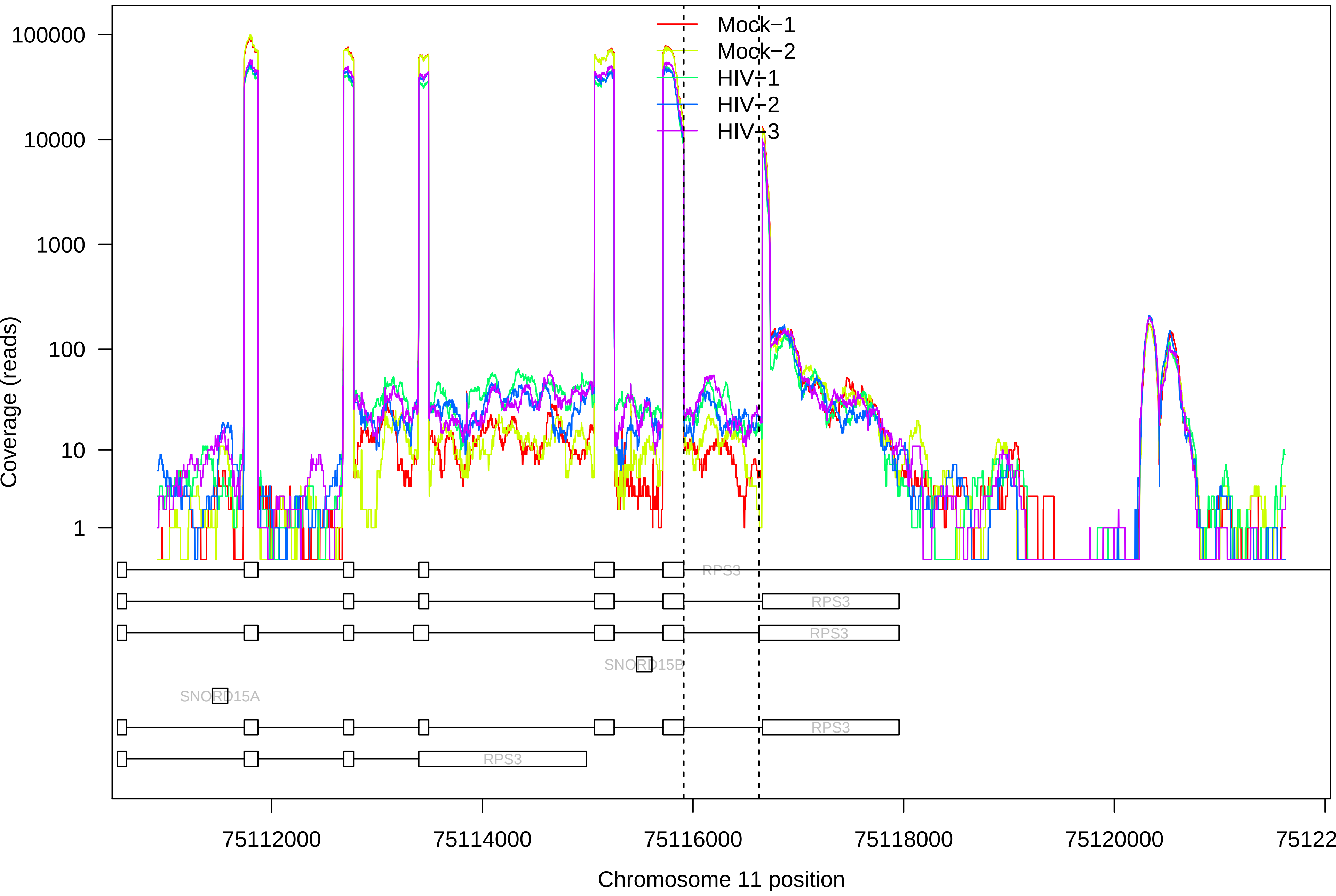
chr11:75115913−75116626
Mock−1
Mock−2
HIV−1
HIV−2
HIV−3
100000
10000
1000
100
10
1
Coverage (reads)
RPS3
RPS3
RPS3
SNORD15B
SNORD15A
RPS3
RPS3
75112000
75114000
75116000
75118000
75120000
75122
Chromosome 11 position

A.48

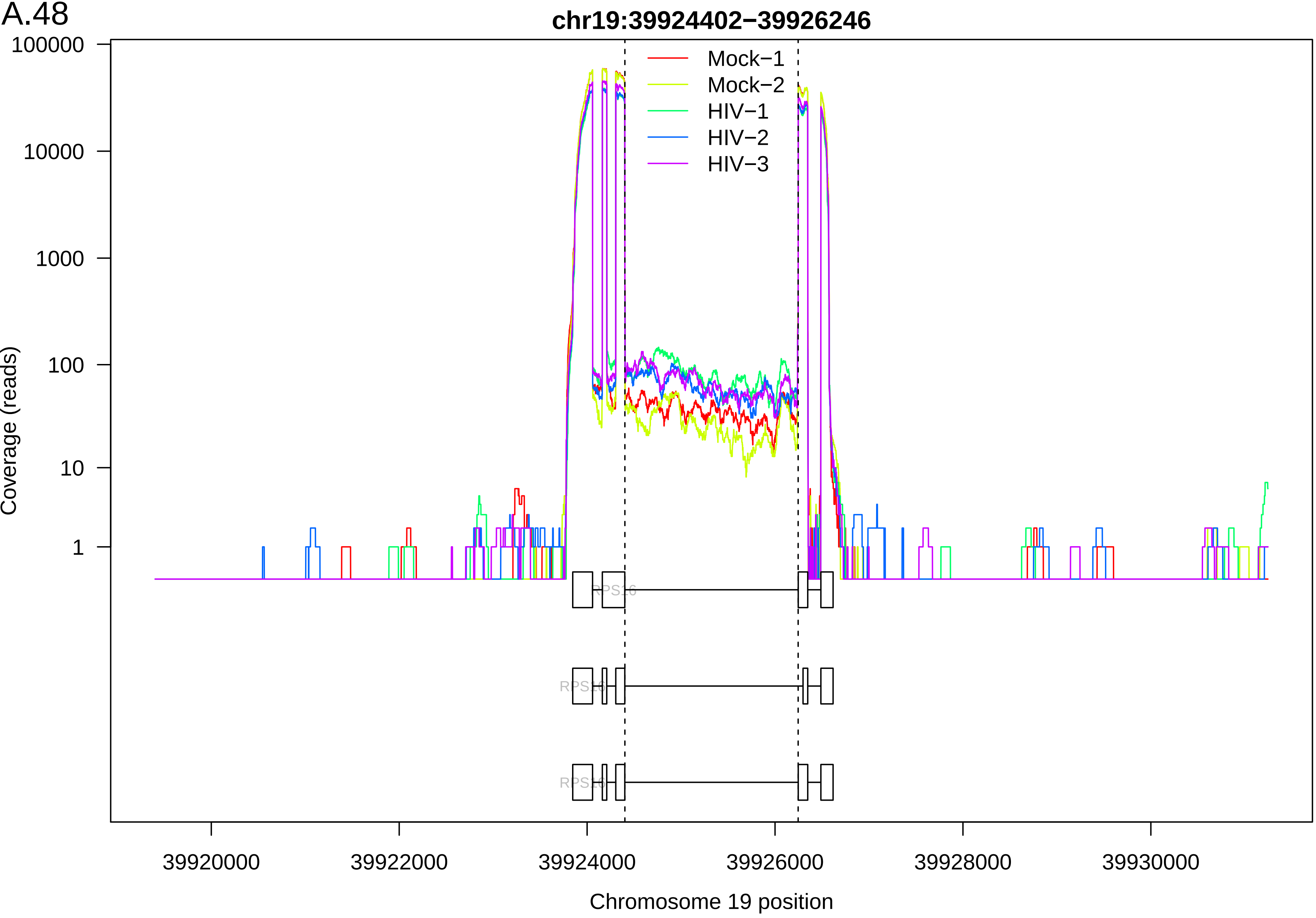

chr19:39924402−39926246
Mock−1
Mock−2
HIV−1
HIV−2
HIV−3
100000
10000
1000
100
10
1
Coverage (reads)
RPS16
RPS16
RPS16
39920000
39922000
39924000
39926000
39928000
39930000
Chromosome 19 position

A.49

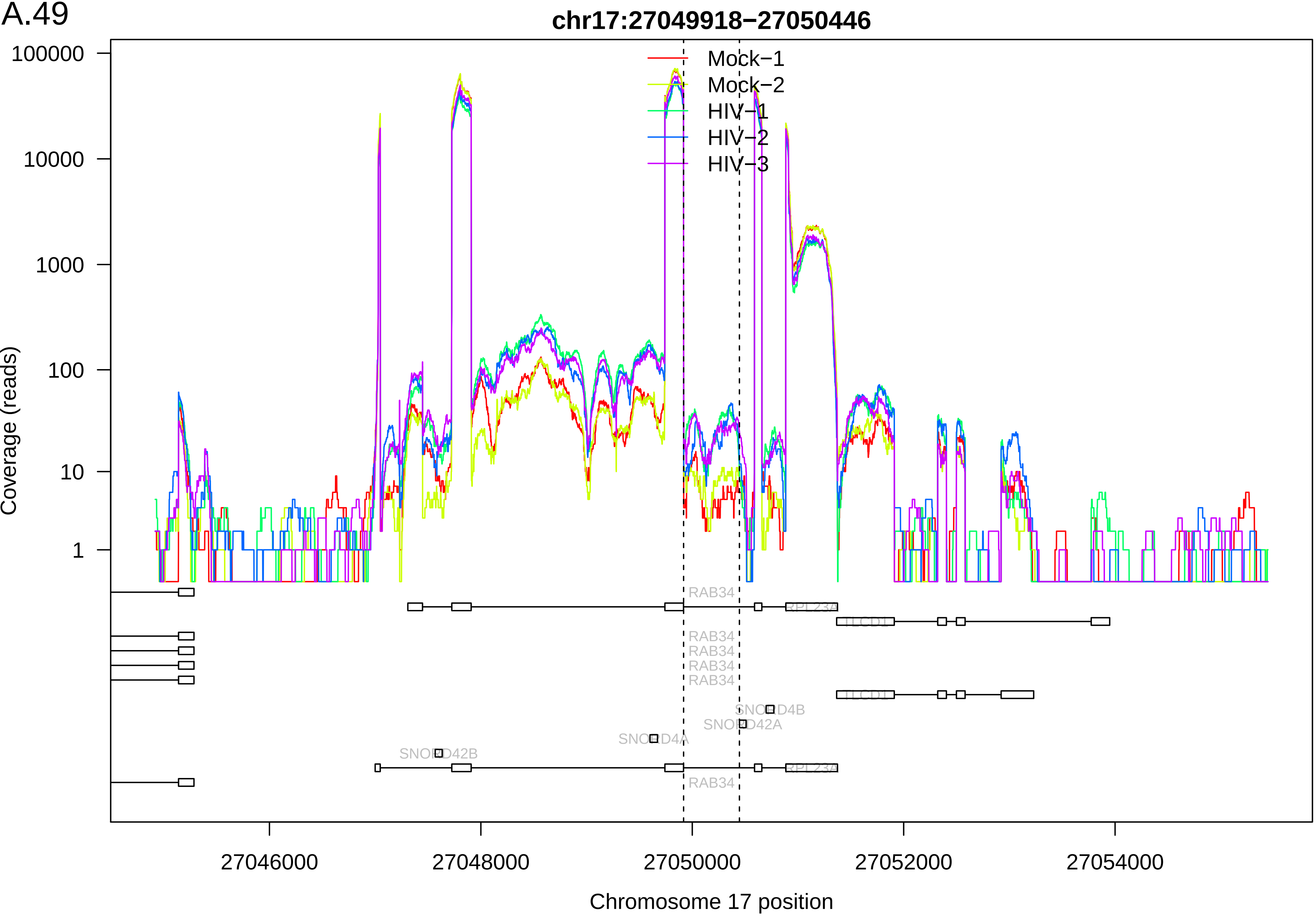
chr17:27049918−27050446
Mock−1
Mock−2
HIV−1
HIV−2
HIV−3
Coverage (reads)
100000
10000
1000
100
10
1
RAB34
RPL23A
TLCD1
SNORD4B
SNORD42A
SNORD4A
SNORD42B
27046000
27048000
27050000
27052000
27054000
Chromosome 17 position

A.50

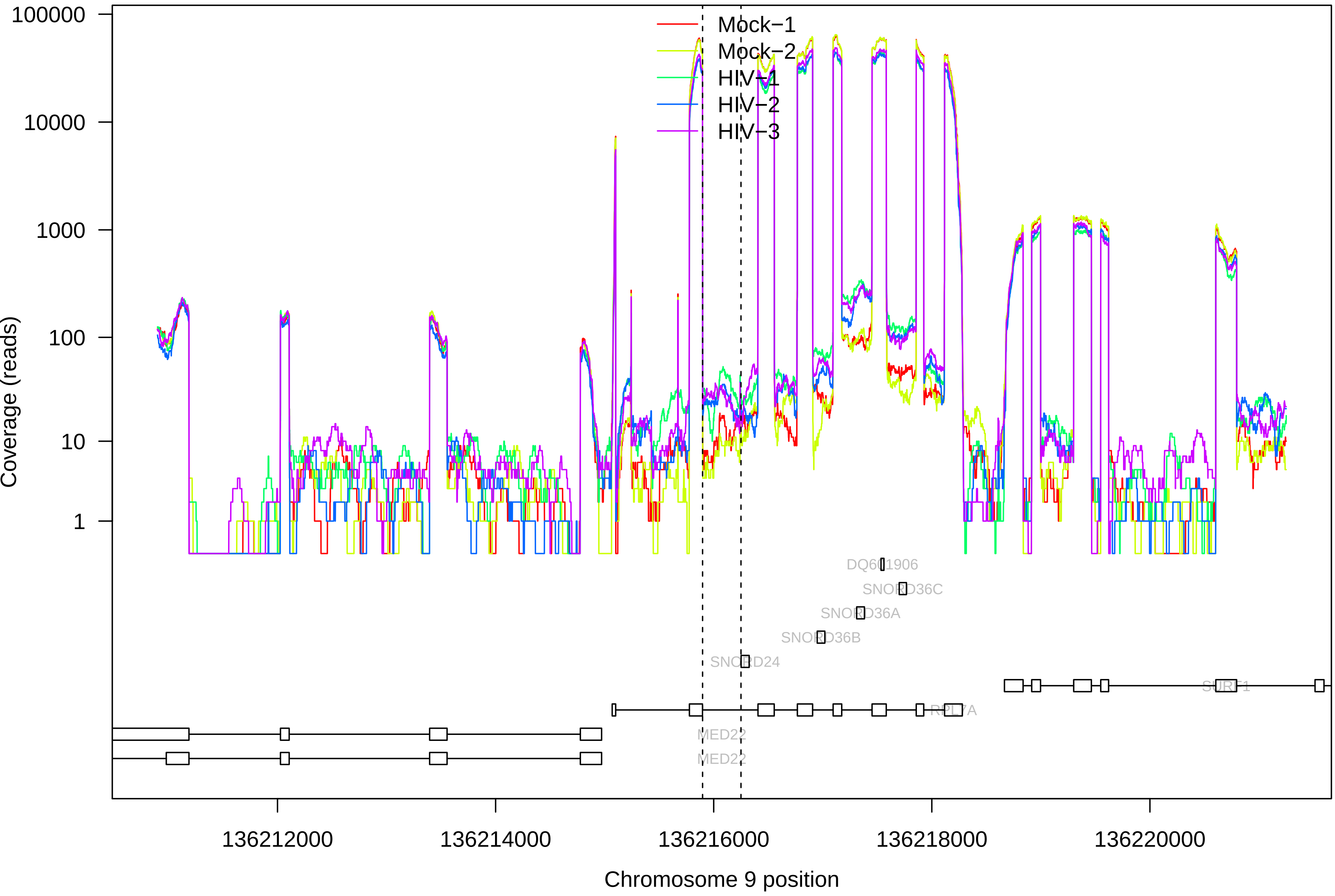
chr9:136215898−136216250
Mock−1
Mock−2
HIV−1
HIV−2
HIV−3
Coverage (reads)
100000
10000
1000
100
10
1
DQ601906
SNORD36C
SNORD36A
SNORD36B
SNORD24
SURF1
RPL7A
MED22
MED22
136212000
136214000
136216000
136218000
136220000
Chromosome 9 position

B.1

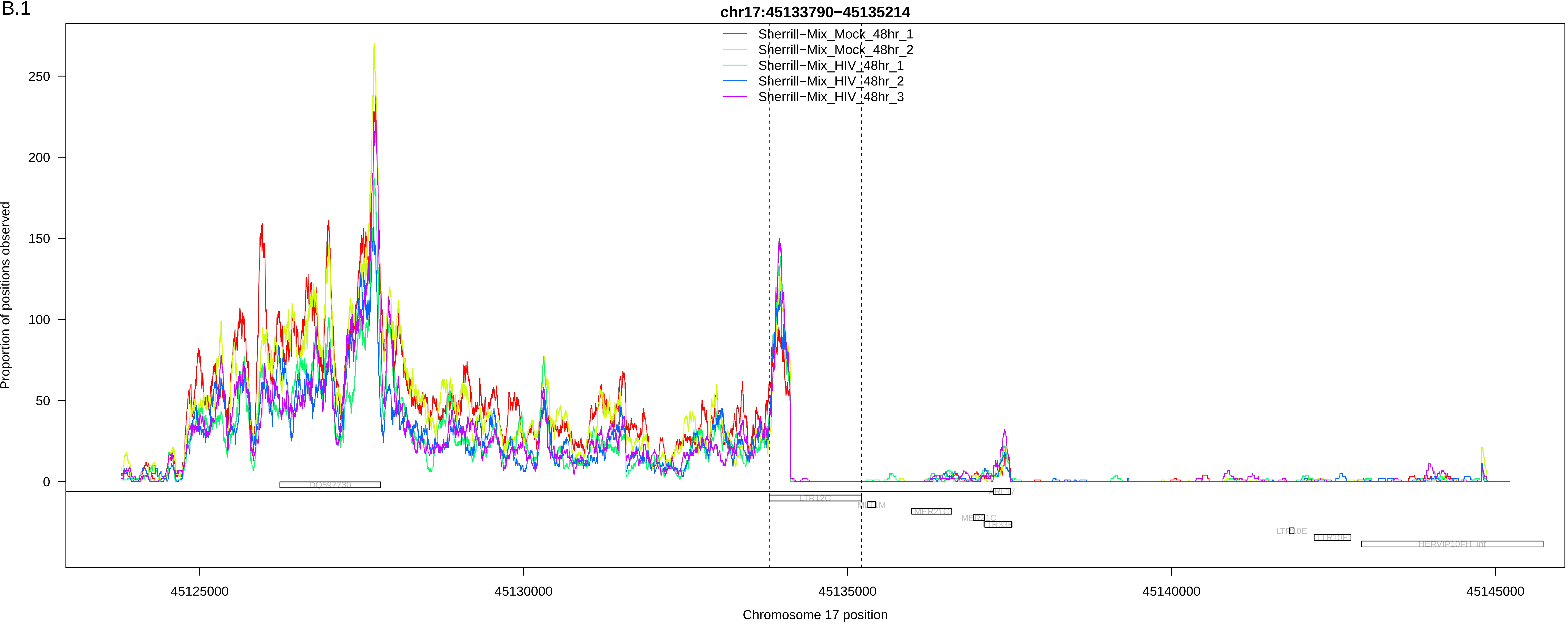

chr17:45133790−45135214
Sherrill−Mix_Mock_48hr_1
Sherrill−Mix_Mock_48hr_2
Sherrill−Mix_HIV_48hr_1
Sherrill−Mix_HIV_48hr_2
Sherrill−Mix_HIV_48hr_3
Proportion of positions observed
0
50
100
150
200
250
45125000
45130000
45135000
45140000
45145000
Chromosome 17 position

B.2

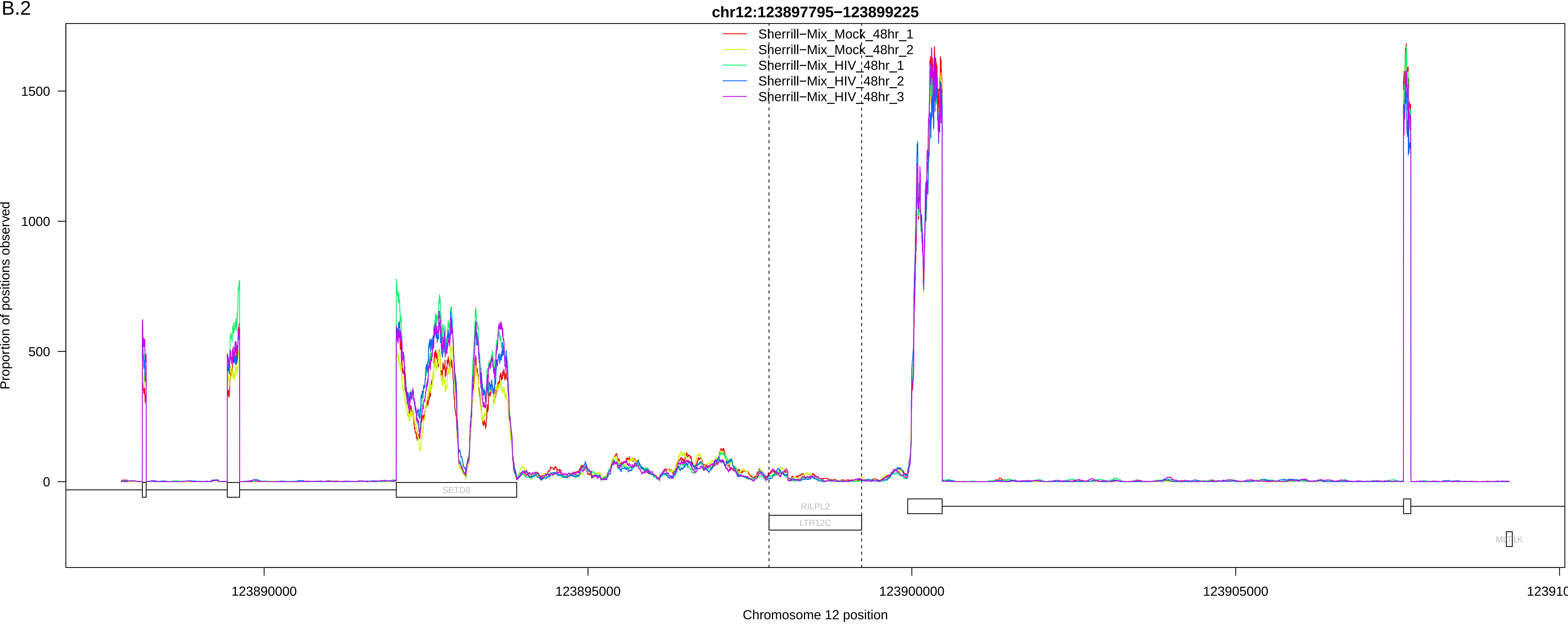

chr12:123897795−123899225
Sherrill−Mix_Mock_48hr_1
Sherrill−Mix_Mock_48hr_2
Sherrill−Mix_HIV_48hr_1
Sherrill−Mix_HIV_48hr_2
Sherrill−Mix_HIV_48hr_3
Proportion of positions observed
1500
1000
500
0
SETD8
RILPL2
LTR12C
123890000
123895000
123900000
123905000
Chromosome 12 position

B.3

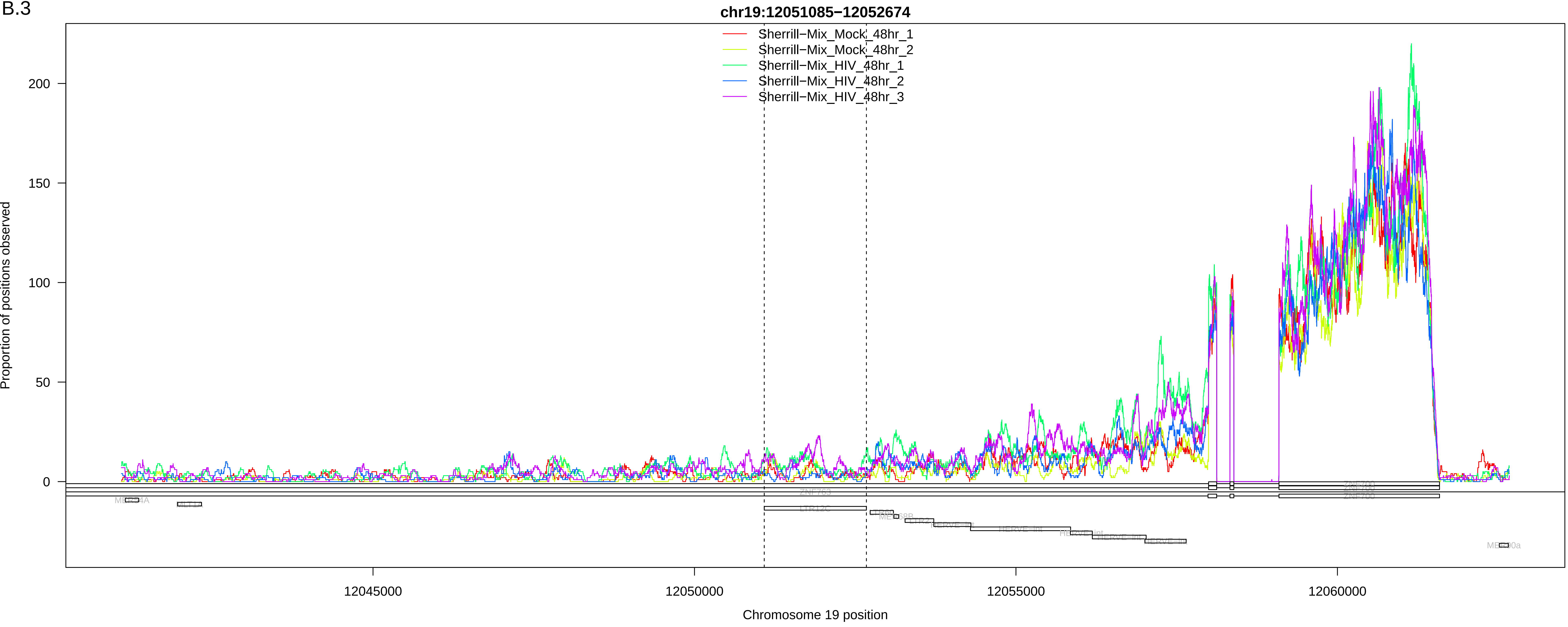

chr19:12051085−12052674
Sherrill−Mix_Mock_48hr_1
Sherrill−Mix_Mock_48hr_2
Sherrill−Mix_HIV_48hr_1
Sherrill−Mix_HIV_48hr_2
Sherrill−Mix_HIV_48hr_3
Proportion of positions observed
0
50
100
150
200
12045000
12050000
12055000
12060000
Chromosome 19 position

B.4

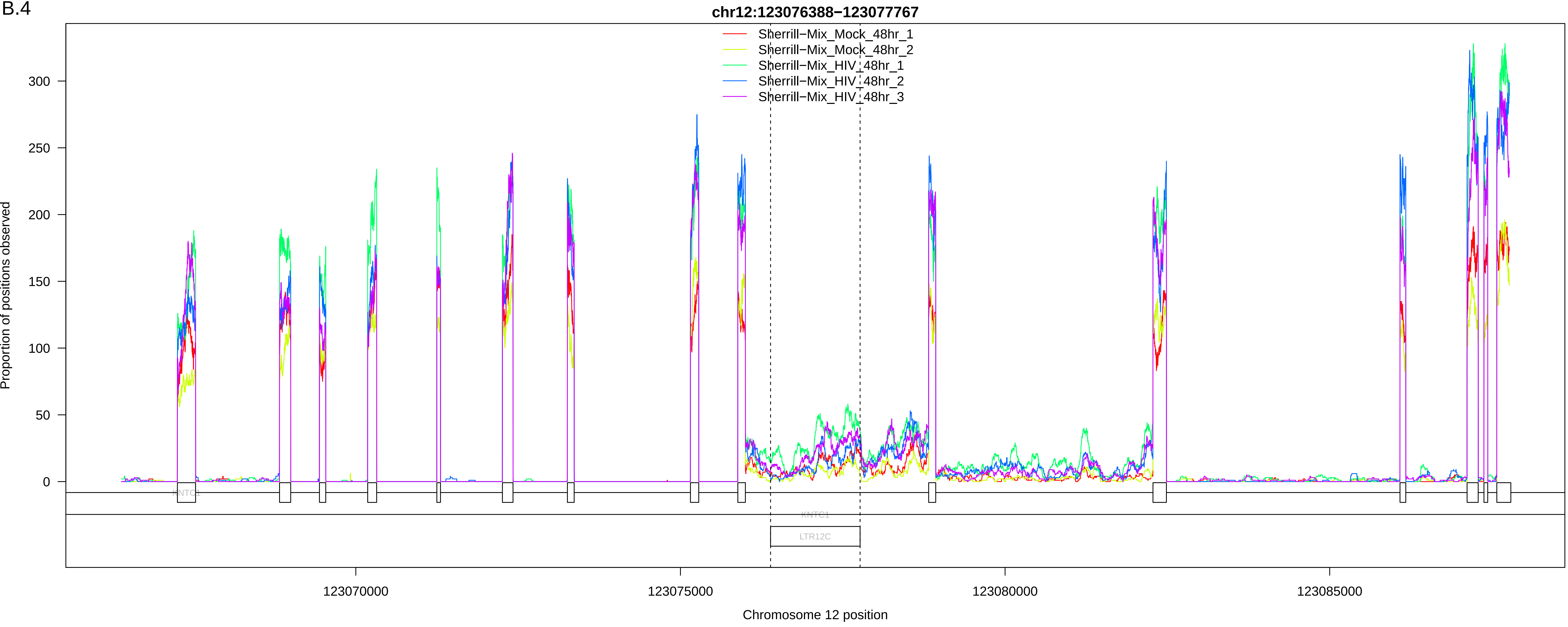

chr12:123076388−123077767
Sherrill−Mix_Mock_48hr_1
Sherrill−Mix_Mock_48hr_2
Sherrill−Mix_HIV_48hr_1
Sherrill−Mix_HIV_48hr_2
Sherrill−Mix_HIV_48hr_3
Proportion of positions observed
300
250
200
150
100
50
0
KNTC1
KNTC1
LTR12C
123070000
123075000
123080000
123085000
Chromosome 12 position

B.5

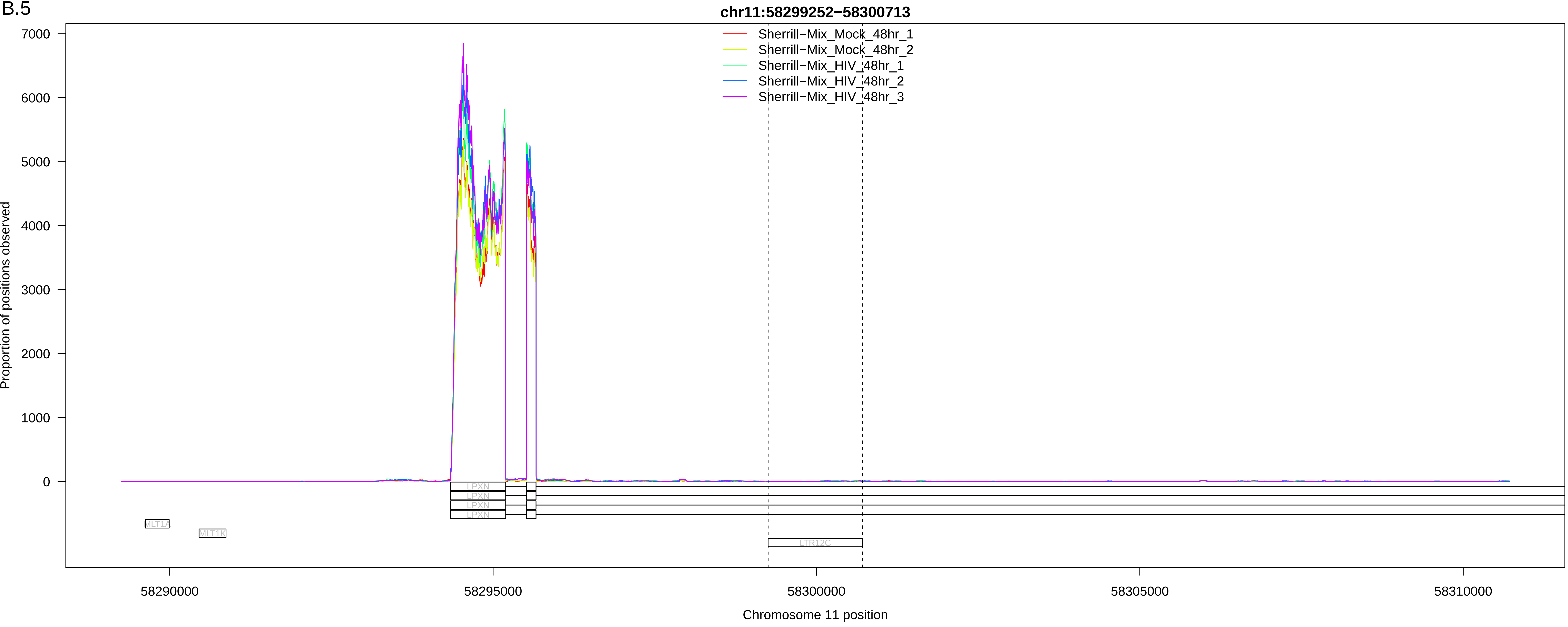

chr11:58299252−58300713
Sherrill−Mix_Mock_48hr_1
Sherrill−Mix_Mock_48hr_2
Sherrill−Mix_HIV_48hr_1
Sherrill−Mix_HIV_48hr_2
Sherrill−Mix_HIV_48hr_3
Proportion of positions observed
7000
6000
5000
4000
3000
2000
1000
0
LPXN
LPXN
LPXN
LPXN
MLT1A
MLT1K
LTR12C
58290000
58295000
58300000
58305000
58310000
Chromosome 11 position

B.6

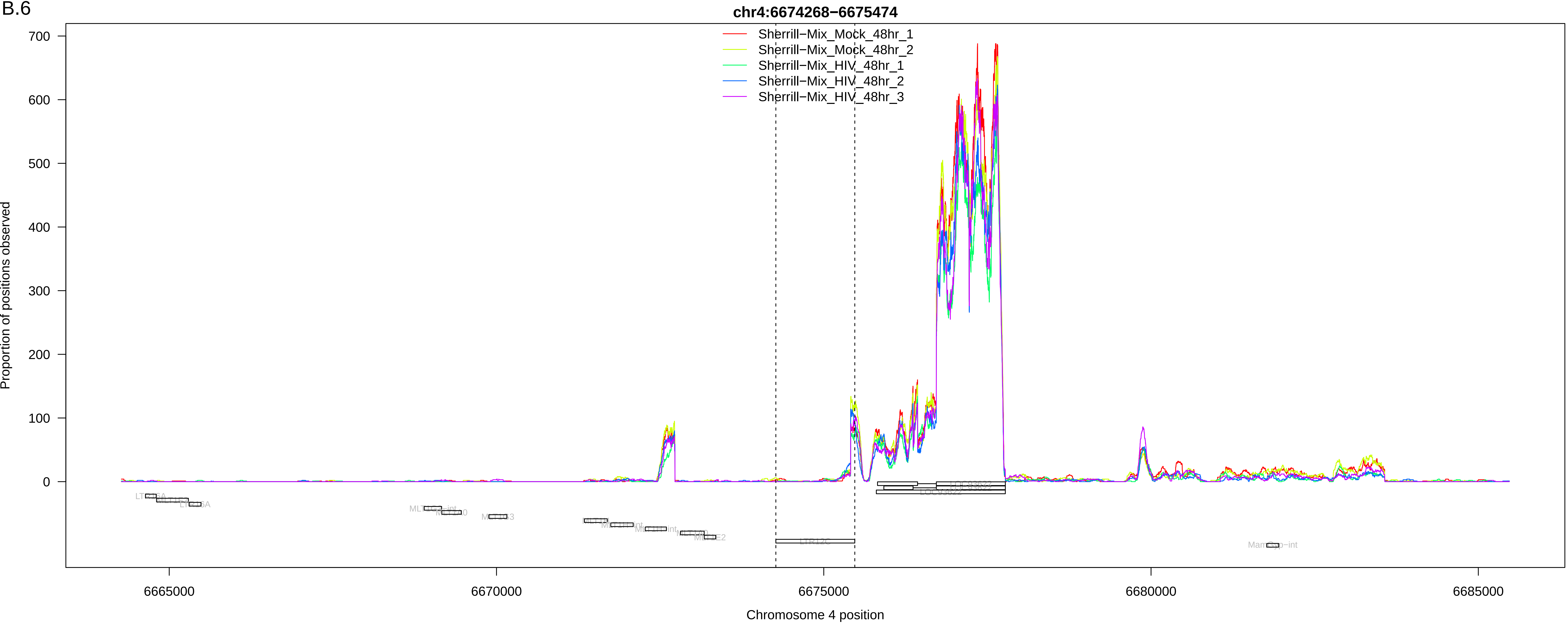

chr4:6674268−6675474
Sherrill−Mix_Mock_48hr_1
Sherrill−Mix_Mock_48hr_2
Sherrill−Mix_HIV_48hr_1
Sherrill−Mix_HIV_48hr_2
Sherrill−Mix_HIV_48hr_3
Proportion of positions observed
700
600
500
400
300
200
100
0
6665000
6670000
6675000
6680000
6685000
Chromosome 4 position

B.7

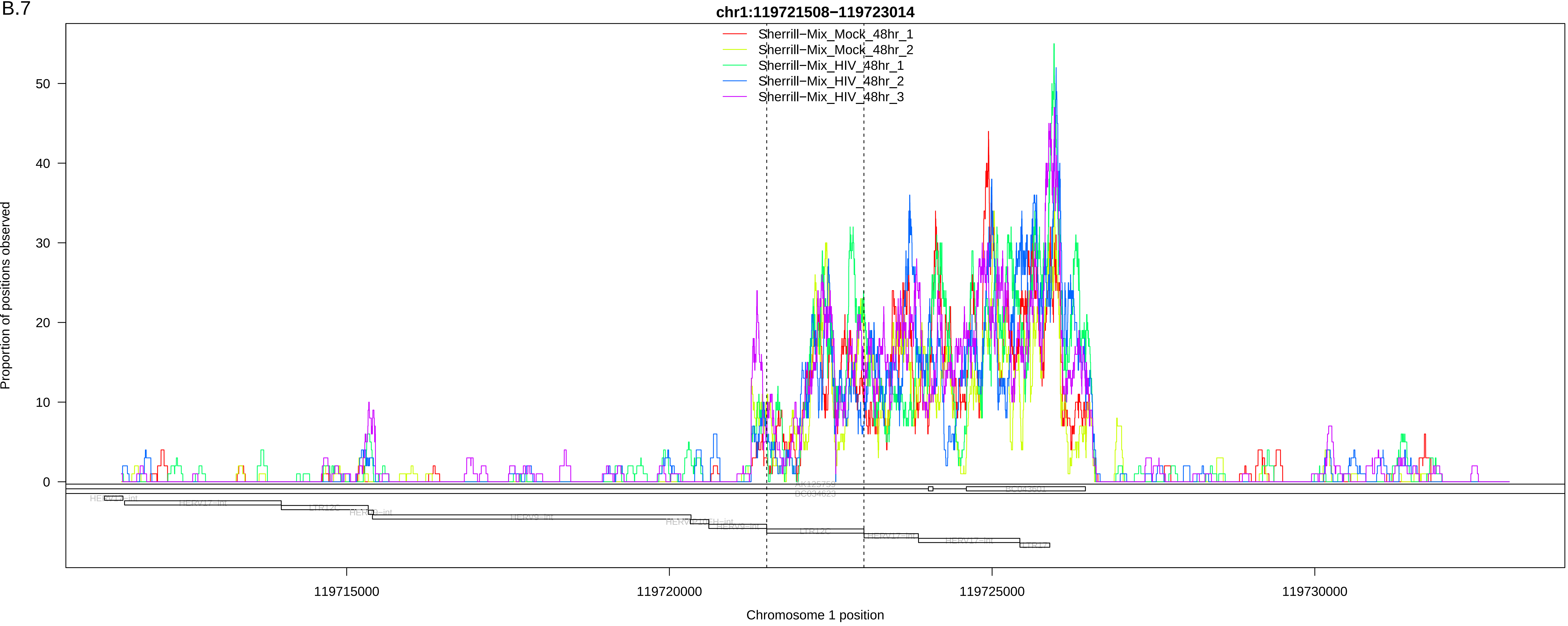
chr1:119721508−119723014
Sherrill−Mix_Mock_48hr_1
Sherrill−Mix_Mock_48hr_2
Sherrill−Mix_HIV_48hr_1
Sherrill−Mix_HIV_48hr_2
Sherrill−Mix_HIV_48hr_3
Proportion of positions observed
0
10
20
30
40
50
119715000
119720000
119725000
119730000
Chromosome 1 position

B.8

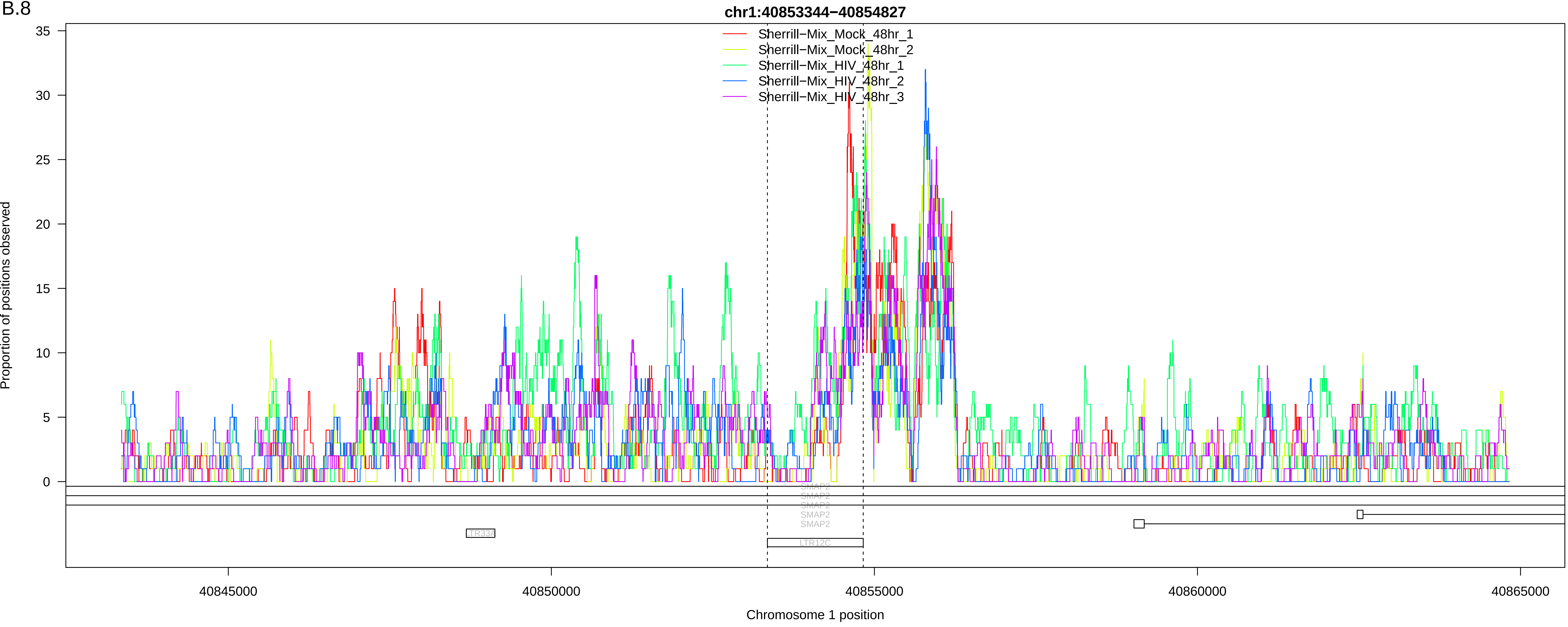
chr1:40853344−40854827
Sherrill−Mix_Mock_48hr_1
Sherrill−Mix_Mock_48hr_2
Sherrill−Mix_HIV_48hr_1
Sherrill−Mix_HIV_48hr_2
Sherrill−Mix_HIV_48hr_3
Proportion of positions observed
0
5
10
15
20
25
30
35
SMAP2
SMAP2
SMAP2
SMAP2
SMAP2
LTR33A
LTR12C
40845000
40850000
40855000
40860000
40865000
Chromosome 1 position

B.9

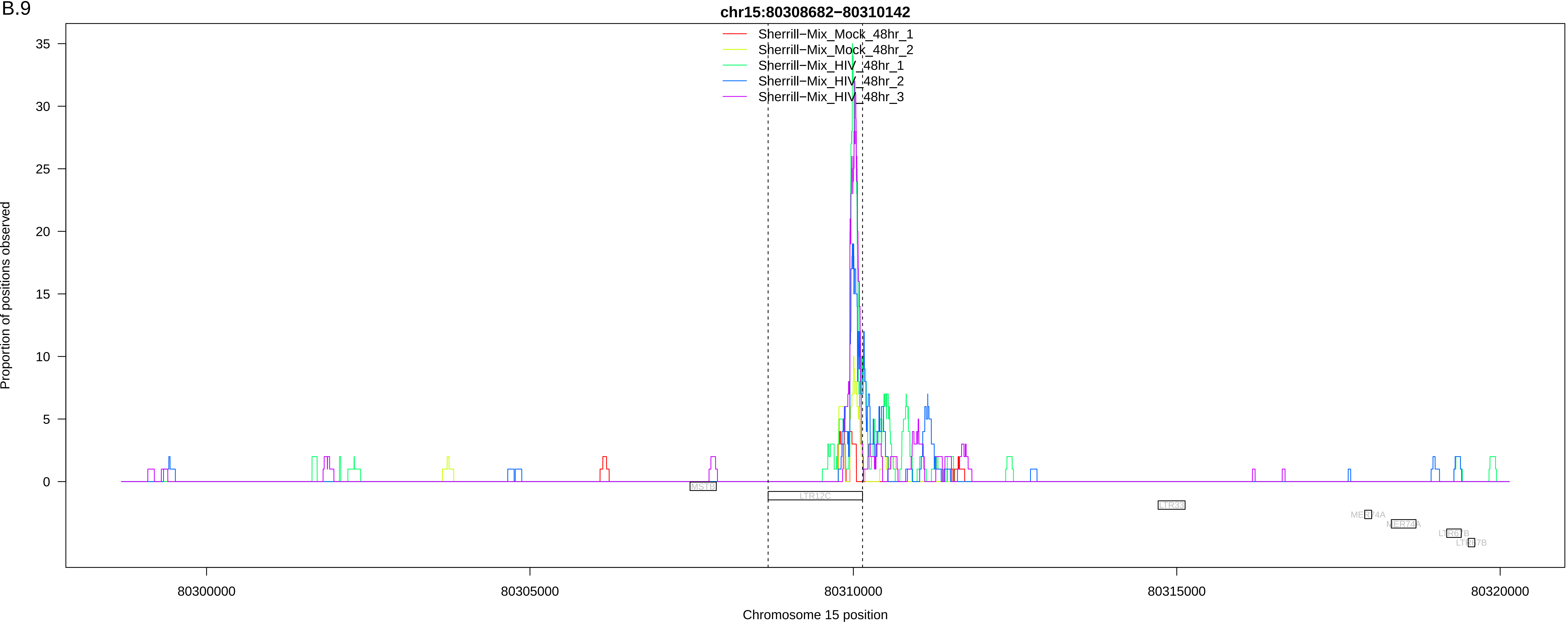

chr15:80308682−80310142
Sherrill−Mix_Mock_48hr_1
Sherrill−Mix_Mock_48hr_2
Sherrill−Mix_HIV_48hr_1
Sherrill−Mix_HIV_48hr_2
Sherrill−Mix_HIV_48hr_3
Proportion of positions observed
0
5
10
15
20
25
30
35
MSTB
LTR12C
LTR33
MER74A
MER74A
80300000
80305000
80310000
80315000
80320000
Chromosome 15 position

B.10

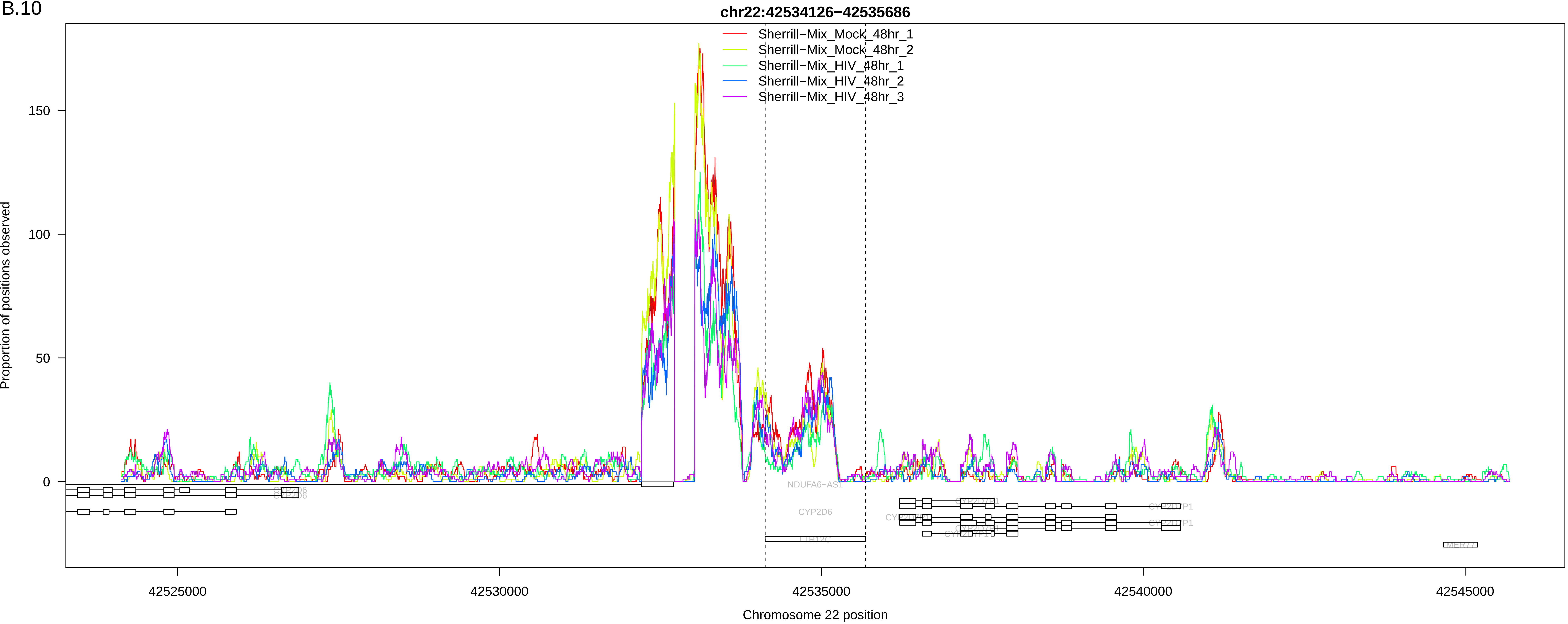

chr22:42534126−42535686
Sherrill−Mix_Mock_48hr_1
Sherrill−Mix_Mock_48hr_2
Sherrill−Mix_HIV_48hr_1
Sherrill−Mix_HIV_48hr_2
Sherrill−Mix_HIV_48hr_3
Proportion of positions observed
0
50
100
150
42525000
42530000
42535000
42540000
42545000
Chromosome 22 position
NDUFA6−AS1
CYP2D6

B.11

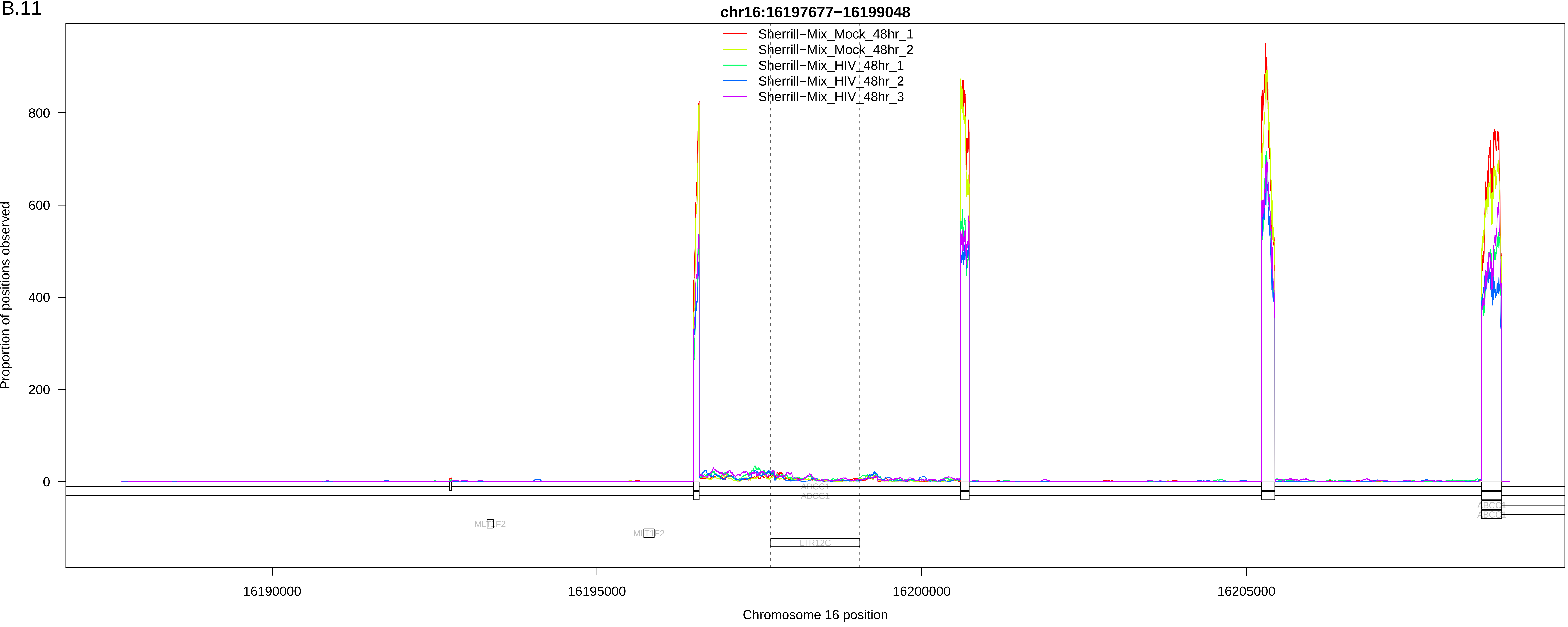
chr16:16197677−16199048
Sherrill−Mix_Mock_48hr_1
Sherrill−Mix_Mock_48hr_2
Sherrill−Mix_HIV_48hr_1
Sherrill−Mix_HIV_48hr_2
Sherrill−Mix_HIV_48hr_3
Proportion of positions observed
800
600
400
200
0
ABCC1
ABCC1
MLT1F2
MLT1F2
LTR12C
16190000
16195000
16200000
16205000
Chromosome 16 position

B.12

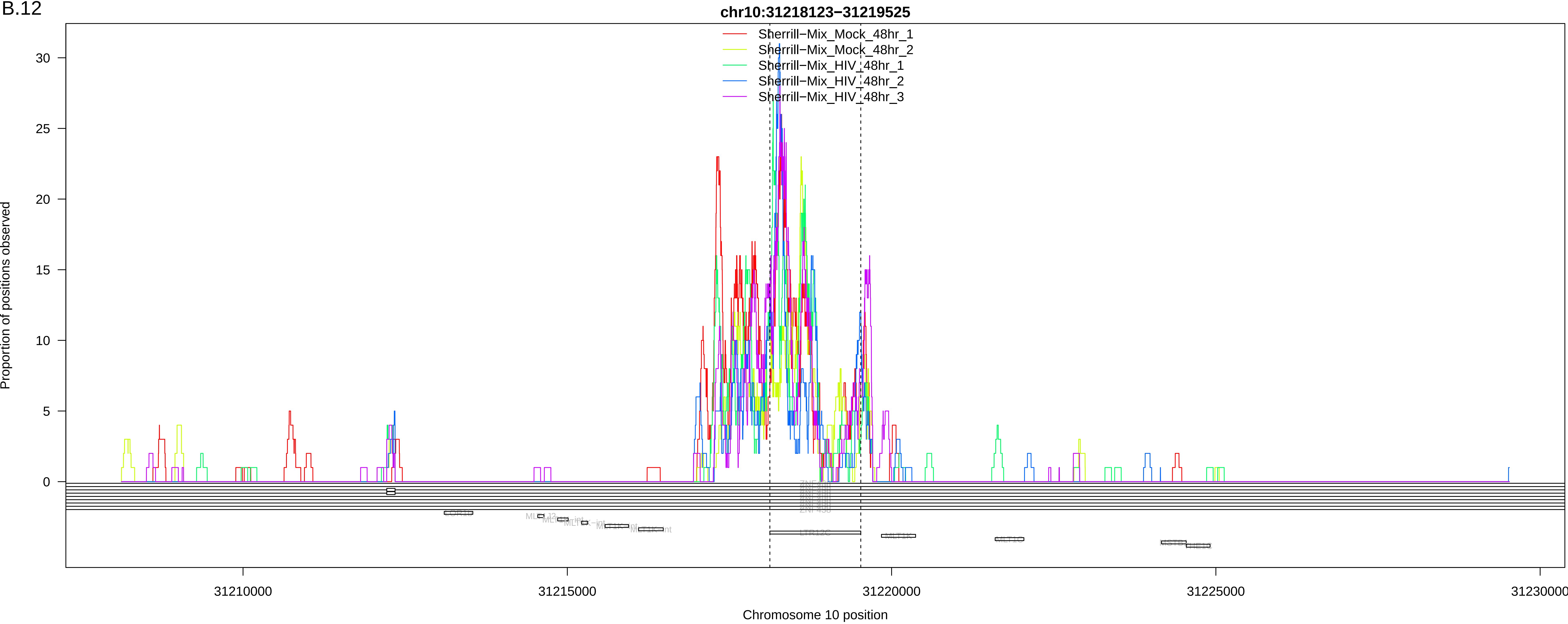
chr10:31218123−31219525
Sherrill−Mix_Mock_48hr_1
Sherrill−Mix_Mock_48hr_2
Sherrill−Mix_HIV_48hr_1
Sherrill−Mix_HIV_48hr_2
Sherrill−Mix_HIV_48hr_3
Proportion of positions observed
0
5
10
15
20
25
30
31210000
31215000
31220000
31225000
31230000
Chromosome 10 position

B.13

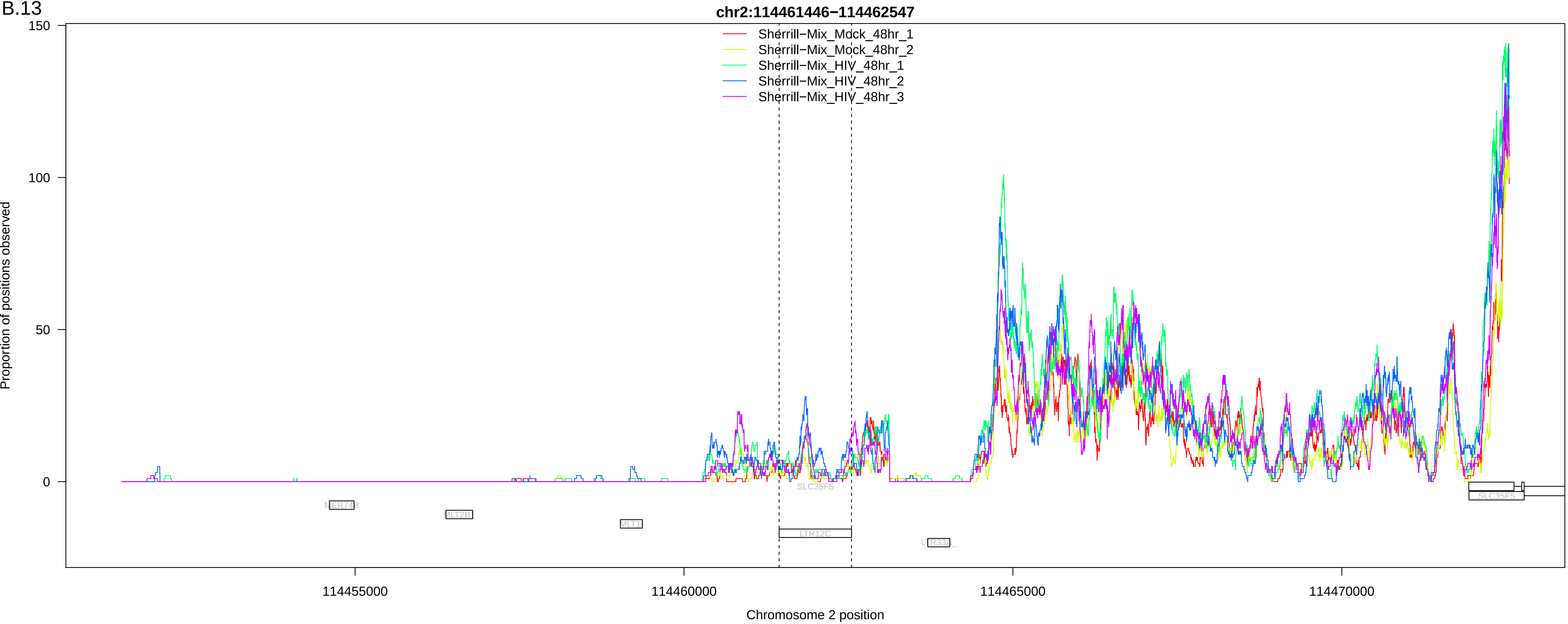

chr2:114461446−114462547
Sherrill−Mix_Mock_48hr_1
Sherrill−Mix_Mock_48hr_2
Sherrill−Mix_HIV_48hr_1
Sherrill−Mix_HIV_48hr_2
Sherrill−Mix_HIV_48hr_3
150
100
50
0
Proportion of positions observed
SLC35F5
LTR12C
MLT1
MLT2B
SLC35F5
114455000
114460000
114465000
114470000
Chromosome 2 position

B.14

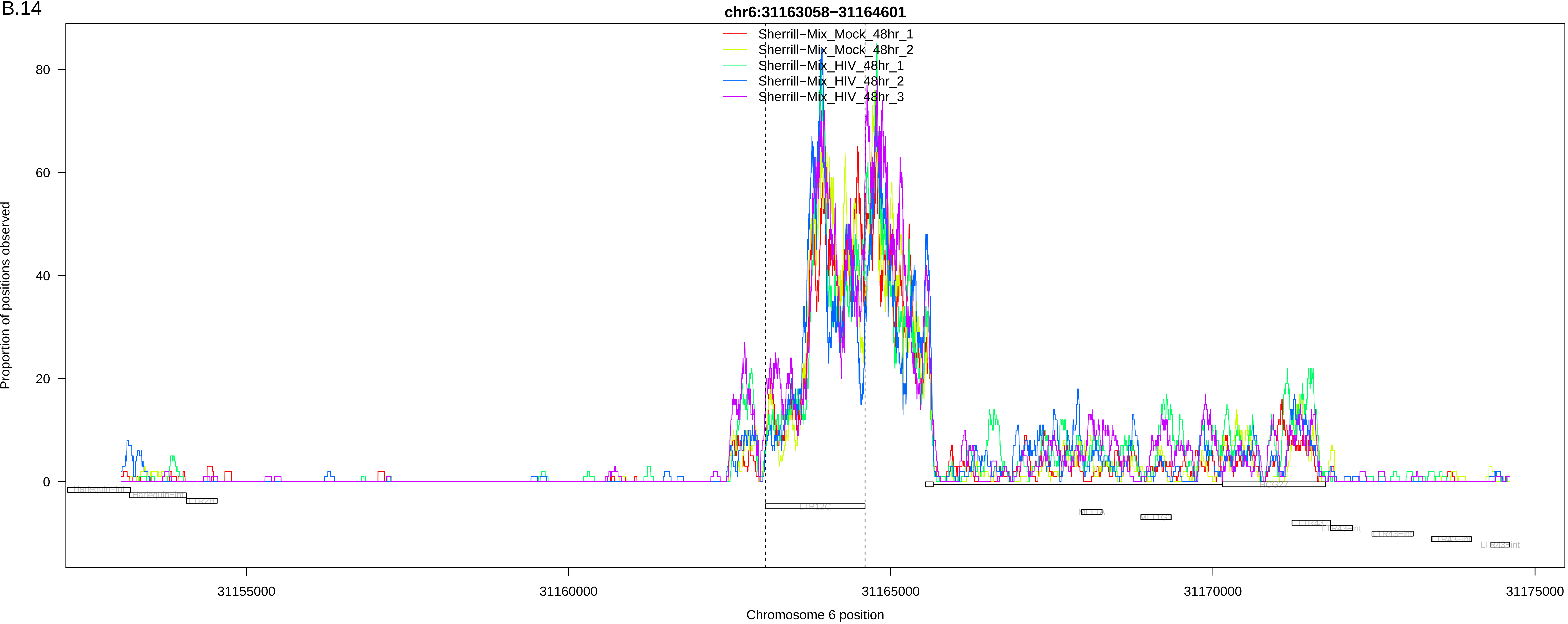
chr6:31163058−31164601
Sherrill−Mix_Mock_48hr_1
Sherrill−Mix_Mock_48hr_2
Sherrill−Mix_HIV_48hr_1
Sherrill−Mix_HIV_48hr_2
Sherrill−Mix_HIV_48hr_3
Proportion of positions observed
80
60
40
20
0
31155000
31160000
31165000
31170000
31175000
Chromosome 6 position
