# Supplementary figures and images for: Gene activity in primary T cells infected with HIV89.6: intron retention and induction of genomic repeats
Source: Retrovirology. 2015 Sep 17;12:79. doi: 10.1186/s12977-015-0205-1 (PMC4574318; doi:10.1186/s12977-015-0205-1)

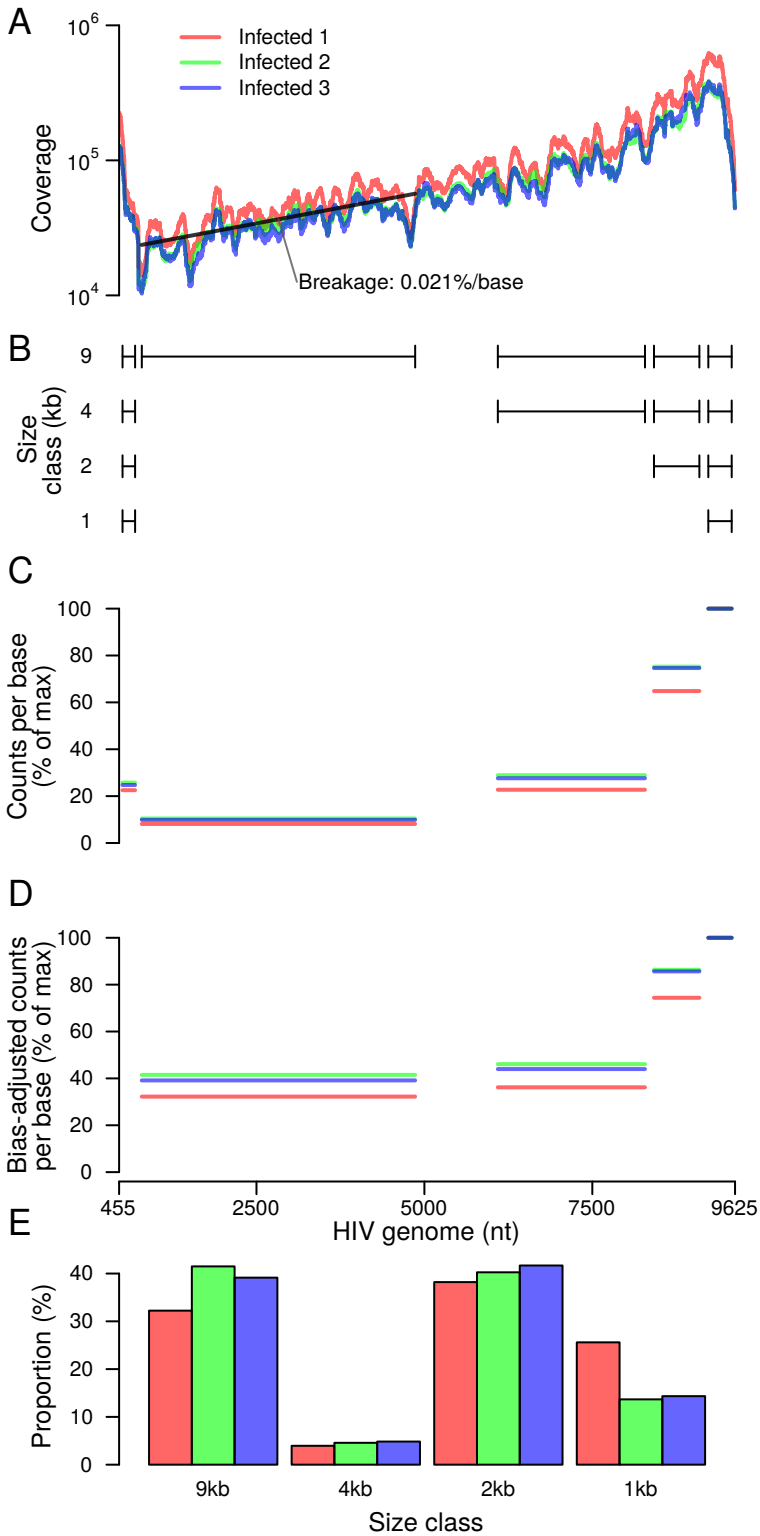

Supplement: Supplementary file 6 — 10.1186/s12977-015-0205-1 Estimating relative abundance of HIV 89.6 message size classes using RNA-Seq data. A) RNA-Seq coverage of the HIV89.6 genome for the replicates in this study. Each replicate is indicated by a different color. The HIV genome is shown on the x-axis and the number of reads that aligned to each position is shown on the y-axis. Black line indicates the 0.021 % coverage decrease per base distance from the 3′ end of the mRNA estimated from a least squares fit on the read counts in the first intron. B) Diagram of the segments of the HIV89.6 RNA present in each of 9 kb, 4 kb, 2 kb and 1 kb size class. C) The proportion of reads mapped to each of the segments of the HIV89.6 genome shown in B adjusted by the length of the segment. Each replicate is shown by a different color. D) Corrected representation of RNA segments from the different size classes. Because cDNA synthesis was primed from the polyA tail, more 3′ sequences are recovered preferentially. Using the bias estimate from A, we adjusted each genome segment by the inverse of the bias predicted based on its distance from the 3′ end of the mRNA. Corrected proportions for the indicated RNA segments are shown colored by replicate. The first exon can not be adjusted using this method since multiple lengths of messages will include it. E) The proportion of each size class was inferred using the estimates in D by calculating the difference between segments. Replicates are indicated by color. [file 12977_2015_205_MOESM6_ESM.pdf]

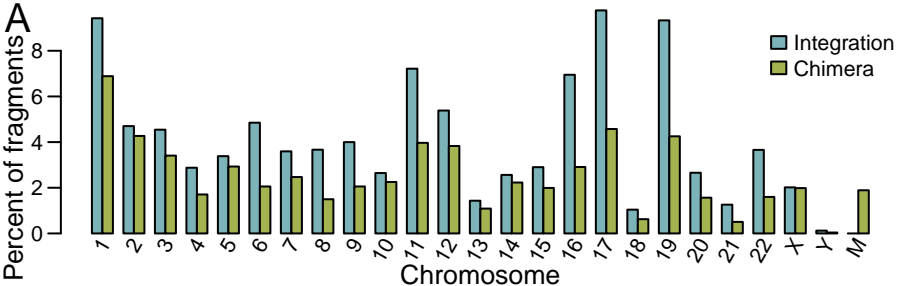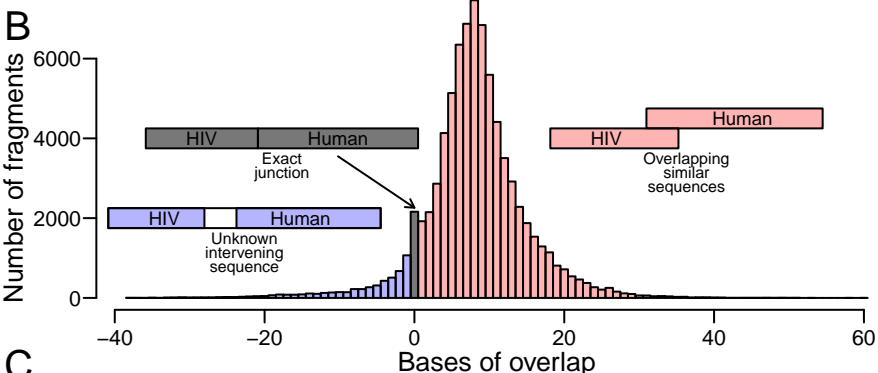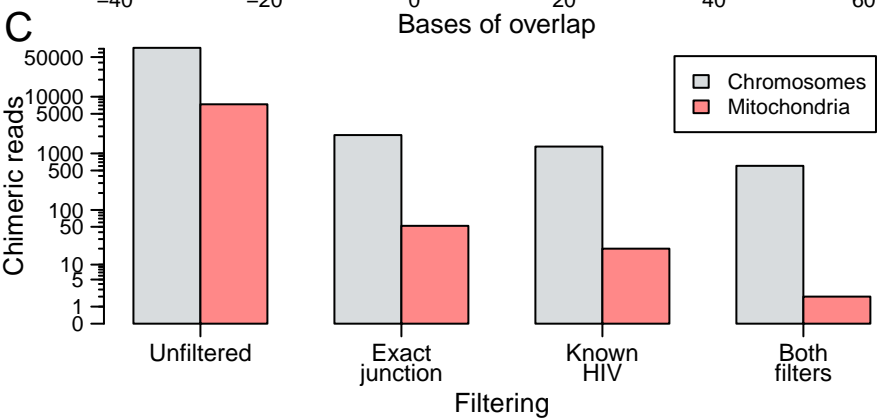

Supplement: Supplementary file 7 — 10.1186/s12977-015-0205-1 Comparison with integration site data reveals an abundance of artifactual chimeras. A) Chromosomal distribution of uniquely mapping HIV integration sites from the same infections of primary T cells and comparison to uniquely mapping human sequences in chimeric reads observed in RNA-Seq. Note that the mitochondrial genome, denoted as M, has no authentic integration sites but does have extensive matches to chimeric junctions found in the RNA-Seq data. B) The length of overlapping sequence (regions of complementarity potentially favoring chimera formation) matching both human and HIV at inferred chimeric junctions. The x-axis shows the length of the overlap and the y-axis shows the frequency of chimeric junctions with the indicated extent of overlap. C) The effects of filtering for chimeras with 1) an exact junction between human and HIV sequences with no unknown or overlapping sequence, 2) HIV segments starting at the 5′ or 3′ end of HIV or at known HIV splice sites or 3) both filters combined. A marked reduction in mitochondrial chimeras (inferred to be markers of sequencing artifacts) is observed with each filtering step. [file 12977_2015_205_MOESM7_ESM.pdf]
